# The generative capacity of probabilistic protein sequence models

**DOI:** 10.1038/s41467-021-26529-9

**Published:** 2021-11-02

**Authors:** Francisco McGee, Sandro Hauri, Quentin Novinger, Slobodan Vucetic, Ronald M. Levy, Vincenzo Carnevale, Allan Haldane

**Affiliations:** 1 Center for Biophysics and Computational Biology, Temple University, Philadelphia, 19122 USA; 2 Institute for Computational Molecular Science, Temple University, Philadelphia, 19122 USA; 3 Department of Biology, Temple University, Philadelphia, 19122 USA; 4 Center for Hybrid Intelligence, Temple University, Philadelphia, 19122 USA; 5 Department of Computer & Information Sciences, Temple University, Philadelphia, 19122 USA; 6 Department of Physics, Temple University, Philadelphia, 19122 USA; 7 Department of Chemistry, Temple University, Philadelphia, 19122 USA

**Keywords:** Computational models, Machine learning, Statistical physics

## Abstract

Potts models and variational autoencoders (VAEs) have recently gained popularity as generative protein sequence models (GPSMs) to explore fitness landscapes and predict mutation effects. Despite encouraging results, current model evaluation metrics leave unclear whether GPSMs faithfully reproduce the complex multi-residue mutational patterns observed in natural sequences due to epistasis. Here, we develop a set of sequence statistics to assess the “generative capacity” of three current GPSMs: the pairwise Potts Hamiltonian, the VAE, and the site-independent model. We show that the Potts model’s generative capacity is largest, as the higher-order mutational statistics generated by the model agree with those observed for natural sequences, while the VAE’s lies between the Potts and site-independent models. Importantly, our work provides a new framework for evaluating and interpreting GPSM accuracy which emphasizes the role of higher-order covariation and epistasis, with broader implications for probabilistic sequence models in general.

## Abstract (web summary)

Generative models have become increasingly popular in protein design, yet rigorous metrics that allow the comparison of these models are lacking. Here, the authors propose a set of such metrics and use them to compare three popular models.

## Introduction

Recent progress in decoding the patterns of mutations in protein multiple sequence alignments (MSAs) has highlighted the importance of mutational covariation in determining protein function, conformations, and evolution, and has found practical applications in protein design, drug design, drug resistance prediction, and classification^1–3^. These developments were sparked by the recognition that the pairwise covariation of mutations observed in large MSAs of evolutionarily diverged sequences belonging to a common protein family can be used to fit maximum entropy “Potts” statistical models^4–6^. These models contain pairwise statistical interaction parameters reflecting epistasis^7^ between pairs of positions, such that the character at one position affects the character biases at the other position. Such models have been shown to accurately predict physical contacts in protein structure^6,8–10^, and have been used to significantly improve the prediction of the fitness effect of mutations to a sequence compared to site-independent sequence variation models which do not account for covariation^11,12^. They are “generative” in the sense that they define the probability, *p*(*S*), that a protein sequence *S* results from the evolutionary process. Intriguingly, the probability distribution *p*(*S*) can be used to sample unobserved, and yet viable, artificial sequences^13–17^. In practice, the model distribution *p*(*S*) depends on parameters that are found by maximizing a suitably defined likelihood function on observations provided by the MSA of a target protein family. As long as the model is well specified and generalizes from the training MSA, it can then be used to generate new sequences, and thus a new MSA whose statistics should match those of the original target protein family. We refer to probabilistic models that create new protein sequences in this way as generative protein sequence models (GPSMs).

The fact that Potts maximum entropy models are limited to pairwise epistatic interaction terms and have a simple functional form for *p*(*S*) raises the possibility that their functional form is not flexible enough to describe the data, i.e., that the model is not well specified. A model with only pairwise interaction terms can predict complex patterns of covariation involving three or more positions through chains of pairwise interactions, but it cannot model certain triplet and higher patterns of covariation that require a model with more than pairwise interaction terms^18^. For example, a Potts model cannot predict patterns described by an XOR or boolean parity function in which the *n*th residue is determined by whether an odd number of the *n* − 1 previous residues have a certain value (see Supplementary Information, Supplementary Note 6). While some evidence has suggested that in the case of protein sequence data the pairwise model is sufficient and necessary to model sequence variation^19–21^, some of this evidence is based on averaged properties, and there appears to be some weak evidence for the possibility of rare “higher-order epistasis” affecting protein evolution^7,22–24^, by which we mean the possibility that subsequence frequencies of three or more positions cannot be reproduced by a model with only pairwise interactions. Fitting maximum entropy models with all triplet interactions is not feasible without significant algorithmic innovation, since for a protein of length 100 it would require approximately 1*B* parameters and enormous MSA datasets to overcome finite sampling error (see Supplementary Notes 4 and 6). However, recent developments in powerful machine learning techniques applied to images, language, and other data have shown how complex distributions *p*(*S*) can be fit with models using more manageable parameter set sizes. Building on the demonstrated power of incorporating pairwise epistasis into protein sequence models, this has motivated investigation of machine learning strategies for generative modeling of protein sequence variation which can go beyond pairwise interactions, including Restricted Boltzmann Machines (RBMs)^3^, variational autoencoders (VAEs)^25–28^, Generative Adversarial Networks (GANs)^29^, transformers^30–35^, and others^36–38^.

One model in particular, the VAE^39,40^, has been cited as being well suited for modeling protein sequence covariation, with the potential to detect higher order epistasis^25,26^. The VAE also potentially gives insight into the topology of protein sequence space through examination of the “latent” (hidden) parameters of the model, which have been suggested to be related to protein sequence phylogenetic relationships^25–27,34^. One implementation of a VAE-GPSM, “DeepSequence”, found that the VAE model was better able to predict experimental measurements of the effect of mutations reported in deep mutational scans than a pairwise Potts model, which was attributed to the VAE’s ability to model higher-order epistasis^11,25^. However, it has also been suggested by others that the improvement reported for DeepSequence could be attributed to the use of biologically motivated priors and engineering efforts, rather than because it truly captured higher-order epistasis^26^. Furthermore, while VAE-GPSMs are generative and aim to capture the protein sequence distribution *p*(*S*), to our knowledge none of these studies have systematically tested what we will call the “generative capacity”^41,42^ of the VAE model, meaning the ability of the model to generate new sequences drawn from the model distribution *p*(*S*), which are statistically indistinguishable from those of a given “target” protein family. Testing the generative capacity, specifically higher-order covariation, of a GPSM is a fundamental check of whether the model is well specified and generalizes from the training set, two prerequisites to capturing higher-order epistasis.

To fill this gap, we aim to develop systematic measures of GPSM accuracy or generative capacity, and to use these measures to compare the generative capacity of different GPSMs. Motivated by questions related to the importance of pairwise and higher-order epistasis in modeling protein datasets, we focus on the forms of model mis-specification related to higher-order patterns of covariation. This has not been well explored for sequence models generally, yet may play an important role in many datasets other than protein sequence MSAs. We perform a series of numerical experiments using GPSMs of current interest, including a pairwise Potts Hamiltonian model with only pairwise interaction terms (Mi3)^43^, two state-of-the-art implementations of a variational autoencoder, and a site-independent model which does not model covariation (Indep). One VAE implementation is a standard VAE (sVAE) using an architecture nearly identical to “EVOVAE”^27^, which closely follows the VAE inference method as it was originally presented^39,40^. The second VAE implementation is DeepSequence, mentioned above, which uses a deeper neural network and a more sophisticated optimizer^25^. All of these GPSMs are applicable to sequence datasets besides protein MSAs. We note that not all protein sequence models are strictly GPSMs in the sense we define below, meaning models with a well-defined probability distribution *p*(*S*) describing sequences in a single protein family. For instance, the transformer model of ref. ^31^ is not strictly a GPSM of the kind we study here (see Supplementary Note 13).

Which forms of model mis-specification can be detected by different GPSM metrics? Current studies of GPSM accuracy often test the correspondence of GPSM predictions with external experimental measurements of proteins. For instance, it is common to compare model predictions of *p*(*S*) for a sequence *S* to experimental fitness values from deep mutational scans^25,27^, or test that generated artificial sequences appear to fold into realistic structures according to in silico folding energy^32^. Although such predictions have important applications, they are indirect measures of GPSM accuracy, and are subject to experimental error or computational chemistry assumptions and precision limits. Further, protein function and fitness do not depend exclusively on the thermodynamic stability of static native structures, but also on the protein’s conformational dynamics^44–49^. This could mean that despite generating sequences with realistic in silico folding energy, a GPSM may still not be capturing crucial higher-order epistatic effects. Such tests are also specific to protein sequence datasets and are not necessarily applicable to other sequence data.

A more direct test of GPSM generative capacity would compare the statistical properties of generated sequences to those of the dataset MSA. A number of such measures have been used to evaluate GPSMs. Here, we test three standard metrics, which are the pairwise covariance correlations^2,17,21,50^, Hamming distance distributions^2,21,51,52^, and statistical energy correlations^21,25,38^. However, as explained below, these do not directly test the model’s ability to reproduce higher-order covariation since they measure properties of only pairs of residues or of whole sequences. In the field of Natural Language Processing other standard metrics have been developed, such as *n*-gram perplexity^53,54^ and BLEU^55^, most notably for transformers^31,32,56,57^. For instance the unigram perplexity is the exponentiated product of the likelihoods of single tokens (residues, in protein sequences) over all sites in generated sequences, according to reference dataset token frequencies, thus testing whether the model generates likely tokens. This is sometimes extended to bigrams and *n*-grams for small *n*, and *k*-skip-*n*-grams^55,58^, to test that the generated groups of nearby tokens (or residues) are likely. However, in protein datasets the informative context of a residue includes long-range relationships with other residues corresponding to tertiary structure, which are not probed by short-range *n*-gram-based metrics. Additionally, certain sequence motifs must appear at specific positions in protein MSAs, while *n*-gram metrics are usually position-independent. For these reasons, here we develop and test a fourth and novel metric designed to systematically probe a GPSM’s ability to reproduce the complex patterns of mutational covariation over many and distant positions, which we call *r*_20_^21^.

Our key results are that, first, the *r*_20_ metric is a more sensitive measure of GPSM generative capacity than the other standard metrics, representing a powerful new method to discriminate between GPSMs with respect to their ability to model higher-order covariation up to the 10th order. Using this metric together with the three standard metrics, we compare the performance of the Mi3 model, both VAE models, and the Indep model. We find that for all metrics tested the two VAE models perform similarly to each other, and while they model some covariation and are more accurate than the Indep model which cannot model covariation by definition, they are unable to reproduce MSA statistics as accurately as Mi3. In other words, according to these metrics, the “deep” networks used in the VAE architecture do not appear to model protein sequence datasets as well as the Mi3 model, which has a simpler functional form involving only pairwise interaction terms. By quantifying and comparing GPSM performance in our innovative epistasis-oriented approach, we hope to better understand the challenges and limitations inherent to generative modeling of natural protein sequence datasets, better gauge the state of the art, and provide insight for future efforts in terms of minimizing the confounding effects of data limitations in generative protein sequence modeling and sequence models more generally.

## Results

### Target probability distributions

Our goal is to set baseline expectations for the generative capacity of GPSMs when fit to synthetic or natural protein sequence data of varying training MSA sizes. Generative models of protein MSAs define a distribution *p*_θ_(*S*) for the probability of a sequence *S* to appear in an MSA dataset given model parameters *θ*. The model parameters are fit by either exact or approximate maximum likelihood inference of the likelihood ${{{{{{{\mathcal{L}}}}}}}}={\prod }_{S\in {{\mbox{MSA}}}}{p}_{\theta }(S)$ over a training MSA, using regularization techniques to prevent overfitting. The sequences in the training MSA are assumed to be independent and identically distributed (i.i.d.) samples from a “target” probability distribution *p*^0^(*S*), which is generally unknown^39^. For a model with high generative capacity, *p*_θ_(*S*) will closely approximate *p*^0^(*S*)^26^. Each GPSM we test has a different functional form of *p*_θ_(*S*).

It is not possible to measure the similarity of *p*_θ_(*S*) and *p*^0^(*S*) directly because of the high dimensionality of sequence space, since the number of sequence probabilities to compare is equal to *q*^L^, where *L* is the sequence length (typically ~300) and *q* is the alphabet size (~21). Instead, we measure how derived statistics computed from evaluation MSAs generated by each GPSM match those of target MSAs drawn from the target probability distribution. Three of these are standard metrics: the pairwise Hamming distance distribution, the pairwise covariance scores, and the GPSM’s ability to predict *p*^0^(*S*) for individual sequences, also called statistical energy and abbreviated *E*(*S*) (see “Methods” section). We introduce a fourth metric, the averaged higher order marginal accuracy, *r*_20_, which we argue most directly tests a GPSM’s ability to model higher-order covariation.

### GPSM error and experiments

In our experiments, we probe and isolate three distinct forms of error which may cause *p*_θ_(*S*) to deviate from *p*^0^(*S*) (see Table 1). The first is “specification error”^59^, which occurs when the functional form of *p*_θ_(*S*) of a model is not flexible enough to accurately model the target probability distribution *p*^0^(*S*) for any choice of parameters. A key motivation for choosing a VAE over a Potts model is its potentially lower specification error when higher-order epistasis is present^25^. Indeed, Potts models are limited to pairwise interaction terms of a particular functional form, while VAEs presumably are not. The second form of error is “out-of-sample error”^60^, caused by a paucity of training samples, and is the consequence of overfitting^61^. Even a well-specified model could fail to generalize when fit to a small training dataset, and may mis-predict *p*^0^(*S*) for test sequences, so it follows that increasing training MSA size reduces out-of-sample error. Beyond specification and out-of-sample error, which each reflect an aspect of GPSM generalization error, there can be “estimation error”^62^ in our MSA test statistics due to the finite MSA sizes we use to estimate their values, which sets an upper bound on how well these statistics can match their target values, depending on the metric. Finally, other errors may arise due to implementation limitations of the inference methods, for instance due to finite precision arithmetic or to finite sample effects when Monte Carlo methods are used.

**Table 1:** Glossary of error types, and the MSA datasets we use to evaluate these errors.

Error name	Error definition
Specification error	Occurs when the functional form *p*_*θ*_(*S*) of a GPSM is not flexible enough to accurately model the target probability distribution *p*^0^(*S*) for any choice of parameters *θ*
Out-of-sample error	Occurs when a GPSM fit to a finite training dataset fails to correctly model unseen data, and is a consequence of overfitting
Estimation error	Occurs due to statistical error in the MSA evaluation metrics when computed from finite evaluation and target MSAs
MSA name	MSA description
Training MSA	Drawn from *p*^0^(*S*), used to train or parameterize a GPSM
Target MSA	Drawn from *p*^0^(*S*) separately from the training MSA, used as a validation dataset
Evaluation MSA	Drawn from *p*_*θ*_(*S*) for a parameterized GPSM, used to compare to the target MSA

**Fig. 1:**
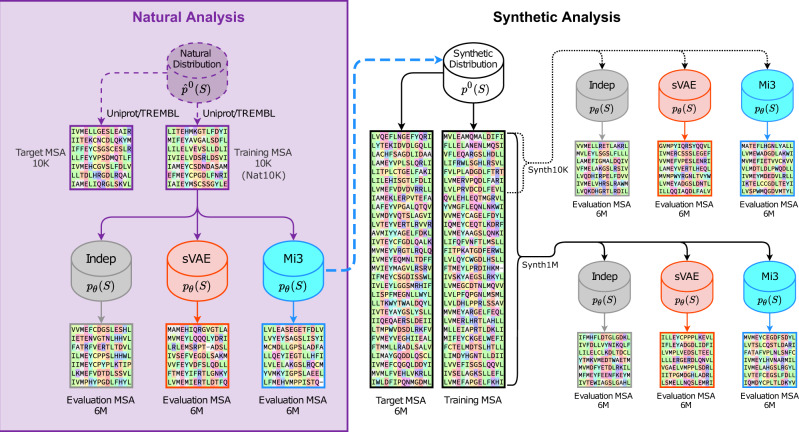
Workflow of natural and synthetic analyses. We perform two analyses, one Natural (left, purple) and the other Synthetic (right, black). In both analyses, we train three types of GPSMs on protein MSAs, which are Indep (gray), VAEs (orange), and Mi3 (blue). Because the performance of both VAEs tested (sVAE and DeepSequence) perform equivalently, only sVAE is included in this figure for simplicity. To compare performance across GPSMs, we generate an evaluation MSA of 6M synthetic sequences from each GPSM after training, and test generative capacity by comparing the evaluation MSA to the target MSA. The target MSA is non-overlapping with the training MSA, but was sampled from the same target probability distribution. Left: In the Natural Analysis, we train the GPSMs on 10*K* protein sequences (Nat10K) taken from Uniprot/TREMBL. The natural target probability distribution (purple) is unknown, so we demarcate its container with a dashed line and define it as ${\hat{p}}^{0}(S)$ to emphasize this unique property, in contrast to all other probability distributions which we know exactly. The dashed lines leaving the Natural Distribution indicate that the non-overlapping 10K training and target MSAs are the result of aligning and phylogenetic filtering of sequences obtained from Uniprot/TREMBL. In the Natural Analysis, we are data-limited to ~10K samples for the non-overlapping training and target MSAs. Right: In the Synthetic Analysis, we use a known Synthetic Distribution, *p*^0^(*S*), from which we can generate arbitrarily large MSAs. The dashed blue line pointing to the Synthetic Distribution is to emphasize that it is precisely the Mi3 GPSM distribution constructed in the Natural Analysis. In one experiment (top right), we train the GPSMs on only 10K synthetic sequences (Synth10K) to mimic the Natural Analysis. However, we perform generative capacity measurements on a target MSAs of 6M sequences, removing estimation error from the measurements. In the second experiment (bottom right), we train the GPSMs on 1M training sequences (Synth1M), which include the 10K from the first experiment. With these large training and target MSAs, both out-of-sample and estimation error are removed from the generative capacity measurements, leaving only specification error, if any.

To disentangle these three different forms of error, we divide our tests into two analyses: one natural, in which we train the GPSMs on a representative natural protein family MSA (Nat10K), the kinase super family, sequestered from Uniprot/TrEMBL^63^ (Fig. 1, left); and one synthetic, in which we train the GPSMs on synthetic MSAs of varying sizes (Synth10K, Synth1M) generated from a known distribution (Fig. 1, right). To demonstrate the generality and robustness of our findings, we repeat some of the synthetic experiments on three other protein families for which large amounts of sequence data are available (RRM, response regulator, and ABC transporter, see “Methods” section), with comparable results to kinase (see Supplementary Note 11).

The natural analysis (Fig. 1, left) examines GPSM performance on natural sequence data, which could potentially contain higher-order correlations that require a Hamiltonian model with triplet or higher-order interaction terms to capture. On this dataset, the VAEs could potentially outperform Mi3, depending on the importance of higher-order epistatic terms, if present^24,25^. However, unlike in the synthetic analysis described next, here we do not know a priori the natural distribution and, most importantly, we have only limited datasets for both training and measurement, which is roughly ~10K sequences for the largest protein families in Pfam^64^ (see Supplementary Note 12). To distinguish the natural distribution as unknown, we denote it as ${\hat{p}}^{0}(S)$ (Fig. 1, left). In the natural analysis, the training and target MSAs each contain ~10K non-overlapping kinase sequences from the Uniprot/TREMBL database after phylogenetic filtering at 50% sequence identity. After filtering, we consider the sequences to be i.i.d. samples of the evolutionary process, having the unknown equilibrium distribution ${\hat{p}}^{0}(S)$.

The synthetic analysis (Fig. 1, right) allows us to isolate specification error by largely eliminating both out-of-sample and estimation error, because here the target probability distribution *p*^0^(*S*) is known exactly and we can generate arbitrarily large non-overlapping training, target, and evaluation datasets. We choose the synthetic target probability distribution *p*^0^(*S*) to be exactly the Potts model distribution we inferred based on natural protein kinase sequence data using Mi3 in our natural analysis (Fig. 1) (see “Methods” section)^43^. The sequences generated from this synthetic target probability distribution should have statistical properties similar to real, or “natural”, protein family MSAs, albeit constrained by the fact that the Hamiltonian model used to generate the synthetic dataset is limited to pairwise epistatic interaction terms only^6,21,65^. Whereas Indep and the VAEs may still fail to model this known probability distribution in the synthetic analysis, our expectation is that Mi3 will be unaffected by specification error in the synthetic tests, since the target MSA is sampled from the same probability distribution used to carry out the inference. We also perform an alternate synthetic test which does not favor Mi3 in this way, in which the target probability distribution is instead specified by sVAE, finding that both Mi3 and sVAE are able to fit this target sVAE distribution accurately, and Mi3 still outperforms sVAE (see Supplementary Note 5).

The synthetic analysis also allows us to quantify out-of-sample error by modulating the training MSA size, and we test two synthetic training MSA sizes: (i) 1M sequences (Synth1M), to minimize overfitting effects and consequently out-of-sample error, thereby isolating GPSM specification error in this experiment; and (ii) 10K sequences (Synth10K), to illustrate the expected GPSM performance on typical datasets, as most protein families in Pfam have less than 10K effective sequences^64^. 1M training sequences appears to be sufficient to largely eliminate out-of-sample error, even though our Mi3 and VAE models have more than 1M parameters (see Supplementary Note 4). We have previously reported how out-of-sample error for GPSMs is not simply a function of the number of sequences relative to the number of model parameters, but also depends on the degree of conservation, in ref. ^65^, and based on that analysis it is not unexpected that only 1M sequences are sufficient to make out-of-sample error (overfitting) negligible, even if the number of model parameters is much larger.

Our overall experimental procedures are outlined in Fig. 1. Our training datasets are either a natural protein sequence dataset obtained from Uniprot/TREMBL, or a synthetic dataset generated by a Mi3 model on natural data (or, as tested in Supplementary Note 5, an sVAE model). In the main text we show results only for kinase, but we present comparable results for RRM, response regulator, and ABC transporter in Supplementary Note 11. The experiments begin by fitting the GPSMs to the training datasets, followed by generation of evaluation MSAs from each GPSM. Finally, using our suite of four generative capacity metrics, we compare statistics of the evaluation MSAs to those of “target” MSAs, which contain sequences drawn from the target probability distributions that are non-overlapping with the training set, and therefore represent our expectation.

### Pairwise covariance correlations

We first examine the pairwise covariance scores for pairs of amino acid residues of an MSA defined as ${C}_{\alpha \beta }^{ij}={f}_{\alpha \beta }^{ij}-{f}_{\alpha }^{i}{f}_{\beta }^{j}$. Here, ${f}_{\alpha \beta }^{{{{{{\rm{ij}}}}}}}$ are the MSA bivariate marginals, meaning the frequency of amino acid combination *α*, *β* at positions *i*, *j* in the MSA. ${f}_{\alpha }^{{{{{{\rm{i}}}}}}}$ and ${f}_{\beta }^{{{{{{\rm{j}}}}}}}$ are the univariate marginals, or individual amino acid frequencies at positions *i* and *j*. Each covariance term measures the difference between the joint frequency for pairs of amino acids and the product of the single-site residue frequencies, i.e., the expected counts in the hypothesis of statistical independence. If ${C}_{\alpha \beta }^{{{{{{\rm{ij}}}}}}}$ equal 0 for all *αβ* then the positions *i*, *j* do not covary. Coevolving amino acids are an important aspect of sequence variation in protein MSAs, and a GPSM’s ability to reproduce the pairwise covariance scores of the training dataset has been used in the past as a fundamental, non-trivial measure of the GPSM’s ability to model protein sequence covariation^2,17,21,50^.

**Fig. 2:**
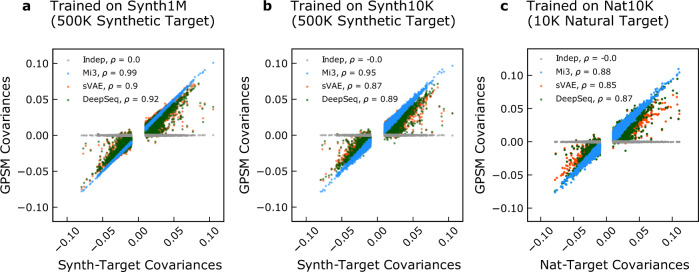
Ability of GPSMs to capture pairwise covariances. Mi3 (blue), DeepSequence (green), sVAE (orange), and Indep (gray) MSA statistics are compared to the target probability distribution values. GPSMs were trained on 1M synthetic (Synth1M, **a**), 10K synthetic (Synth10K, **b**), or 10K natural (Nat10K, **c**) kinase sequences from the corresponding target probability distribution. Covariances ${C}_{\alpha \beta }^{{{{{{\rm{ij}}}}}}}$ were computed from MSAs of 500K sequences generated by each GPSM (*y*-axis) vs. target covariances (*x*-axis) computed from MSAs of 500K sequence from the target probability distribution (**a**, **b**) or from 10K natural target sequences (**c**), with Pearson correlation *ρ* shown for each comparison. Generative capacity only slightly decreases as training sample size is reduced for all GPSMs from panels **a** to **b**, indicating insensitivity to training sample size. Covariances around zero are omitted to simplify the plot, but are included in the Pearson correlation.

For each GPSM, we compare pairwise covariance scores for all pairs of positions and residues ${\hat{C}}_{\alpha \beta }^{{{{{{\rm{ij}}}}}}}$ in their respective evaluation MSA to the corresponding target pair ${C}_{\alpha \beta }^{{{{{{\rm{ij}}}}}}}$ in the target MSA using the Pearson correlation coefficient $\rho (\{{C}_{\alpha \beta }^{{{{{{\rm{ij}}}}}}}\},\{{\hat{C}}_{\alpha \beta }^{{{{{{\rm{ij}}}}}}}\})$ (Fig. 2). In the synthetic tests we evaluate this statistic using 500K sequences for both the target and evaluation MSAs, while for the natural test we compare 500K evaluation sequences to the available 10K target sequences. Indep cannot reproduce covariances by definition, so *ρ* is zero in all tests, as expected.

Mi3 accurately reproduces the target covariance scores in all tests (*ρ* = 0.99 in Fig. 2a, *ρ* = 0.95 in Fig. 2b, and *ρ* = 0.88 in Fig. 2c, respectively). The high accuracy of Mi3 is expected for this measurement, because Mi3’s parameters are optimized to exactly reproduce the joint frequencies of pairs of amino acids from the target MSA. In the natural analysis, the somewhat lower value for Mi3 of *ρ* = 0.88 is accounted for entirely by the increased estimation error in that test, as only 10K target sequences are available for evaluation. The expected *ρ* due only to estimation error is *ρ* ~ 0.87, which is computed by comparing the natural 10K target sequences to the natural 10K training sequences using this metric.

The VAEs’ inference does not include the same constraint to reproduce the joint frequencies of pairs, but we find that even when trained on the larger (1M) dataset of synthetic sequences to minimize out-of-sample error, the covariances computed from the VAEs’ evaluation MSAs are consistently smaller in magnitude than those of the target MSA, and have smaller correlation with the target than Mi3 (*ρ* = 0.9 for sVAE and *ρ* = 0.92 for DeepSequence in Fig. 2a). For the VAEs, this amount of error in *ρ* can primarily be attributed to specification error, since training GPSMs on 1M sequences largely eliminates out-of-sample error, and the large evaluation MSAs make estimation error negligible. The VAEs’ covariances are further scaled down slightly in magnitude when fit to the synthetic 10K dataset (*ρ* = 0.87 for sVAE and *ρ* = 0.89 for DeepSequence in Fig. 2b).

These results are consistent between the synthetic and natural analyses for all GPSMs, showing the behavior is not due to artificial properties of our synthetic target model. These results confirm that VAEs can model pairwise epistasis in protein sequence datasets, since they generate pairwise mutational covariances that are correlated with the target values, even in the absence of explicit constraints for reproducing these statistics. However, they scale down the strength of pairwise covariances in both the synthetic and natural analyses and the correlation with the target is lower than 1. Mi3, in contrast, is constrained by design to fit the pairwise covariance scores and does so nearly perfectly.

### Higher order marginal statistics

**Fig. 3:**
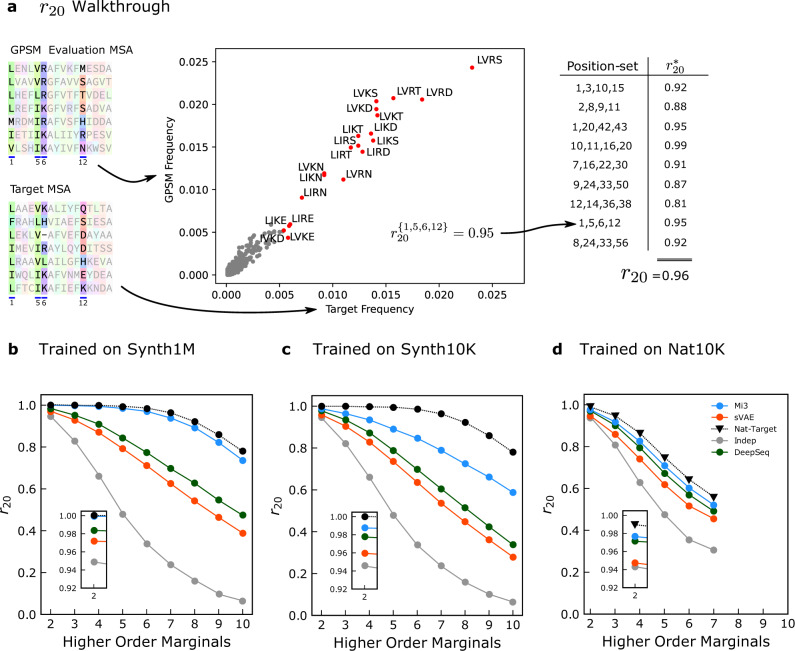
Ability of GPSMs to capture higher-order covariation (*r*_20_). **a** Diagram of *r*_20_ calculation for *n* = 4. Left: For a particular set of positions, {1, 5, 6, 12} in this case, the subsequence frequencies for all words at these positions is calculated for the target MSA and the evaluation MSA. Middle: The word frequencies for the two MSAs are compared, and the top-20 most frequent words according to the target MSA (red points, labeled by word) are used to compute the Pearson-*r* value between the two sets of values. Right: This procedure is repeated for many different position-sets. The final *r*_20_ score is the average of the *r* across position-sets. Bottom row: Pearson *r*_20_ score (*y*-axis) as a function of Higher-Order-Marginal (HOM) word length (*x*-axis), illustrating how well each GPSM predict HOMs from the target probability distribution. GPSMs were trained on 1M synthetic (Synth1M, **b**), 10K synthetic (Synth10K, **c**), or 10K natural (Nat10K, **d**) sequences from the corresponding target probability distribution. *r*_20_ scores for each GPSM are computed using GPSM-generated evaluation MSAs of 6M sequences compared to target MSAs of 6M sequences for the synthetic tests (**b**, **c**) or to a target MSA of 10K natural sequences for the natural test (**d**) due to limited natural sequence data. The black dotted line denotes the upper bound for *r*_20_ due to estimation error given the evaluation MSA size of 6M for the synthetic analysis and 10K for the natural analysis. This reflects the expected value if the GPSM had modeled *p*^0^(*S*) exactly given these MSA sizes. It must be approximated for the natural test as signified using triangle markers (see text for detail). For panel **d**, only lengths 2 through 7 are plotted, as the small dataset size limits HOM estimation. Insets emphasize pairwise *r*_20_. Comparing panels **c** and **d**, it appears that the generative capacity of Mi3 and the VAEs are sensitive to decreasing synthetic training sample size for *r*_20_, whereas Indep is insensitive.

A more stringent test of GPSM generative capacity is to measure the model’s ability to reproduce sequence covariation involving more than two positions, or higher-order covariation. We characterize these higher-order mutational patterns in the target MSA and GPSM-generated evaluation MSAs by computing the frequency of non-contiguous amino acid “words” of length *n*, or higher-order marginals (HOMs) corresponding to subsequences, and compare their frequency in each MSA to corresponding values in the target MSAs, as illustrated in Fig. 3a. For increasing values of *n* the number of possible words increases rapidly, requiring increasingly large evaluation MSAs to accurately estimate the frequency of individual words. For this reason, we limit word length to *n* ≤ 10 and only compute a limited subset of all possible position sets for each *n*. For each *n* we randomly choose 3K position sets, compute the frequencies of the top twenty most frequent words for each corresponding position set in the target and evaluation MSAs, as these are well sampled, and for each position-set compute the Pearson correlation *r* between these top twenty frequencies. We then average the correlation values for each *n* over all position-sets. We call this metric *r*_20_^21^ reflecting the fact that it is computed from the Pearson correlation *r* for the top 20 most frequent words. Unlike our other metrics, the estimation error for *r*_20_ is non-negligible because of the extremely large MSAs required to compute word frequencies, particularly for high *n* > 5 (see Supplementary Note 8). We can predict the estimation error caused by finite sampling in the evaluation MSAs by computing the *r*_20_ scores between two non-overlapping MSAs generated by the synthetic target model, which are of the same size as our evaluation MSAs. Similar computations of 3rd and sometimes 4th order statistics have previously been reported^50,66–69^, but the *r*_20_ score systematizes these statistics to higher orders.

In Fig. 3, we plot the HOM *r*_20_ for varying word length *n*. The expected estimation error (black line) represents an upper bound for *r*_20_ giving the highest measurable *r*_20_ given the finite target MSA size of 6M for the synthetic analysis and 10K for the natural analysis. This corresponds to the case the GPSM had perfectly modeled *p*^0^(*S*), with no specification or out-of-sample error, leaving only estimation error caused by the use of finite evaluation MSA sizes.

In the synthetic analysis, we compute this upper-bound by generating two MSAs of size 6M from *p*^0^(*S*) and computing the *r*_20_ score between them. The *r*_20_ for Mi3 fit to 1M synthetic training sequences is very close to this validation upper-bound for all *n*, suggesting it has accurately fit the synthetic target probability distribution, and its specification error is close to 0 (Fig. 3b). This is expected since the synthetic target model in this test is a Potts model. With 10K synthetic training sequences, Mi3 *r*_20_ scores are lower than the 1M result for all *n* (Fig. 3c), which illustrates that Mi3 is affected by out-of-sample error for typical dataset sizes^65^.

Indep has much lower *r*_20_ scores than Mi3, as expected, since it does not model pairwise epistasis by design and is a poor model of the word frequencies. Indep’s *r*_20_ scores are similar across all experiments, suggesting that it is not strongly affected by out-of-sample error. This is expected because its parameters are optimized for reproducing single-site frequency statistics only, which can be accurately estimated even from small training MSAs^65^.

The *r*_20_ scores for both sVAE and DeepSequence are approximately halfway between Mi3 and Indep, and generally well below Mi3 at higher orders. The scores for DeepSequence are slightly higher than sVAE’s, but both VAEs remain close to each other for all training datasets and *n*. In the 1M training MSA experiment, the VAEs’ *r*_20_ decreases to ~0.4 and ~0.5 at *n* = 10 for sVAE and DeepSequence respectively, reflecting high specification error at higher orders (Fig. 3b). With 10K synthetic training sequences, the VAEs’ *r*_20_ decreases further for all *n* due to the addition of out-of-sample error (Fig. 3c).

In the natural analysis, because we do not have knowledge of ${\hat{p}}^{0}(S)$ or access to large MSAs generated from ${\hat{p}}^{0}(S)$, the *r*_20_ metric is subject to much larger estimation error due to the small 10K target MSA, and so we cannot construct the estimation upper-limit in the same way as was done in the synthetic analysis (black line, Fig. 3d). Despite this, we observe again that the Mi3 model performs best, and the VAE models are intermediate between Mi3 and Indep, although the difference between the models is smaller than in the synthetic test as the metric is obscured by the high estimation error. Since we cannot generate new sequences from ${\hat{p}}^{0}(S)$ in the natural analysis, we can only approximate the upper-bound due to the estimation error, which we do by using the Mi3 model distribution *p*_θ_(*S*) as an approximation to ${\hat{p}}^{0}(S)$, and generate two 10K and 6M sequence MSAs from *p*_θ_(*S*) and compare them using *r*_20_. This provides only an estimate of the expected *r*_20_ if the GPSM had modeled ${\hat{p}}^{0}(S)$ exactly, since ${\hat{p}}^{0}(S)$ will differ from *p*_θ_(*S*) when the training MSA is only 10K sequences. Nevertheless, in Fig. 3d, we observe that the Mi3 *r*_20_ score is very close to this estimate, which is consistent with the hypothesis that the Potts model is well specified to the natural protein sequence dataset, and the small difference can arise due to the approximation in the upper-bound.

These *r*_20_ results reinforce our findings using the pairwise covariations ${C}_{\alpha \beta }^{{{{{{\rm{ij}}}}}}}$, which gave a preliminary indication that VAEs capture epistasis but mispredict its strength, and extends these initial indications into higher orders. Unlike Mi3, the VAEs show specification error even when fit to large datasets from a model which only contains pairwise epistatic interaction terms (Fig. 3b). Because higher-order covariation statistics are constrained by the pairwise statistics, and the VAEs mispredict the pairwise statistics, we expect that the VAEs will exhibit specification error for higher-order epistasis. We also observe that when trying to gauge GPSM generative capacity at higher orders of covariation, the standard pairwise statistics alone can be misleading. The relative magnitudes of *r*_20_ between models at *n* = 2 are different at higher *n*, and the performance decrease as *n* increases is more severe for the VAEs than Mi3 (Fig. 3, bottom row).

The sensitivity of the *r*_20_ metric for detecting model mis-specification involving higher-order covariation raises the question of which forms of mis-specification this metric can detect, and whether variants of this metric could be even more sensitive. We discuss some alternatives here as well as the advantages of the *r*_20_ formulation of the metric in Supplementary Note 7.

One type of mis-specification the *r*_20_ metric potentially fails to detect involves low frequency words below the top 20. However, if epistatic interactions increase the frequencies of low frequency words, of which there are many, this necessarily reduces the frequency of the highest frequency words since the frequencies must sum to 1. In this way *r*_20_ indirectly measures the balance in probabilities between the low frequency words and the individual highest 20 frequency words.

Another potential limitation is that the *r*_20_ metric does not directly test for covariation as measured by ${C}_{\alpha \beta }^{{{{{{\rm{ij}}}}}}}$, as it instead tests whether the GPSM accurately reproduces marginals. To account for this, we test an alternative to *r*_20_ based on the “connected correlations”^70^, which are a generalization of ${C}_{\alpha \beta }^{{{{{{\rm{ij}}}}}}}$ to higher orders, which we call cc − *r*_20_ (see Supplementary Note 7). We compute it similarly to *r*_20_ but replace the top 20 word frequencies by the corresponding connected correlations. These results are qualitatively similar to the *r*_20_ results, and Mi3 most accurately reproduces the connected correlations (see Supplementary Fig. S7). For cc − *r*_20_, the Indep model gives 0 by definition, so that cc − *r*_20_ more specifically tests statistics which cannot be predicted with any accuracy using a site-independent model, unlike *r*_20_. However, cc − *r*_20_ is more severely affected by estimation error than *r*_20_ and cannot be as accurately measured for high *n*, making the *r*_20_ score preferable for higher orders.

Finally, because the *r*_20_ score only tests a limited sample of sets of positions for each *n*, it may potentially fail to sample a particular position-sets which a GPSM fails to model correctly, for example if sparse higher-order interactions are necessary to model the data at those positions. In Supplementary Note 9, we test this by artificially introducing a single triplet interaction term to a Potts model and treat this as the target probability distribution. We then test whether the *r*_20_ metric is able to detect mis-specification of a new pairwise Potts model fit to an MSA generated from this target probability distribution. We find *r*_20_ can indeed detect this mis-specification caused by even a single triplet interaction term. The introduction of an interaction term at one set of positions will affect the marginals at most other positions throughout the MSA because of the chains and networks of interactions necessary to model protein sequence datasets, explaining how *r*_20_ is sensitive to such an interaction.

### Hamming distance distributions

**Fig. 4:**
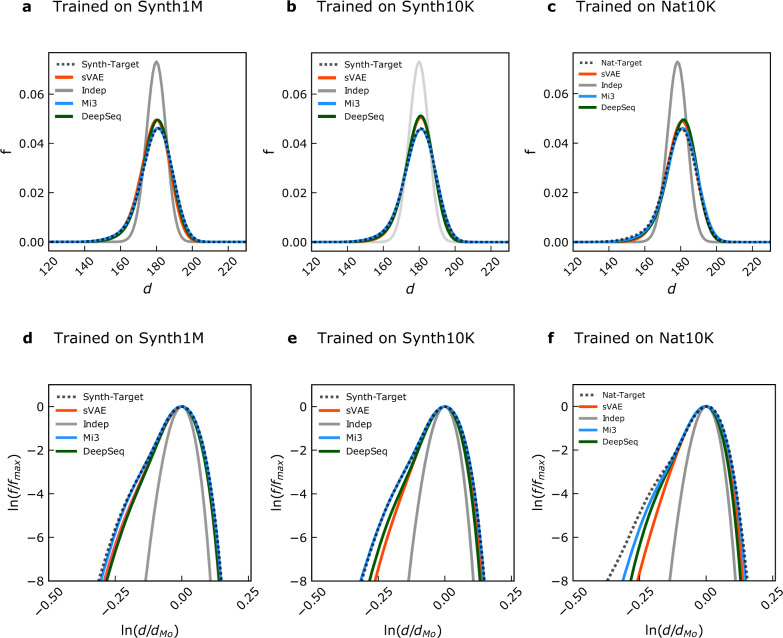
Ability of GPSMs to capture Hamming distance distribution. These plots illustrate whether sequences generated from the GPSMs reproduce the overall sequence diversity of their respective targets. Mi3 (blue), DeepSequence (green), sVAE (orange), and Indep (gray) distributions are compared to target probability distribution (dotted black). GPSMs were trained on 1M synthetic (Synth1M, **a**, **d**), 10K synthetic (Synth10K, **b**, **e**), or 10*K* natural (Nat10K, **c**, **f**) sequences from the corresponding target probability distribution. All Hamming distributions were computed from 50K-sequence MSAs, except for the natural target, which was computed from a 10K-sequence target MSA due to data limitations (see Supplementary Note 12). Top row: Hamming distances *d* (*x*-axis) are shown about the mode, and frequency *f* is normalized as a fraction of total (*y*-axis). Mi3 perfectly matches the target probability distribution from **a** to **c**, whereas DeepSequence and sVAE overlap each other and share a mode with a slightly higher frequency than the target and Mi3. Indep has a slightly lower mode than all the other GPSMs, and a much higher mode frequency. Because generative capacity for all GPSMs is unchanged from **a** to **c**, none of them are sensitive to training or evaluation sample size for this metric. Bottom row: Re-scaled logarithmic Hamming distance distributions better discriminate between GPSMs with respect to generative capacity than the normal Hamming distance distribution. Before being log-scaled, the Hamming distances *d* are normalized by the mode *d*_Mo_ (*x*-axis), and frequencies *f* are normalized by the maximum Hamming distance *f*_max_ (*y*-axis). This transformation highlights minute differences between distributions at low frequencies in the tails of the distributions on the left-hand and right-hand sides. From **d** to **e**, DeepSequence and sVAE appear sensitive to training sample size for this metric at the log-log scale.

We next evaluate the pairwise Hamming distance distribution metric $d(S,S^{\prime} )$. The Hamming distance between two protein sequences is the number of amino acids that are different between them, and we obtain a distribution for an MSA by comparing all pairs of sequences. Because it characterizes the range of sequence diversity in an MSA, recapitulation of the Hamming distance distribution has been used in the past as a measure for GPSM performance^2,21,51,52^. In Fig. 4, we compare the pairwise Hamming distance distribution for each GPSM to that of the target probability distribution, computed with evaluation and target MSAs of 10K sequences each. To quantify the difference between the GPSM and target probability distributions for this metric, we use the total variation distance (TVD)^71^, which equals 1 when the distributions have no overlap and is 0 when they are identical, defined by TVD[*f*, *g*] = 1/2∫∣*f*(*x*) − *g*(*x*)∣d*x*.

All models reproduce the mode Hamming distance of ~179. For Mi3, we report the same TVD = 0.007 when trained on either 1*M* (Fig. 4a) or 10K (Fig. 4b) synthetic sequences, showing negligible specification error, as expected. When trained on 10K natural sequences, Mi3 TVD increases to 0.012 (Fig. 4c), for reasons discussed further below. Indep severely underestimates the probability of both low and high Hamming distances, as observed at the distribution tails, with TVD ~ 0.24 across all experiments. The VAEs perform in between Mi3 and Indep, but much closer to Mi3 than Indep with respect to TVD. Performance differences across all GPSMs for this metric indicate that out-of-sample error has a consistent and detectable, though very small, effect on the fundamental sequence diversity of artificial GPSM-generated MSAs. That Mi3 and the VAEs are highly performant and comparable to each other, but not Indep, corroborates our earlier findings that epistasis is relevant to accurate modeling of protein sequence diversity (Figs. 2 and  3). However, because Indep performs much closer to Mi3 and the VAEs for this metric than on any other, and also because this metric cannot discriminate well between Mi3 and the VAEs, we suspect that reproducing the Hamming distance distribution is a much easier hurdle for GPSMs than is reproducing higher-order covariation. This shortcoming of the standard Hamming distance distribution metric becomes apparent when these results are compared to those of our novel metric, *r*_20_, which does show a significant gap in generative capacity between Mi3 and the VAEs at higher orders (Fig. 3, bottom row).

To emphasize the decay of the tails, we rescale all the distributions by their maxima and re-center them around their modes to give them the same peak, and then plot them on a log-log scale (Fig. 4, bottom row). The relevance of the distributions’ tails lies in their power-law behavior as they approach 0, where the function’s exponent is related to the intrinsic dimension of the dataset and therefore to the number of informative latent factors needed to explain the data^51,52,72^. A well-specified GPSM ought to reproduce this exponent, and therefore the tail’s decay, since it is a topological property intrinsic to the dataset and independent from the particular choice of variables used to describe the probability density^72^. There is a trend of slightly decreasing generative capacity as training samples decrease, which is detectable only here in the log-scaled Hamming distribution (Fig. 4, bottom row). In this modified rendering of the Hamming distance distribution, differences in GPSM generative capacity can be observed at both low (left tail) and high (right tail) sequence diversity. The Mi3 distribution closely overlaps the target probability distribution with both 1M (Fig. 4d) and 10K (Fig. 4e) synthetic training sequences. In the 10K natural experiment, Mi3 deviates noticeably from the target on the left tail (Fig. 4f), which represents less evolutionarily diverged sequences. This could be an artifact of the phylogenetic relationships between sequences present in the natural dataset, which may have been incompletely removed by our phylogenetic filtering step for this dataset (see “Methods” section; Supplementary Notes 11 and 12), or it could be due to estimation error in measuring the target probability distribution, as only 10K target sequences are available to estimate the black line in the natural analysis. As before, Indep performance is consistently low across all experiments. The VAEs’ performance at low sequence diversity (Fig. 4, bottom row, left tails) decreases for smaller training dataset size.

### Statistical energy correlations

A fourth metric we use to evaluate generative capacity is the statistical energy *E*(*S*) of individual sequences in the dataset, which we express using the negative logarithm of the predicted sequence probability *p*(*S*), where $E(S)=-{{{{{{\mathrm{log}}}}}}}\,p(S)$. *E*(*S*) can be computed analytically for Mi3 and Indep, and estimated for VAE models by importance sampling (see “Methods” section, Supplementary Note 2).

**Fig. 5:**
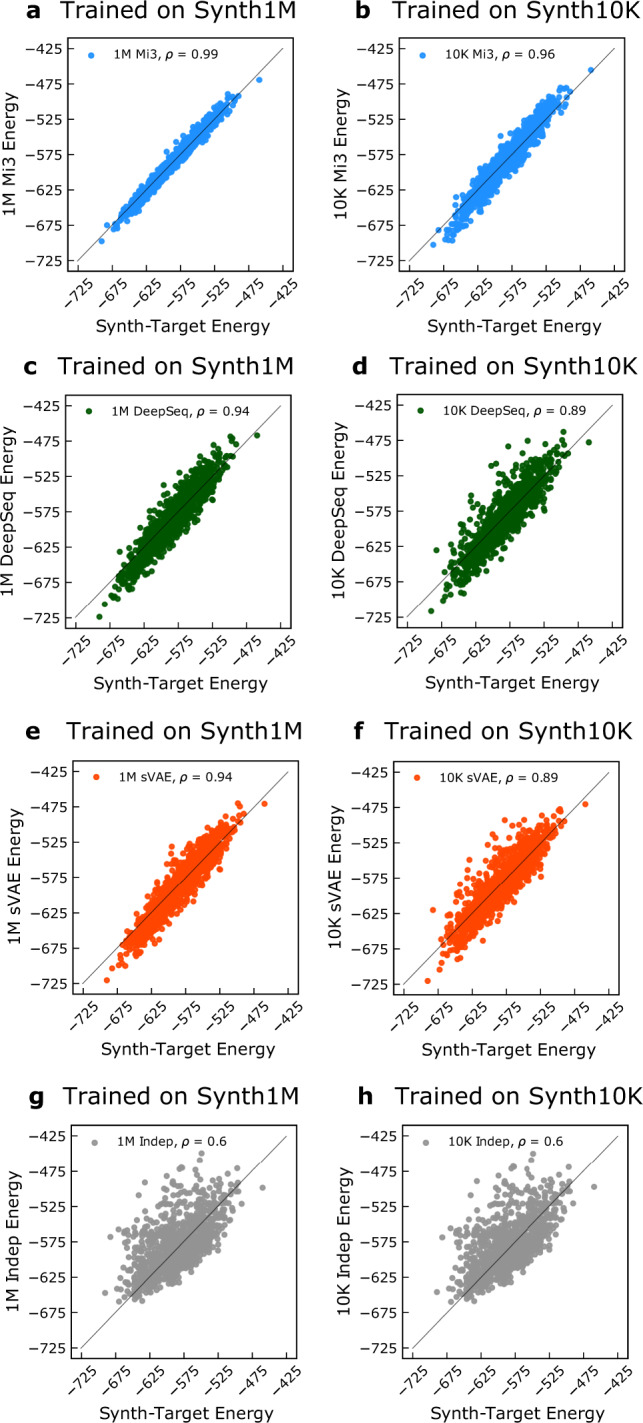
Ability of GPSMs to capture statistical energy, *E*(*S*). Statistical energies *E*(*S*) of 1K synthetic test sequences from the target probability distribution as evaluated by Mi3 (blue, **a**, **b**), DeepSequence (green, **c**, **d**), sVAE (orange, **e**, **f**), Indep (gray, **g**, **h**). Each GPSM was trained on 1M (Synth1M, **a**, **c**, **e**, **g**) or 10K (Synth10K, **b**, **d**, **f**, **h**) sequences from the synthetic target probability distribution. For each scatterplot, Pearson correlation coefficient *ρ* was computed between each GPSM’s statistical energy and that of the synthetic target probability distribution for each sequence. Only Indep is insensitive to decreased training sample size for this metric.

This statistic directly evaluates accuracy of the GPSM distribution values from *p*_θ_(*S*) for a limited number of individual sequences, which has been used to validate GPSMs by comparison to corresponding experimental fitness values^21,25,30,36,38,73^. In Fig. 5, we compare artificial statistical energies from the GPSM distribution *p*_θ_(*S*) to those of the target probability distribution *p*^0^(*S*) for a 1K test MSA generated from *p*^0^(*S*). This measurement cannot be performed for the natural 10K experiment because *p*^0^(*S*) is unknown for the natural data, so we present results only for the 1M and 10K synthetic experiments. We use the 1M (Fig. 5, left column) and 10K (Fig. 5, right column) synthetic training MSA sizes, and quantify GPSM generative capacity for this metric by the Pearson correlation coefficient $\rho (\{E(S)\},\{\hat{E}(S)\})$ between synthetic target energies *E*(*S*) and GPSM energies $\hat{E}(S)$. Mi3 reproduces the synthetic target probability distribution at both training MSA sizes. Because Mi3 should have very low specification error on the synthetic target, as it is well specified by design, the small amount of error must be due to remaining out-of-sample or numerical errors. As expected, Indep poorly reproduces the target values, with *ρ* = 0.6 for both MSA training sizes. The VAEs exhibit slightly larger specification error than Mi3 on the 1M training set with correlation of *ρ* = 0.94, and exhibit further out-of-sample error on the 10K training set with *ρ* = 0.89.

Juxtaposing the *E*(*S*) results to the *r*_20_ results reiterates the striking insight of our work, which is that despite the utility of standard metrics for measuring sequence statistics, they say little about a GPSM’s ability to capture higher-order covariation, and therefore necessarily higher-order epistasis. If considered by itself, the *E*(*S*) metric would indicate that both VAEs have generative capacity close to that of Mi3, and even Indep could be said to have a large amount of generative capacity, despite capturing no covariation by design. But according to *r*_20_, which directly measures the higher-order marginals, the VAEs and Indep are comparable to Mi3 only at the pairwise level. Critically, at higher orders, the performance difference between the VAEs to each other remains relatively unchanged, but is significantly lower than Mi3, with Indep lower still. Additionally, we explore in Supplementary Note 10 how the *E*(*S*) metric has difficulty detecting when a triplet interaction term is necessary to model the sequence data, in a synthetic test. These observations suggest that *E*(*S*), just as with the Hamming distance metric and pairwise covariance correlation, represents an easier, and perhaps different, hurdle for GPSMs than measuring generative capacity as the ability to capture higher-order sequence covariation, as done uniquely by *r*_20_.

## Discussion

In this study, we reveal the steep challenges and limits entailed by current measurements of generative capacity in GPSMs trained on either synthetic or currently available natural sequence datasets. Recent state-of-the-art GPSM studies have benchmarked their models by comparing *p*_θ_(*S*) to experimental fitness values from deep mutational scans^25,27^, or by generating artificial sequences that appear to fold into realistic structures based on in silico folding energy^32^. However, these strategies for model evaluation present their own challenges, for instance because of experimental or modeling error, or because these measurements only probe select components of the protein fitness landscape which do not require epistasis to predict. This could mean that despite generating sequences with realistic in silico folding energy, a GPSM may still not be capturing crucial higher-order epistatic effects. Neither point mutation fitness effects, nor in silico folding energy estimations, are directly related to mutational covariation statistics observed in an MSA in the sense that they do not check if subsets of covarying amino acids in specific positions present in the target MSA are indeed present in the GPSM-generated evaluation MSA. Our novel *r*_20_ metric uniquely delivers that functionality, emphasizing higher-order covariation where previous studies rarely go beyond the pairwise level^2,17,21,50,74^.

Benchmarking coevolution-based protein sequence models in data rich and data poor regimes, as done here, is an effective method for ascertaining where data-driven effects stop, and algorithmic failure begins^75^. Due to the limited availability of natural protein sequence data, this line is inherently blurred in the natural analysis, as we demonstrate across all four of our generative capacity metrics. But in our synthetic analysis, we have demonstrated the extent to which VAEs, with different implementations, can capture higher-order covariation at orders between three and ten when the target probability distribution is known, its statistical properties are measurable with a high degree of certainty, and major forms of error are removed, minimized, or accounted for. When given a large number of training and target sequences, we found both VAEs’ generative capacity to be between that of a site-independent model (Indep) and a pairwise Hamiltonian (Mi3) for all measurements. In the synthetic *r*_20_ tests, our results show that both VAEs’ generative capacity are well below Mi3, raising questions about whether VAEs can capture higher-order epistasis significantly better than a pairwise Potts model. In our synthetic analysis the target probability distribution is a Potts model, and therefore we expect Mi3 to fit the target probability distribution well by design. However, we find Mi3 also outperforms the VAEs where we do not have this expectation, such as on the natural target probability distribution and on a target probability distribution specified by sVAE (see Supplementary Note 5), suggesting Mi3 generally outperforms VAEs on protein sequence data with respect to generative capacity.

The Hamming distance distributions, pairwise covariation correlations, and statistical energy correlations are standard metrics that have been used in the past to measure GPSM accuracy, but we find that they can be inadequate or misleading indicators of a GPSM’s ability to capture covariation at higher orders. Taken together, our results suggest that, of the metrics we tested, only *r*_20_ provides the granularity needed to discriminate between different GPSM’s ability to model higher-order epistasis, as it directly tests the model’s ability to capture higher-order covariation.

Though third and even fourth order sequence statistics have already been demonstrated^50,66–69^, the novelty of our *r*_20_ metric is that it generalizes and systematizes the assessment of a GPSM’s ability to model covariation into high orders, while recapitulating previous measurements for the lowest orders. Generalizing the measure in this way motivates comparison with current standard measures in machine learning literature for sequence-based and natural-language models, for instance perplexity. This provides additional perspective, showing how current measures do not probe higher-order covariation as directly as *r*_20_, and our results with *r*_20_ highlight how higher-order covariation can be used to distinguish models in ways these other measures cannot.

Although our results suggest VAE-GPSMs are less effective for capturing higher-order epistasis than pairwise Potts models, they have demonstrated utility in unsupervised learning and clustering. One VAE-GPSM, “BioSeqVAE”, has generated artificial sequences that share a “hallucinated” homology to natural proteins in the training set, which could mean that their folded structures would perform similar functions to their hallucinated natural homologs^28^. Another, “PEVAE”^26^, has shown that a VAE-GPSM’s latent space captures phylogenetic relationships better than PCA^76^ and t-SNE^77^. These VAE-GPSMs furnished a latent space that immediately allowed for function-based protein classification, a benefit unavailable to pairwise Potts models without some effort.

The causes of VAE performance limitations are actively being investigated in literature from multiple directions. One line of inquiry involves a VAE phenomenon known as “posterior collapse” (see Supplementary Note 3), in which some dimensions of the VAE latent space become insensitive to the input data. Studies of this phenomenon have led to some insights into VAE behavior, for instance that in some situations the VAE likelihood can contain spurious local maxima^78^, and many different heuristic strategies to understand and avoid this phenomenon have been suggested^79–81^. We test that sVAE, used in our main results, does not exhibit posterior collapse, though we can trigger it for sVAE architectures with more than seven latent dimensions in the bottleneck layer. Another line of inquiry relates to assumptions typically made about the metric and topology of the latent space, suggesting that the commonly used Euclidean metric space or Gaussian prior distribution may not best describe particular datasets, for instance because of an effect called “manifold mismatch”^82,83^. Techniques closely related to the VAE, such as the Wasserstein Autoencoder (WAE), are also being investigated as alternatives to the VAE, with improved performance characteristics^84,85^. Additionally, other sequence models, including transformer models and masked models^31,32,56,57^, can generate protein sequences, although these models are developed with different objectives and target datasets and are often not strictly GPSMs. In Supplementary Note 13, we test the generative ability of one such model, the MSA Transformer^31^, showing it is able to model mutational covariation to a limited degree. However, this model is not strictly a GPSM as defined above, and is not strictly comparable to the other models we test. Among these competing approaches, in this work we have tested VAE implementations which are currently used to generatively model protein sequences. With more nuanced latent variable models, and with better understanding of protein sequence embeddings, perhaps GPSM generative capacity could extend beyond what has been demonstrated here with state-of-the-art VAEs.

Our epistasis-oriented methodology focuses on measuring higher-order covariation, with the potential for broad applicability to various sequentially ordered data. *r*_20_-like measurements become possible when the data are sufficient in number, and the correlation structures between elements, both within and across samples, are statistically detectable and meaningful in some context, be it visual, biophysical, or linguistic. The convergence between data categories such as images, proteins, and language with respect to generative modeling evaluation offers the exciting opportunity of a wider, interdisciplinary audience for the work proposed here. Conversely, further development of sophisticated, data-intensive, and direct generative capacity metrics of GPSMs could reveal nuances of the correlation structure of protein sequence datasets that distinguish them from other datasets, helping to explain why *r*_20_-like metrics can detect higher-order covariation, whereas other metrics cannot. Our work represents not only a revision of currently prevailing paradigms of GPSM benchmarking, but also a challenge to generative protein sequence modeling more broadly, to consider how epistasis and direct higher-order covariation metrics like *r*_20_ can inform their models and results.

## Methods

### Sequence dataset preparation

For the natural analysis, we create MSAs containing sequences from the Uniprot/TREMBL database^63^ using the HHblits search algorithm^86^ and HMM seeds from the Pfam database^64^, for the protein kinase superfamily (PF00069), as well as the RRM (PF00076), response regulator (PF00072), and ABC transporter (PF00005) families. We filter the MSAs so that no sequence pair has greater than 50% identity, by iteratively selecting a random sequence and removing all similar sequences (see Supplementary Note 12). For all four protein families, we retain 20K sequences and randomly divide these into a 10*K* natural training MSA and 10K evaluation MSA. All of our natural training and evaluation MSAs are available in supporting information. For the synthetic analysis, we treat the Mi3 model trained on this natural protein kinase MSA as the target model or distribution, and generate MSAs from it, which serve as training and target MSAs in our synthetic tests. Despite coming from the same probability distribution, we are careful to ensure in each experiment that the training and target sequences are non-overlapping sets in order to mitigate overfitting (Fig. 1). Evaluation MSAs are generated by the GPSMs and used by our generative capacity measurements, which compare the evaluation MSA to the appropriate target MSA.

### Mi3

The Mi3 model is a pairwise Potts Hamiltonian model fit to sequence data using the “Mi3-GPU” software we have developed previously^43^, which performs “inverse Ising inference” to infer parameters of Potts models using a Markov-Chain Monte-Carlo (MCMC) algorithm which entails very few approximations. This software allows us to fit statistically accurate Potts models to MSA data. We have examined Mi3’s generative capacity and out-of-sample error in earlier work^43,65^, which we summarize here.

A Potts model is the maximum entropy model for *p*(*S*) constrained to reproduce the bivariate marginals ${f}_{\alpha \beta }^{{{{{{\rm{ij}}}}}}}$ of an MSA, i.e., the frequency of amino acid combination *α*, *β*, at positions *i*, *j*. The probability distribution *p*_θ_(*S*) for the Potts model takes the form

1 $${p}_{\theta }(S)=\frac{{e}^{-E(S)}}{Z}\quad \,{{\mbox{with}}}\,\quad E(S)=\mathop{\sum }\limits_{{{{{{\rm{i}}}}}}}^{L}{h}_{{{{{{{\rm{s}}}}}}}_{{{{{{\rm{i}}}}}}}}^{{{{{{\rm{i}}}}}}}+\mathop{\sum}\limits_{i < j}{J}_{{{{{{{\rm{s}}}}}}}_{{{{{{\rm{i}}}}}}}{{{{{{\rm{s}}}}}}}_{{{{{{\rm{j}}}}}}}}^{{{{{{\rm{ij}}}}}}},$$

where *Z* is a normalization constant, *Z* = ∑_S_*e*^−*E*(*S*)^, and “coupling” ${J}_{\alpha \beta }^{{{{{{\rm{ij}}}}}}}$ and “field” ${h}_{\alpha }^{{{{{{\rm{i}}}}}}}$ parameters are compactly denoted by the vector $\theta =\{{h}_{\alpha }^{{{{{{\rm{i}}}}}}},{J}_{\alpha \beta }^{{{{{{\rm{ij}}}}}}}\}$. The number of free parameters of the model (non-independent couplings and fields) is equal to the number of non-independent bivariate marginals, which can be shown to be $\frac{L(L-1)}{2}{(q-1)}^{2}+L(q-1)$ for *q* amino acids^19^, which is ~10.7M parameters for our model. This implies that the Potts model is well specified to reproduce the bivariate marginals when generating sequences from *p*_θ_(*S*).

The Mi3 model inference procedure maximizes the log-likelihood with regularization. Maximizing the Potts log-likelihood can be shown to be equivalent to minimizing the difference between the dataset MSA bivariate marginals ${f}_{\alpha \beta }^{{{{{{\rm{ij}}}}}}}$ and the model bivariate marginals of sequences generated from *p*(*S*). To account for finite sampling error in the estimate of ${f}_{\alpha \beta }^{{{{{{\rm{ij}}}}}}}$ for an MSA of *N* sequences we add a small pseudocount of size 1/*N*^65^. We also add a regularization penalty to the likelihood affecting the coupling parameters ${J}_{\alpha \beta }^{{{{{{\rm{ij}}}}}}}$ to bias them towards 0, of form $\lambda \sum {{{{{{{\rm{SCAD}}}}}}}}({J}_{\alpha \beta }^{{{{{{\rm{ij}}}}}}},\lambda ,\alpha )$ using the SCAD function which behaves like $\lambda | {J}_{\alpha \beta }^{{{{{{\rm{ij}}}}}}}|$ for small ${J}_{\alpha \beta }^{{{{{{\rm{ij}}}}}}}$ but gives no bias for large ${J}_{\alpha \beta }^{{{{{{\rm{ij}}}}}}}$^87^, using a small regularization strength of *λ* = 0.001 for all inferences which causes little model bias.

To generate synthetic MSAs from the Potts model, we use MCMC over the trial distribution *p*_θ_(*S*) until the Markov-Chains reach equilibrium^43^. We can directly evaluate *E*(*S*) as the negative log-probability of any sequences for the Mi3 model using Eq. (1) up to a constant *Z*, and this constant can be dropped without affecting our results.

### Indep

The Indep model is the maximum entropy model for *p*(*S*) constrained to reproduce the univariate marginals of an MSA, and is commonly called a “site-independent” model because the sequence variations at each site are independent of the variation at other sites. Because it does not fit the bivariate marginals, it cannot model covariation between positions. It takes the form

2 $${p}_{\theta }(S)=\frac{{e}^{-E(S)}}{Z}\quad \,{{\mbox{with}}}\,\quad E(S)=\mathop{\sum }\limits_{{{{{{\rm{i}}}}}}}^{{{{{{\rm{L}}}}}}}{h}_{{{{{{{\rm{s}}}}}}}_{{{{{{\rm{i}}}}}}}}^{{{{{{\rm{i}}}}}}},$$

where *Z* is a normalization constant, *Z* = ∑_S_*e*^−E(S)^, and “field” parameters ${h}_{\alpha }^{{{{{{\rm{i}}}}}}}$ for all positions *i* and amino acids residue *α* are compactly referred to by the vector $\theta =\{{h}_{\alpha }^{{{{{{\rm{i}}}}}}}\}$. The fields of the Indep model generally have different values from the fields of the Potts model. Unlike for the Potts model, maximum likelihood parameters can be determined analytically to be ${h}_{\alpha }^{{{{{{\rm{i}}}}}}}=-{{{{{{\mathrm{log}}}}}}}\; {f}_{\alpha }^{{{{{{\rm{i}}}}}}}$ where ${f}_{\alpha }^{{{{{{\rm{i}}}}}}}$ are the univariate marginals of the dataset MSA. When fitting the Indep model to a dataset MSA of *N* sequences, we add a pseudocount of 1/*N* to the univariate marginals to give model marginals ${\hat{f}}_{\alpha }^{{{{{{\rm{i}}}}}}}$, to account for finite sample error in the univariate marginal estimates. The model distribution simplifies to a product over positions, as ${p}_{\theta }(S)=\sum {\hat{f}}_{{{{{{{\rm{s}}}}}}}_{{{{{{\rm{i}}}}}}}}^{{{{{{\rm{i}}}}}}}$. The number of independent field parameters is *L*(*q* − 1) which equals ~4.6K parameters for our model.

To generate sequences from the independent model we independently generate the residues at each position *i* by a weighted random sample from the marginals ${f}_{\alpha }^{{{{{{\rm{i}}}}}}}$, and we directly evaluate the log probability of each sequence *E*(*S*) from Eq. (2).

### VAEs

The standard variational autoencoder (sVAE) is a deep, symmetrical, and undercomplete autoencoder neural network composed of a separate encoder *q*_ϕ_(*Z*∣*S*) and decoder *p*_θ_(*S*∣*Z*)^88^, which map input sequences *S* to regions within a low-dimensional latent space *Z* and back. The probability distribution for the sVAE is defined as

3 $${p}_{\theta }(S)=\int {p}_{\theta }(S| Z)p(Z){{{{{\rm{d}}}}}}Z,$$

where the latent space distribution is a unit Normal distribution, $p(Z)={{{{{{{\mathcal{N}}}}}}}}[0,1](Z)$. Training of a VAE can be understood as maximization of the dataset log-likelihood with the addition of a Kullback–Leibler regularization term D_KL_[*q*_*ϕ*_(*Z*∣*S*), *p*_θ_(*Z*∣*S*)], where *p*_θ_(*Z*∣*S*) is the posterior of the decoder^39,40^.

sVAE’s architecture is “vanilla”^89^, meaning it is implemented in a standard way and its behavior is meant to be representative of VAEs generally. A nearly identical architecture was used previously as a VAE-GPSM in “EVOVAE”^27^. sVAE’s encoder and decoder are implemented without advancements such as convolutional layers^25^, multistage training^90^, disentanglement learning^89^, Riemannian Brownian motion priors^91^, and more. This allows us to directly interrogate the assumptions and performance of standard variational autoencoding with respect to the training and evaluation of GPSMs in this work. As with EVOVAE, sVAE architecture and hyperparameters were selected via grid search hyperparameter tuning. Detailed discussions of sVAE architecture, hyperparameters, tuning, and nuances between EVOVAE and sVAE are available in Supplementary Note 1.

Employing standard normalization and regularization strategies as needed^92,93^, our encoder and decoder have three hidden layers each, with 250 nodes per hidden layer. sVAE’s latent bottleneck layer has seven nodes, and the model in total has 2.7M inferred parameters. The input layer of the encoder accepts one-hot encoded sequences with a mini-batch size of 200, and the decoder’s output layer values can be interpreted as a Bernoulli distribution of the same dimensions as a one-hot encoded sequence. We tested various sVAE architectures and hyperparameters with our datasets, as well as DeepSequence as described below, and found qualitatively similar generative capacity results with sVAE.

To generate a sequence from sVAE, we generate a random sample in latent space from the latent distribution *p*(*Z*) and then pass this value to the decoder to obtain a Bernoulli distribution, from which we sample once. To evaluate the negative log-probability of a sequence *E*(*S*) we use importance sampling, averaging over 1K samples from the latent distribution *q*_ϕ_(*Z*∣*S*)^26^. Other publications use the evidence lower bound (ELBO) estimate as an approximation of the negative log-probability^25^, and we have verified that the ELBO and the negative log-probability are nearly identical in our tests and have equal computational complexity (see Supplementary Note 2).

In addition to sVAE, we test the DeepSequence VAE^25^. We use the default inference parameters, and use the “SVI” inference implementation which uses a “variational Bayes” inference technique as an extension of the sVAE inference method. Because DeepSequence is designed to output the ELBO rather than the negative log-probability for each sequence, we use the ELBO as an approximation of *E*(*S*), and estimate it using an average over 1K samples. To generate sequences we use the same strategy as for sVAE.

### Reporting summary

Further information on research design is available in the Nature Research Reporting Summary linked to this article.

## Supplementary information

Supplementary Information

Peer Review File

Reporting Summary

## Data Availability

The target and training MSAs used in the natural analysis for the four tested protein families are available in Supporting Information. These are generated based on sequences in the Unitprot database^63^, using seeds from the Pfam database PF00069, PF00076, PF00072, PF00005. All other intermediate values are computable from these, and are also available from the corresponding authors on reasonable request.

## Code availability

The source codes required to reproduce the results in this manuscript are available at the links listed below. Generative capacity metrics: https://github.com/alagauche/generative_capacity_metrics v1.0. sVAE: https://github.com/ahaldane/MSA_VAE v1.0 Mi3: https://github.com/ahaldane/Mi3-GPU v1.1 *r*_20_ and cc − *r*_20_: https://github.com/ahaldane/HOM_r20 v1.0 Sequence generator for MSA Transformer: https://github.com/S-Hauri/MSA_Transformer_Generator v1.0. This code additionally uses the following python packages: Numpy v1.20.1^94^, Pandas v1.0.5^95^, Matplotlib v3.34^96^, Tensorflow v2.2.0^97^. We use HHblits v3.1.0 for sequence search^86^.
