# Supplementary material for: The generative capacity of probabilistic protein sequence models
Source: Nat Commun. 2021 Nov 2;12:6302. doi: 10.1038/s41467-021-26529-9 (PMC8563988; doi:10.1038/s41467-021-26529-9)
Supplement: Supplementary file 1 — Supplementary Information [file 41467_2021_26529_MOESM1_ESM.pdf]

Supplementary Information for:
Generative Capacity of Probabilistic Protein Sequence Models

Francisco McGee Sandro Hauri Quentin Novinger Slobodan Vucetic Ronald M. Levy
Vincenzo Carnevale Allan Haldane

Supplementary Note 1 - sVAE implementation

The standard variational autoencoder (sVAE) is a deep, symmetrical, and undercomplete autoencoder neural network composed of a separate encoder $q_\phi(Z|S)$ and decoder $p_\theta(S|Z)$[1], which map input sequences S into regions of a low-dimensional latent space Z and back (see Fig. S1). It is a probabilistic model, and in our "vanilla"[2] implementation we assume sequences will be distributed according to a unit normal distribution in latent space, $p(Z) = \mathcal{N}[0,1](Z)$[3]. Training of a VAE can be understood as maximization of (the logarithm of) the dataset likelihood $\mathscr{L} = \prod_S p_\theta(S) = \sum_S \int p_\theta(S|Z)p(Z)dZ$ with the addition of a Kullback-Leibler regularization term $\mathrm{D_{KL}}[q_\phi(Z|S), p_\theta(Z|S)]$, where $p_\theta(Z|S)$ is the posterior of the decoder, which allows use of the fitted encoder $q_\phi(Z|S)$ to perform efficient estimation of the likelihood and its gradient by Monte-Carlo sampling, for appropriate encoder models. The sVAE architecture is built on the same basic VAE architecture of "EVOVAE"[4], which itself appears to be built on the VAE implementation provided by developers for the Keras library[5], and this same VAE architecture is used for each protein presented in this work.

Similarly to EVOVAE, sVAE's hyperparameters were tuned using grid search. sVAE is composed of 3 symmetrical ELU-activated layers in both the encoder and decoder, each layer with 250 dense (fully-connected) nodes. The encoder and decoder are connected by a latent layer of l nodes, and we use $l = 7$ in the main text. We provide further justification for the selection of $l = 7$ elsewhere in the Supplementary Note 3. sVAE's input layer accepts one-hot encoded sequences, the output layer is sigmoid-activated, and its node output values can be interpreted as a Bernoulli distribution of the same dimensions as a one-hot encoded sequence. The first layer of the encoder and the middle layer of the decoder have dropout regularization applied with 30% dropout rate, and the middle layer of the encoder uses batch normalization[4,6,7].

In all inferences, we hold out 10% of the training sequences as a validation dataset, and perform maximum likelihood optimization using the Keras Adam stochastic gradient optimizer on the remaining 90%[8], using mini-batch gradient descent with a batch size of 200. After each training epoch we evaluate the loss function for the training and validation data subsets separately. We have tested using early-stopping regularization to stop inference once the validation loss has not decreased for three epochs in a row, as in previous implementations, but this led to some variability in the model depending on when the early stopping criterion was reached. To avoid this variability, and to make different models more directly comparable, we instead fixed the number of epochs to 32 for all models, since in the early stopping tests this led to near minimum training loss and validation loss, and did not lead to significant overfitting as would be apparent from an increase in the validation loss.

sVAE was implemented using Keras, building on previous implementations[4,5], however with a modification of the loss function relative to both of these, to remove a scaling factor of Lq on the reconstruction loss, which is sometimes used to avoid issues with local minima as described further below. This prefactor leads to a non-unit variance of the latent space distribution of the dataset sequences, violating our definition that the latent space distribution should be normal with unit variance, $p(Z) = \mathcal{N}[0,1](Z)$. In the next section we show that after removing the prefactor the latent space distribution is approximately a unit normal, which more closely follows the original VAE conception[3,9]. Our implementation is available at `https://github.com/ahaldane/MSA_VAE`.

To generate a sequence from the model we generate a random sample in latent space from the latent distribution $\mathcal{N}[0,1]$, and pass this value to the decoder to obtain a Bernoulli distribution, from which we sample once.

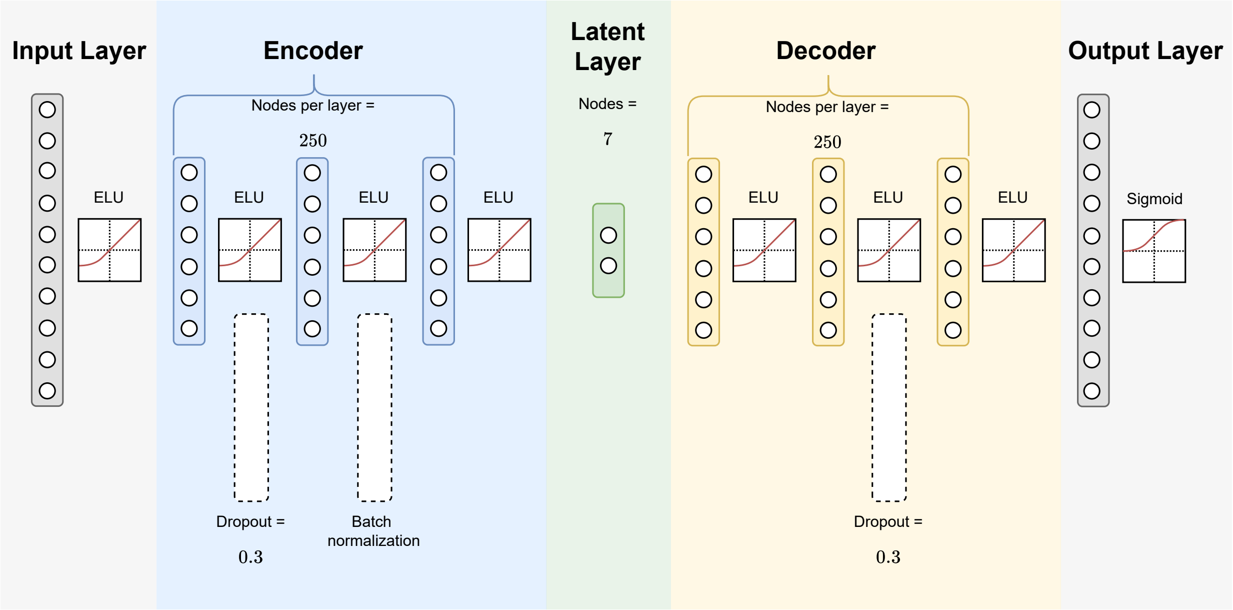

Figure S1. sVAE architecture diagram. Our standard VAE (sVAE) is a vanilla variational autoencoder with standard regularization. Both input and output layers (**grey**) have $L*q$ nodes, where L is the length of the protein MSA and q is the amino acid alphabet of size 21. Both the encoder (**blue**) and decoder (**yellow**) have 3 hidden layers each, and each layer has 250 nodes. For regularization (dashed lines), a 30% dropout layer of has been placed between layers 1 and 2 of the encoder, and layers 2 and 3 of the decoder. Except for the latent layer (**green**) and the output layer, all layers are ELU-activated. The latent layer, also called the "bottleneck" layer, has 7 nodes, meaning sVAE has 7 latent variables, and does not have an activation function. The output layer is sigmoid-activated. sVAE retains the same architecture for all proteins modeled in this work.

Supplementary Note 2 - Estimating $E(S)$ for sVAE by Monte-Carlo

To evaluate the log-probability of a sequence, we use importance sampling, averaging over 1000 samples from the latent distribution $q_\phi(Z|S)$ following from the relations[10,11]

$$p_\theta(S) = \int p_\theta(S|Z)p(Z)dZ = \int q_\phi(Z|S)\frac{p_\theta(S|Z)p(Z)}{q_\phi(Z|S)}dZ$$
$$= \underset{Z\sim q_\phi(Z|S)}{\mathbb{E}}\left[\frac{p_\theta(S|Z)p(Z)}{q_\phi(Z|S)}\right] \approx \frac{1}{N}\sum_i^N \frac{p_\theta(S|Z^i)p(Z^i)}{q_\phi(Z^i|S)} \quad (1)$$

where, Z^i are independent samples from $q_\phi(Z|S)$ and N is a large number of samples. Here $q_\phi(Z|S)$ plays the role of a sampling bias function, biasing samples to regions of latent space which are likely to have generated the sequence, leading to an accurate Monte-Carlo estimate of $p_\theta(S)$. The value $p_\theta(S)$ can be converted to a unit-less statistical energy as $E(S) = -\log p_\theta(S)$ for direct comparison with Mi3 and Indep statistical energies.

Other publications have used the Evidence Lower Bound (ELBO) estimate as an approximation of $\log p_\theta(S)$[12], and we have tested that the ELBO and the log-probability are nearly identical (see Fig. S2). The fact that the ELBO and log-probability are nearly identical is a sign that our encoder is well fit, as the difference between these values should equal the KL divergence $\mathrm{D_{KL}}[q_\phi(Z|S), p_\theta(Z|S)]$ between the "true" posterior of the decoder $p_\theta(Z|S)$ and the approximate posterior $q_\phi(Z|S)$, which should be 0 if the encoder $q_\phi(Z|S)$ has accurately modelled the posterior[3]. This test was performed using $N = 1000$ samples for both calculations, and the close correspondence between the two calculations additionally suggests that the Monte-Carlo error in the estimates is low for this N, and so our conclusions related to $E(S)$ in the main text are well supported.

To further investigate whether $N = 1000$ samples are enough to reliably estimate $E(S)$, we perform an additional test to measure the error in $E(S)$ as a function of N (S2, right). In this test, we take $N = 100K$ Monte-Carlo samples of the r.h.s. term of equation 1 for each of a set of 200 kinase sequences, and define $\bar{E}(S)$ to be the estimate of $E(S)$ using equation 1 using all $100K$ samples. Then, we repeat the estimate of $E(S)$ but by using a limited number N of the $100K$ samples, giving an estimate $E_N(S)$. For each value N for each sequence, we repeat this estimation of $E_N(S)$ for $k = 10$K times for different random choices of the N samples out of the $100K$. In total we have k estimates $E_{N,k}(S)$ for each sequence for each value N. We then measure the error relative to the estimate made with all the samples $\bar{E}(S)$. We measure the error in two ways: firstly, the root mean square (RMS) error in the energy,

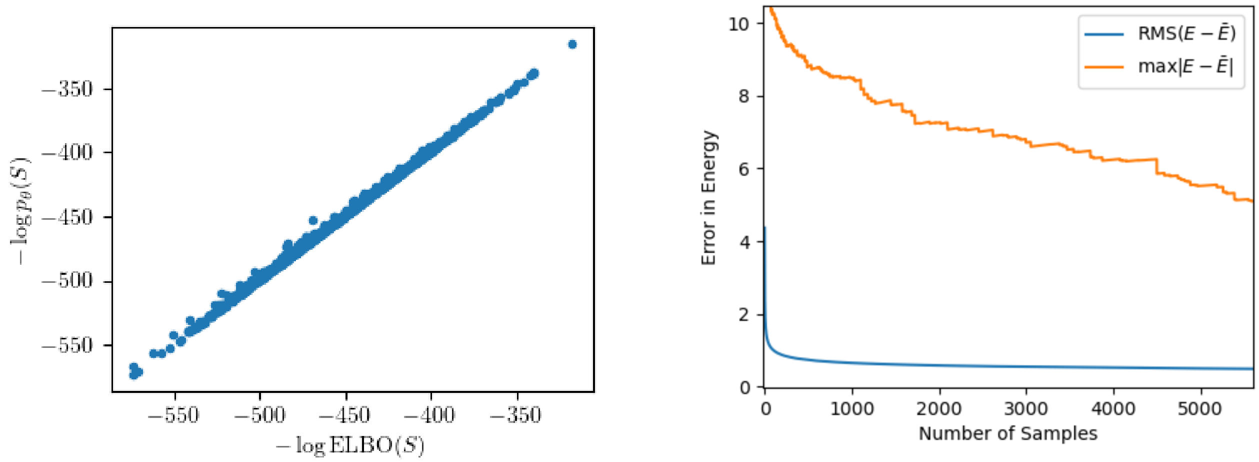

Figure S2. Left: Comparison of $E(S) = -\log p_\theta(S)$ with the ELBO estimate for the sVAE with $l = 7$ fit to $1M$ sequences, evaluated for 1000 sequences S from the validation dataset, with $N = 1000$ samples for both the ELBO estimate and the $E(S)$ estimate. Right: Analysis in the Monte-Carlo estimator error in $E(S)$ for sVAE, as a function of the number of samples N used in equation 1. Two types of error are shown: the root mean square (RMS) error for $E(S)$ estimated using N samples relative to the estimated energy using $\bar{E} = 100,000$ samples, and the maximum absolute error in $E(S)$ relative to $\bar{E}$ observed over $10K$ random realizations of the estimate. See text for details.

$(\sum_{k,S}(E_{N,k}(S) - \bar{E}(S))^2)^{1/2}$, averaging over all sequences and k, and 2. the max observed error, $\max_{k,S}|E_{N,k}(S) - \bar{E}(S)|$, maximized over all sequences and k.

We find that the RMS error in $E(S)$ becomes less than 1 even at a very small number of samples, $N = 101$. At $N = 1000$, as we used in the main text, the RMS error in the energy is 0.6 energy units. This is much smaller than the RMS error in the predicted energy (after subtracting the mean offsets) for the sVAE relative to the target energy in our synthetic tests, for instance of 78 energy units for Figure 5E in the main text for our $1M$ synthetic test. It is also small relative to the overall range of sequence energies, of about 200 energy units. This suggests that the amount of error due to the Monte Carlo sampling is a negligible source of error in our results for sVAE, and that $N = 1000$ samples is sufficient to support our conclusions in the main text.

The max error measurement represents the extremely rare "worst case" scenario. This is the maximum difference we observed for $E_{N,k}(S)$ relative to the large-sample estimate $\bar{E}(S)$, over all $k = 10K$ realizations. This rare worst-case error is higher than the RMS error, and at $N = 1000$ samples is 8 energy units. This is still much smaller than the RMS error of the sVAE relative to the target in our synthetic tests, so our conclusions using $N = 1000$ in the main text are well supported. The fact that the "worst case" error is significantly larger than the RMS error suggests a edge case phenomenon. Indeed, we observe (not shown) that in some cases sVAE generates a small number of rare outlier samples which contribute an unusually high likelihood score, and these outliers can contribute significantly to the average performed in equation 1. Nevertheless, the max error measurement shows that the Monte-Carlo error in our estimates of $E(S)$, even in the worst case, is still small relative to the sVAE model's specification error.

Supplementary Note 3 - VAE model validation and hyperparameter selection

To validate our choice of latent space size of $l = 7$ used in the main text, we tried fitting sVAEs with different latent space sizes from 2 to 10. In Fig. S3 we illustrate the 2D pairwise latent space projections of the sequences in the training dataset, where each latent variable is plotted against each of the others. In keeping with the vanilla VAE implementation[3], we let the prior over the latent variables be the centered isotropic multivariate Gaussian $p_\theta(z) = \mathcal{N}(z; 0, I)$, and so we expect the latent space distribution to be a multidimensional normal distribution with mean 0 and unit variance. Indeed, as can be seen in the plot and measured numerically, we generally find the latent space distribution of the dataset has close to unit variance and is approximately normal, although there is some non-normal structure in the distribution.

For latent spaces of $l = 8$ and $l = 10$ we observe that some latent dimensions appear to have"collapsed", in particular z_0 for $l = 8$, and z_1 and z_6 for $l = 10$. From repeated runs (not shown) we observe that the number of collapsed dimensions varies somewhat depending on the random seed used to initialize the stochastic optimizer, and also depends on the size of the training dataset as more dimensions collapse when fitting $1M$ sequences than

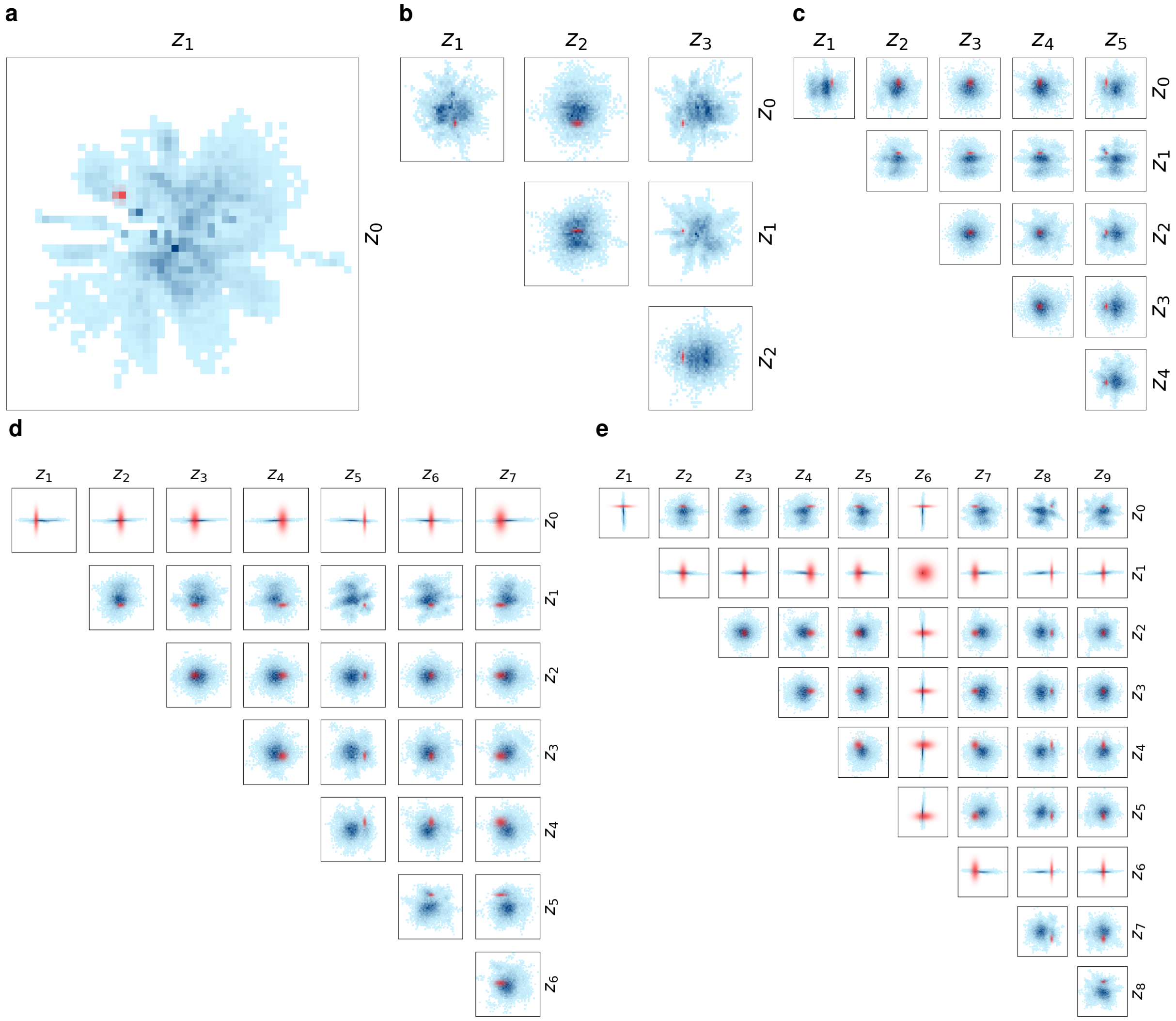

Figure S3. Plots of latent space distribution of the training dataset for sVAE models fit with different latent space sizes of 2, 4, 6, 8, and 10 (**a**,**b**,**c**,**d**,**e** respectively), fit to 1M synthetic training sequences as in the synthetic test in the main text. For each latent space size we show, for each pair of latent variables, a 2d histogram of the projected means of $10K$ training dataset sequences in latent space in blue. There is one subplot for $l = 2$, six subplots for $l = 4$, etc. Each plot ranges from -4 to 4 on both axes. The latent distribution $q_\phi(Z|S)$ for single random sequence from the training dataset is shown as a red shading in proportion to probability.

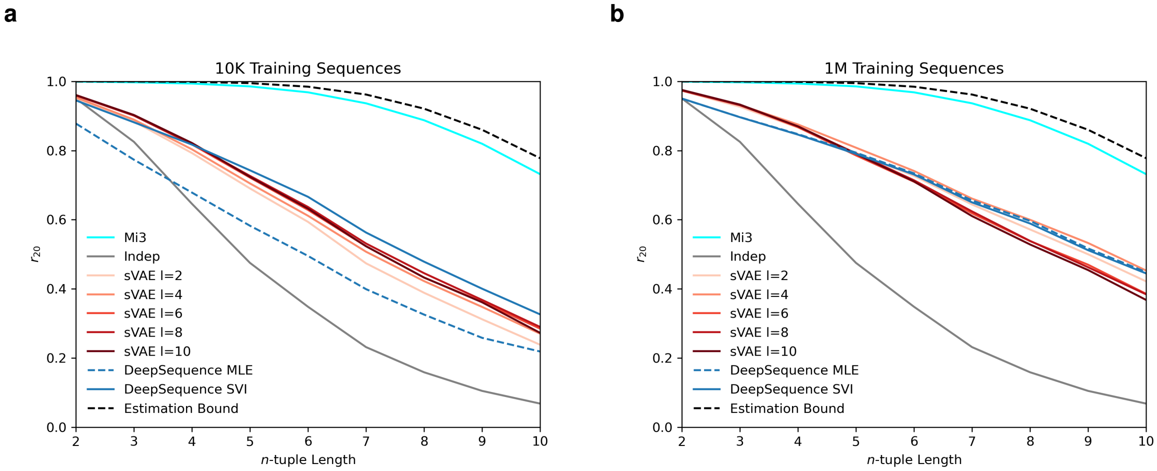

Figure S4. Performance comparison of sVAEs for different l compared to DeepSequence VAEs and Mi3 using the r_{20} metric on $10K$ synthetic (**a**) and $1M$ synthetic (**b**) training sequences.

fitting $10K$ sequences (not shown). For these "collapsed" dimensions, we see that the projected variance of the illustrated sequence in red in Fig. S3 is very close to 1, unlike in other dimensions where the projected variance is much smaller. These observations are consistent with "posterior collapse", a phenomenon discussed in VAE literature[13]. It has been suggested that VAE posterior collapse can occur due to local minima in the likelihood function[13], but in some situations can be a sign that additional latent dimensions are uninformative, and that fewer latent dimensions better represent the data[14]. We find that choosing $l = 7$ gives the best performing model according to our generative capacity metrics, which also avoids posterior collapse. Interestingly, the number of "informative" latent variables, i.e. those that do not undergo posterior collapse, turns out to coincide with the intrinsic dimension (ID) of the natural kinase dataset of training sequences, estimated from the set of pairwise distances using a completely independent approach[15]. In brief, it has been shown that graph distances calculated on k-neighbor graphs can be used to approximate geodesics and thus to generate the distribution of "intrinsic" distances. Close to the maximum, the latter depends exclusively on the dimension of the distance distribution's support. This observation is used to devise a family of estimators for the ID. Using these tools, we estimated an ID of 7 or 8 for the synthetic dataset used in the main text. These numbers are consistent with what was observed in terms of collapse of the posterior distribution: the ID is seemingly related to the number of informative latent variables so that if the number of nodes in the embedding layer is increased past this number, then posterior collapse occurs, indicating that the additional variables are not needed to explain the data. While ID analysis was performed only on the natural kinase dataset, it appears from our generative capacity measurements on other proteins elsewhere in Supplementary Notes 3, 11 that $l = 7$ was an appropriate choice for the other natural protein datasets. A rigorous and systematic explanation of the relationship between the choice of l embedding dimensions for a VAE and various protein families is an intriguing possibility, but falls outside the scope of this work.

To compare the generative capacity of different GPSMs to determine how general our results are, we computed our MSA statistics for other VAEs besides the $l = 7$ sVAE shown in the main text. In Fig. S4 we show the r_{20} scores for different models when fit to either $10K$ or $1M$ synthetic sequences, as in the synthetic tests in the main text. We include the Mi3 and Indep models, as well as sVAEs for different latent space sizes, and also models produced using the DeepSequence VAE software which comes in two variations, the "MLE" and the "SVI" algorithms[12], for which we use the default or example parameters. All the VAEs perform fairly similarly in this metric, including the DeepSequence VAEs. For the smaller training dataset of $10K$ sequence the DeepSequence SVI algorithm outperforms the other VAEs, suggesting it is less susceptible to out-of-sample error. These results suggest that our results for the sVAE shown in the main text generalize to other VAEs, including the significantly more complex DeepSequence VAE, and are not strongly dependent on implementation or number of latent variables l. The models with $l \sim 7$ perform among the best of the sVAE models for both the $10K$ and the $1M$ training datasets, though the difference between the models is small, and this further justifies our choice of $l = 7$ in the main text.

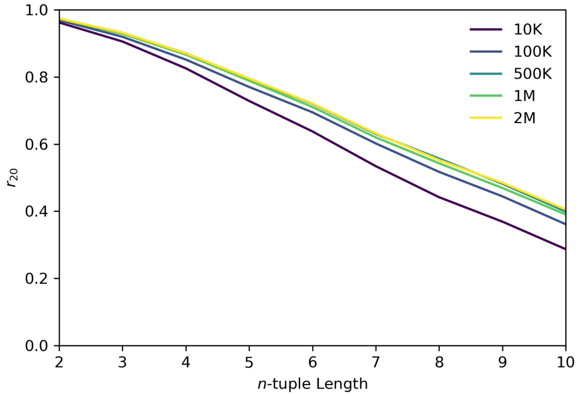

Figure S5. sVAE performance for $l = 7$ for varying synthetic training dataset sizes. For each training dataset size, two inferences are run with different random seeds, shown in solid and dashed lines for each training size. These results demonstrate how varying l for sVAE and algorithm choice (SVI vs MLE) for DeepSequence affect model performance.

Supplementary Note 4 - $1M$ training Sequences are sufficient to virtually eliminate out-of-sample error

Since the goal of the synthetic test with $1M$ training sequences in the main text is to eliminate out-of-sample error (overfitting) by using an extremely large training dataset, we demonstrate here how large the training dataset must be to mostly eliminate out-of-sample error for the sVAE. In Fig. S5 we show tests for the $l = 7$ sVAE for increasing training dataset sizes, finding that after $500K$ sequences the improvement in performance becomes small. This justifies our choice of using $1M$ synthetic training sequences, as there is little additional improvement to be gained by fitting to $2M$ sequences at the cost of increasingly prohibitive fitting time.

We have previously published numerical and analytic results exploring out-of-sample-error (overfitting) in Potts models, which are consistent with these observations. Both the sVAE and the Potts model have a large number of parameters ($3M$ and $10M$ respectively) which is larger than the number of sequences used to train the model, which might raise questions of overfitting. However, in Ref. 16 it is shown how, contrary to common intuition, the degree of overfitting (i.e. out-of-sample error) of Potts models cannot be gauged by simply comparing the number of parameters to the number of sequences. Overfitting also depends on the level of sequence conservation, and its effect can be estimated analytically as a function of the sequence length (L), number of sequences in the MSA (N), and the degree of conservation of the MSA (a quantity called χ in Ref. 16). In Ref. 16 we show that out-of-sample error is virtually eliminated in some conditions even when the number of training sequences is orders of magnitude smaller than the number of parameters of the model, which is illustrated this with some simple examples. In summary, this analysis supports the results of Fig. S5 and provide an explanation for why one should expect $1M$ sequences to be sufficient to virtually eliminate out-of-sample error, even though the number of model parameters is larger.

Supplementary Note 5 - Using sVAE as the synthetic target probability distribution

In the main text, our synthetic GPSM tests are performed using a Potts model as the synthetic target probability distribution. This means that the synthetic target probability distribution is constructed without higher-order interaction terms, and a Potts model is by definition well specified to fit data generated from this target probability distribution. This opens up the possibility that Mi3 could outperform other GPSMs, such as the VAE, in the synthetic analysis for this reason alone, rather than because of having lower specification error. Here, we show GPSM performance when the synthetic target probability distribution instead corresponds to a sVAE, which potentially generates data which cannot be fit by a model with only pairwise interaction terms. If Mi3 were to fit the sVAE distribution better than sVAE itself, then this would be compelling evidence that Mi3 had no unfair advantage in our synthetic experiments, and that indeed Mi3 has lower specification error.

In this section we take the synthetic target probability distribution to be described by the sVAE fit in the main text from $10K$ natural sequences. As described in the main text, this model potentially generates patterns of higher-order mutational covariation which cannot be fit by the Mi3 model. We then follow the same procedure as for our synthetic $1M$ test of the main text, but using this target probability distribution. We generate $1M$ sequences from the target

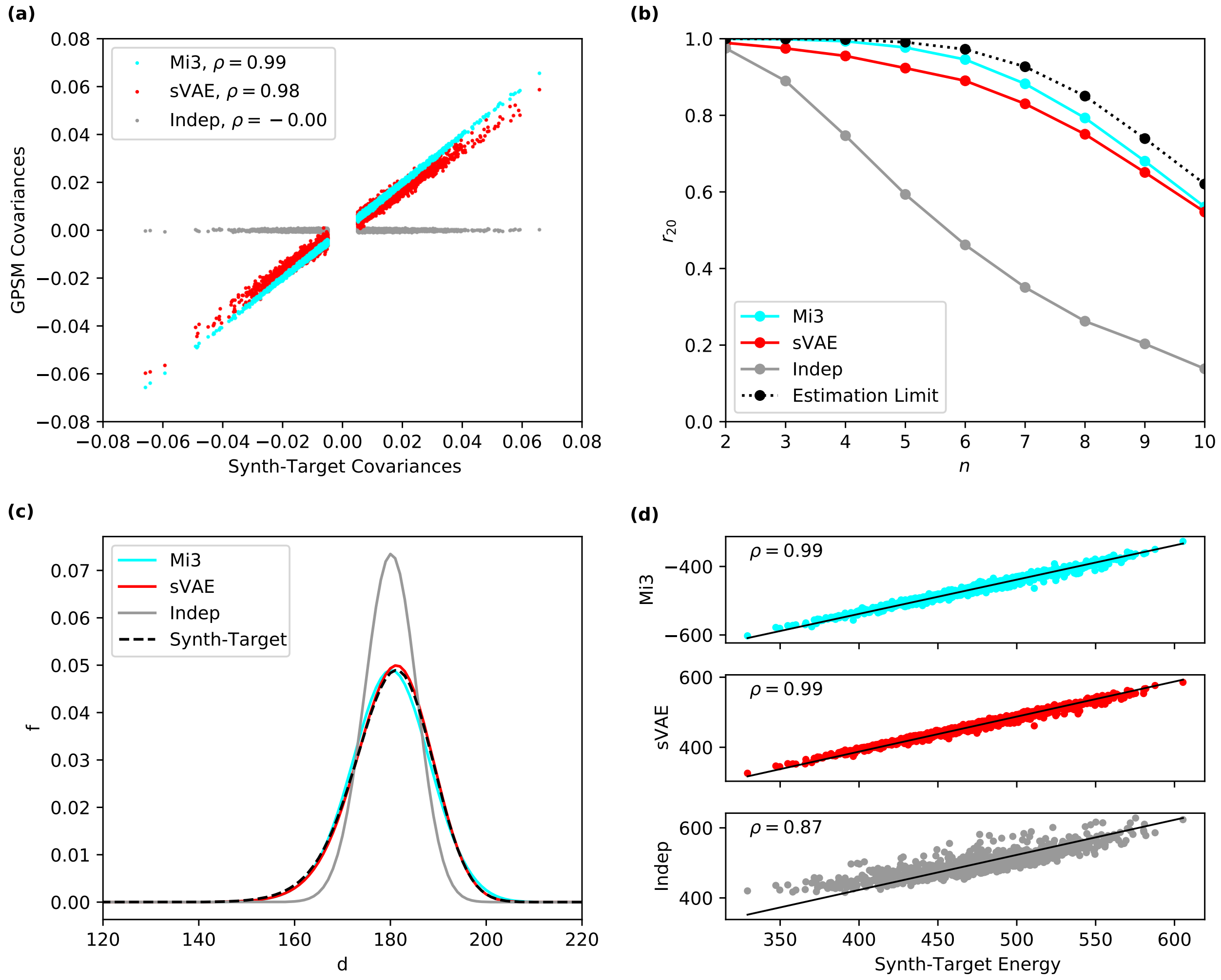

Figure S6. Synthetic test of the performance of different GPSMs when the synthetic target probability distribution is specified by sVAE. **a** Pairwise covariance correlation scores, as in main text Figure 2a. **b** r_{20} scores, as in main text Figure 3a. **c** Hamming distance distributions, as in main text Figure 4a. **d** Statistical energy scores, as in main text Figure 5, panels a, c, e.

sVAE distribution which we use as training data for each GPSM, that is for Mi3, sVAE, and Indep. We generate evaluation MSAs from each inferred model and compare it to evaluation MSAs generated from the target probability distribution, using our test statistics.

In Fig. S6 we show MSA test statistics for the models fit to the sVAE target. We find that the performance of Mi3 fit to this target performs at least as well as the sVAE model fit to the same target. As in the main text $1M$ synthetic test, the correlation scores are estimated from $500K$ evaluation sequences from the target and each GPSM, the r_{20} scores using $6M$ evaluation sequences, the Hamming distributions from $30K$ sequences, and the energies are evaluated for $1K$ sequences using 1000 Monte Carlo samples. For the r_{20} test we measure the estimation limit due to the finite size of the evaluation MSAs by computing r_{20} between two MSAs of size $6M$ generated from the target probability distribution. There is a small difference between the estimation error limit and the Mi3 result, which may be due to out-of-sample error due to the finite $1M$ training data, or due to specification error, and this difference is smaller than the difference of sVAE fit to the same target probability distribution (red). In sum, we interpret these tests to show that the Mi3 model is better able to fit the sVAE's target probability distribution than sVAE itself, showing that the experiments performed in the synthetic analysis were not biased in favor of Mi3.

Supplementary Note 6 - How higher-order covariation is represented by pairwise models

One of the questions we address in the main text is whether different GPSMs are well specified to describe protein sequence variation, especially in the case of covariation of many positions in the sequence at once. Of particular interest is whether a model which explicitly includes only pairwise interactions, such as the Potts model, is sufficient to model higher order epistasis, or whether GPSMs with more complex functional forms, such as a VAE, are necessary.

For clarity, we give a brief example describing how Potts models can predict many patterns of higher-order covariation, meaning triplet and higher patterns of residue covariation, despite only modelling pairwise interactions. We illustrate this using a toy model describing sequences of length $L = 3$ with two residue types A and B, with $2^3 = 8$ possible sequences, and show different forms of higher-order covariation which a pairwise model can and cannot fit. Detailed discussion and theoretical results suggesting why pairwise models are often sufficient to model many datasets have been provided by others[17–19].

First, we show how such a Potts model generates triplet covariation. Consider a Potts model with parameters given by $J^{12}_{AA} = J^{23}_{AA} = -s$ for some interaction strength s and all other field and coupling parameters are 0. This directly couples the character "A" between positions $(1,2)$ and also positions $(2,3)$. These interactions cause pairwise covariation between the directly coupled residues, and in the limit of large s we find $C^{12}_{AA} = C^{23}_{AA} = 0.08$, or 8%, but they also cause covariation between the indirectly coupled pair, as $C^{13}_{AA} = 0.04$, or 4%. Furthermore, this Potts model predicts three-body covariation, as can be seen by computing the three-body connected covariation terms found in cluster expansions in statistical physics given by

$$C^{123}_{\alpha\beta\gamma} = f^{123}_{\alpha\beta\gamma} - f^{1}_{\alpha}C^{23}_{\beta\gamma} - f^{2}_{\beta}C^{13}_{\alpha\gamma} - f^{3}_{\gamma}C^{12}_{\alpha\beta} - f^{1}_{\alpha}f^{2}_{\beta}f^{3}_{\gamma} \quad (2)$$

and we find that $C^{123}_{AAA} = 0.024$, or 2.4%, which is nonzero. This shows that a Potts model generates and can fit higher-order covariation between sets of residues even though the interactions are only pairwise, as a result of indirect covariation through chains and loops of pairwise interactions.

AAA
ABB
BAB
BBA

Table 1. Example MSA following the XOR pattern.

An example of MSA triplet statistics which a Potts model is mis-specified to describe is the XOR pattern in which the dataset is composed in equal proportions of copies of the four sequences shown in Table 1. These sequences follow the XOR function in boolean logic, so that the third position is the XOR function applied to the first two positions. One can see that both the A and B residues have a 50% probability at each position, and that for each pair of positions the probability of each of the four combinations AA, AB, BA, BB is $1/4$. This means that the pairwise covariances $C^{ij}_{\alpha\beta} = 0.25 - 0.5 \times 0.5$ are all 0. Because there are no pairwise covariances, fitting a Potts model to this data will yield a model with no coupling terms, equivalent to an Indep model. Sequences generated from this (or any) Indep model have all three-body covariation terms equal to 0. However, the three-body covariations of the dataset are non-zero and $C^{123}_{AAA} = 0.125$. This shows how a Potts model fit to XOR data will fail to reproduce the correct three-body covariations. More generally, it will fail to model data which follows a boolean parity function, which generalizes the XOR function to longer strings, and is defined so that the last character is set to "B" if there are an odd number of "B" characters in the preceding sequence.

A motivation for the VAE is that it may potentially be able to model patterns of covariation such as the XOR pattern which a Potts model cannot. Whether a VAE is able to outperform the Potts model when fit to protein sequence data will depend on the prevalence of patterns such as XOR in the data which cannot be fit by a Potts model. If they are undetectable, the Potts model will be well specified and third order parameters are unnecessary. Our results with the natural dataset in the main text suggest the Potts model is able to reproduce the MSA statistics we tested up to the limits imposed by estimation and out-of-sample error.

Supplementary Note 7 - Connected correlation cc-r_{20} score for higher-order covariation

In addition to the r_{20} metric, we also consider close variants of it. Here we test one variant in which we use higher-order "connected-correlations" rather than marginals in an r_{20}-like calculation.

The connected-correlations, or Ursell functions[20], arise in statistical physics of many-body systems including Ising and Potts models. The second order connected-correlation is equal to the covariation scores $C^{ij}_{\alpha\beta}$, and higher orders of the connected-correlations functions g can be defined recursively by

$$g_{[p]} = f_{[p]} - \sum_{\mathscr{P}[p]} \prod_{p_i \in \mathscr{P}} g_{[p_i]} \tag{3}$$

where $[p]$ is a set of indices $1...p$, $f_{[p]}$ is the higher-order marginal of interest, and $\mathscr{P}[p]$ is the set of partitions of $[p]$ into groups of indices, not including the original set $[p]$. In our case, a marginal such as f_{123} is the marginal for a particular word, in this case of of length 3, which we noted as f^{123}_{DFG} in the main text (and in Equation 2 above) but here we drop the residue identities for simplicity because they do not affect the recursion.

Some example connected correlations are

$$g_1 = f_1 \tag{4}$$
$$g_{12} = f_{12} - g_1 g_2 \tag{5}$$
$$g_{123} = f_{123} - g_1 g_{12} - g_2 g_{13} - g_3 g_{12} - g_1 g_2 g_3 \tag{6}$$
$$g_{1234} = f_{1234} - g_1 g_{234} - g_2 g_{134} - g_3 g_{124} - g_4 g_{123} - g_{12} g_{34} - g_{13} g_{24} - g_{14} g_{23} - g_1 g_2 g_3 g_4 \tag{7}$$

which, carrying out the recursion, is equivalent to

$$g_1 = f_1 \tag{8}$$
$$g_{12} = f_{12} - f_1 f_2 \tag{9}$$
$$g_{123} = f_{123} - f_1 f_{23} - f_2 f_{13} - f_3 f_{12} + 2 f_1 f_2 f_3 \tag{10}$$

For a site-independent model, the connected correlations are all 0 except for the first order. This is why it is of interest to test the GPSM's ability to reconstruct the connected-correlations, as by definition a site-independent model that cannot capture covariation will fail to predict these values. In contrast, for our original r_{20} calculation even a site-independent model will be able to predict the word frequencies with limited accuracy, depending on the importance of correlations.

For each higher-order marginal f^{ijkl}_{abcd}, there is a corresponding connected correlation g^{ijkl}_{abcd}. To adapt the r_{20} score to use the connected correlations, we take the same sets-of-positions and the same top-20 words as we tested in our r_{20} evaluation, and transform the marginal for each word to the corresponding connected correlation. These words are those which appear frequently in the data, and therefore are more likely to be reliably estimated from a finite evaluation MSA, and so give lower estimation error. We then compute the Pearson r for these 20 values for the validation and target datasets, and average, as in the original r_{20} computation, giving the cc-r_{20} score.

In Fig. S7 we show the cc-r_{20} analysis for all GPSMs we tested using the kinase synthetic $1M$ dataset described in the main text. This plot is computed using the same MSAs as in Figure 3a in the main text, only the marginals used in the computation of r_{20} are converted to connected correlations, using the one-to-one mapping of marginals to connected-correlations described above, using which we compute cc-r_{20}. We find that the Mi3 model closely matches the Nat-target expectation.

We find the cc-r_{20} results shown in Fig. S7 are qualitatively similar to the r_{20} results in main text Figure 3a, and lead to the same conclusions: the Potts model matches the reference expected values well, while the VAE models are lower. As expected, the independent model gives an cc-r_{20} score of 0 at all orders. This test further demonstrates that both VAE models are able to capture some degree of correlated effects which the independent model cannot. It further demonstrates that even with this alternate measure of covariation, the Potts model is uniquely able to capture the higher-order effects in a way that closely matches the target. Additionally, this computation shows that even at second order ($n = 2$) the VAE predictions have error.

cc-r_{20} is more affected by estimation error (finite sampling error in the generative capacity metric) than r_{20}, so the statistics become difficult to estimate for higher orders of marginal. We find that even using validation MSAs of $6M$ sequences we can only reliably evaluate cc-r_{20} up to sixth order, in contrast to up to tenth order for the original r_{20} scores. While we could potentially go beyond sixth order by increasing the size of our test MSAs beyond $6M$, as discussed further below the necessary MSA sizes quickly diverge. For these reasons, r_{20} better highlights model accuracy to higher (tenth) marginal orders than the cc-r_{20}.

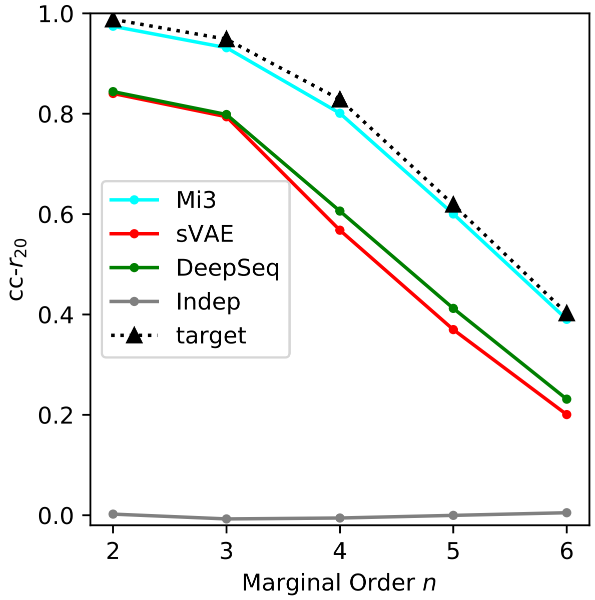

Figure S7. cc-r$_{20}$ computation for the Kinase protein family, using the MSAs for the synthetic $1M$ test in the main text. This plot is analogous to Figure 3a in the main text, and is computed using the same MSAs, only the r_{20} metric is replaced by the cc-r$_{20}$ metric.

Supplementary Note 8 - Analysis of r_{20} estimation error as a function of MSA depth

When computing the r_{20} scores we are able to quantify estimation error, as can be seen by the r_{20} upper limit (Fig. S6, b, black dotted line). Here we provide quantitative intuition for the behavior of the r_{20} upper limit as a function of the evaluation MSA size N, which explains the challenge of eliminating estimation error entirely.

Consider a particular set of positions for which we estimate the frequency f of each word at those positions in the target probability distribution, based on a finite MSA of size N generated from the target probability distribution, giving estimated marginals $\hat{f}$. We retain only the top twenty observed words for use in the r_{20} computation. The statistical variance in $\hat{f}$ caused by finite-sampling error will be $f(1-f)/N$, following a multinomial sampling process, and we will approximate that all top 20 marginals have similar magnitude and we approximate this error as $\langle f \rangle (1 - \langle f \rangle)/N$ for all twenty values, where $\langle f \rangle$ is the mean value of the top 20 marginals.

We can then approximate that the expected Pearson correlation ρ^2 between values estimated from two such MSAs will be $\rho^2 \approx \chi^2/(\chi^2 + \sigma^2)$ where χ^2 is the variance in the values of the top 20 marginals (reflecting the variance of the "signal"), and $\sigma^2 \approx \langle f \rangle (1 - \langle f \rangle)/N$ is the statistical error in each value (representing the variance of the "noise").

$\langle f \rangle$ and χ are properties of the protein family being modelled, at each position-set, and do not depend on N. This invariant allows us to extrapolate, since if we solve for $\langle f \rangle (1 - \langle f \rangle)/\chi^2 = N(1/\rho^2 - 1)$, the r.h.s. should be invariant when we change the size of the dataset MSA from N to N_0 or vice versa. If we estimate the r.h.s. for a particular N_0 and measured ρ_0 numerically, we can solve for ρ at higher N since $N(1/\rho^2 - 1) = N_0(1/\rho_0^2 - 1)$, or

$$N = N_0 \frac{\rho^2/(1-\rho^2)}{\rho_0^2/(1-\rho_0^2)}. \quad (11)$$

The approximations we used to derive this formula will become more accurate for larger N_0. We have tested this formula by predicting the expected r_{20} for MSAs of size N by extrapolating based on the measured r_{20} for MSAs of smaller size N_0, and find it is quite accurate.

This equation shows how extremely large MSAs can be required to reduce estimation errors when evaluating r_{20}, as the extrapolated N diverges as $\propto 1/x$ as $x = 1 - \rho^2$ approaches 0. For instance, if with an MSA of $6M$ sequences we obtain $r_{20} = 0.8$, then we would require $28.5M$ sequences to obtain $r_{20} = 0.95$ and $148M$ to reach $r_{20} = 0.99$.

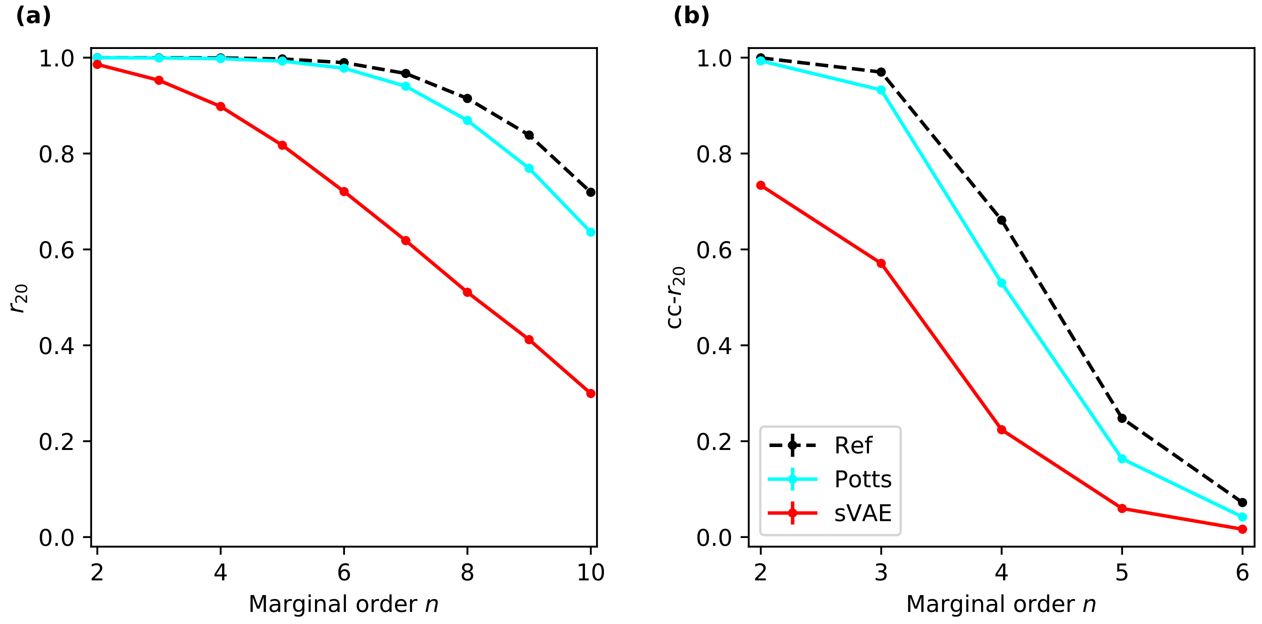

Figure S8. (a) r_{20} analysis and (b) $\mathrm{cc}\text{-}\mathrm{r}_{20}$ for the Potts+3 model of the RRM protein family, which includes a triplet interaction term. See text.

Supplementary Note 9 - Detecting higher-than-pairwise interaction terms using r_{20}

Here we demonstrate numerically that the r_{20} score is able to detect whether a GPSM can model datasets which require higher than second order interaction terms to model, even when such terms are sparse or rare. The tests we performed in the main text and above do not explicitly demonstrate this. In the main text, we performed synthetic tests in which the target probability distribution corresponds to a Potts model, which only requires up to pairwise interaction terms to model. Above, we also carried out synthetic tests in which the target probability distribution corresponds to an sVAE, which potentially requires higher-than-pairwise interaction terms to model, however this is not definitely proven. Therefore, we perform an additional test here in which we explicitly and artificially introduce a triplet interaction term into the target probability distribution function, and test the effect on our GPSM metrics for each GPSM.

In this test, we choose a single arbitrary set of three positions ijk in a natural sequence MSA for a protein family of interest, only conditioned to avoid choosing highly conserved positions. With this condition, the most common character at each of the three positions we chose in all our tests has roughly 10% to 20% frequency. Taking a Potts model parameterized based on the natural sequence MSA, we then artificially introduce a single triplet interaction parameter, J^{ijk}_{abc} for the characters abc corresponding to the most likely character at each of the three positions, creating a “Potts+3” model, from which we generate sequences. In other words, we add the value J^{ijk}_{abc} to the Potts energy function only for sequences with the triplet abc. We choose a value of $J^{ijk}_{abc} = -\log(2)$, since this will approximately double the frequency of the word “abc" assuming it is fairly rare. We then fit GPSMs to this Potts+3 biased MSA, and compute r_{20} for these new models relative to the target Potts+3 model, as well as $E(S)$ for a set of $100K$ sequences from the target MSA.

We performed this test for two protein families, RRM and response regulator, whose properties are explained in detail further below. In both cases, the introduction of this single triplet term causes a detectable decrease in the r_{20} score, particularly at order 10, as shown in Fig. S8 and Fig. S9. We also observe a decrease in $\mathrm{cc}\text{-}\mathrm{r}_{20}$. In comparison, if we perform the same test but without adding the triplet term, the Potts result matches the reference target, and we find no decrease in r_{20} at order 10 beyond the effects of finite sampling or estimation error (see Figures S11,S12 below).

This demonstrates that the r_{20} score can detect specification error due to lack of higher-order interaction terms, and further that it can do so even if the higher-order interaction terms are sparse, since we only added a single triplet term.

This test is artificial in the sense that we added a triplet interaction term without any biological justification.

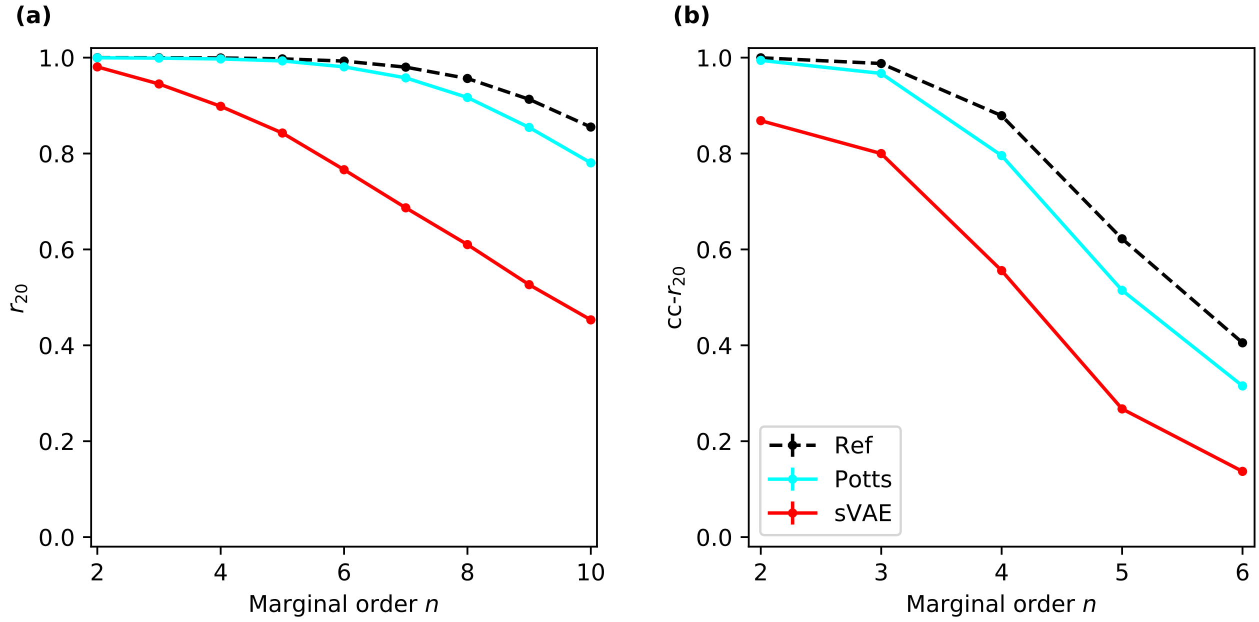

Figure S9. (a) r_{20} analysis and (b) cc-r_{20} for the Potts+3 model of the response regulator protein family, which includes a triplet interaction term. See text.

Because the existence of higher-order interaction terms in natural sequence probability distributions is still debated[17–19]), the strength and sparsity with which such interactions might appear is unclear. Nevertheless, our test provides a demonstration of a case in which r_{20} can be used to detect detect specification error even when higher-order interaction terms are sparse, and even for a single triplet interaction term as shown in this test. Additionally, for realistic MSA sizes available from Pfam, for up to $10K$ sequences as described further below, it is unlikely that properties of the natural sequence probability distribution can be fit better by including triplet terms, because finite size effects obscure the contributions of higher interaction terms, limiting our ability to reliably measure r_{20} to the higher orders at which the effects of the triplet term become apparent.

How is it that the r_{20} metric is able to detect the effect of a single triplet term, given that the r_{20} term is only based on a limited sample of sets-of-position for each word size n? That is, in our tests we only sample 3000 sets-of-positions for each word length n, which makes it unlikely that we sample the particular three positions ijk involved in the triplet interaction when computing r_{20}. More generally, how might the r_{20} score be used to detect the need for sparse higher-order interactions? We explain this through the effect chains and networks of epistatic interactions on the MSA marginals.

The Potts Hamiltonian is the easiest that can be used to interpret or explain this. The Potts model is parameterized by pairwise interaction terms J^{ij}_{ab} (couplings), and Potts models fit to protein MSAs have sparse couplings as we find many J^{ij}_{ab} are close to 0. However, it is important to distinguish couplings J^{ij}_{ab} (in the model parameters) from covariation (based on the MSA marginal statistics, such as connected-correlations). The Potts model usually generates higher than pairwise covariation despite only having pairwise couplings, as illustrated in section Supplementary Note 6 - above. Through long chains and networks of couplings, we find there is widespread covariation to varying degrees across the positions in the MSAs we studied. This means that introducing a new pairwise coupling to the model at positions i, j will affect the marginals at other positions k and will affect many higher-order marginals (word frequencies) throughout the MSA.

As an example of how r_{20} can be affected, consider the case where we introduce a single triplet term at positions ijk to an existing Potts model, as in the Potts+3 numerical test just described. If position i is strongly pairwise coupled in the Potts model to position x, and j to y, and k to z, then introducing a triplet term at ijk will also affect the marginals at positions xyz, generating higher-order covariations at xyz not well captured by a Potts model. Thus, even if the r_{20} calculation does not directly sample positions ijk where the interaction applies, it will likely sample many other sets of positions xyz whose marginal frequencies are affected by the triplet interaction at ijk. Our numerical test of r_{20} for the Potts+3 model supports this interpretation.

In this way, as long as there are enough chains or networks of interactions, even if the higher-order interactions

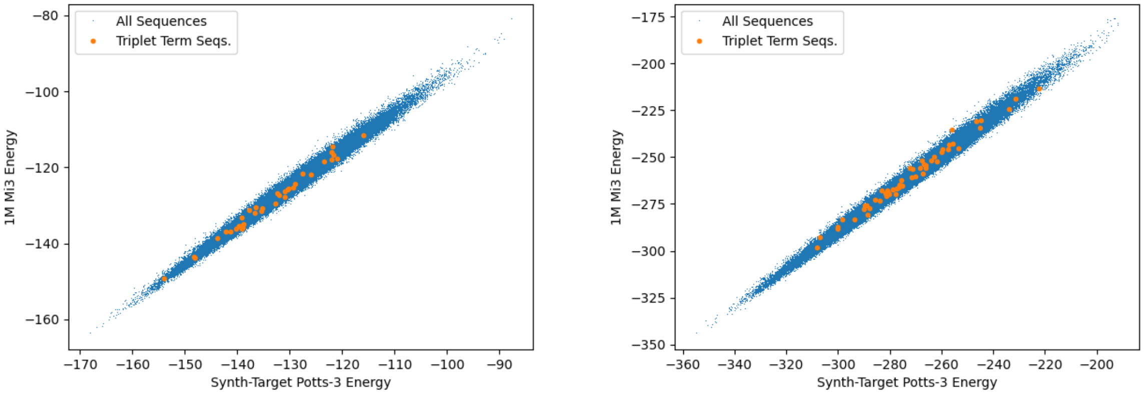

Figure S10. Statistical Energy correlations for the RRM protein family (left) and the response regulator protein family (right), when the target model is the Potts+3 model with a triplet interaction. Each point represents a sequence from the validation MSA. A random sample of about 50 sequences from the validation MSA which have the triplet interaction are shown in orange.

themselves are sparse, we expect that introducing a new model interaction at one set of positions will affect the marginals at many other sets of positions. This means that if a higher-order interaction term is necessary to model the MSA, the r_{20} score is likely to distinguish which GPSMs are well-specified for this MSA since many higher-order marginals throughout the MSA will be affected, for typical protein sequence data.

While low-frequency words could be informative, they cannot be individually, directly, or reliably measured. Nevertheless, the r_{20} score tests for them indirectly: if epistatic interactions increase the frequencies of low-frequency words, of which there are many, this necessarily reduces the frequency of the highest-frequency words since the frequencies must sum to 1. Thus, r_{20} indirectly measures the balance in probabilities between the low-frequency words and the individual highest 20 frequency words.

Supplementary Note 10 - Detecting higher-than-pairwise interaction terms using $E(S)$

We also consider the effect of the triplet term on the $E(S)$ metric for individual sequences. Here we repeat our procedure for the $E(S)$ correlation metric from the main text (Figure 5), but using the Potts+3 model.

To summarize this procedure, we score sequences of a target MSA generated from the Potts+3 model, both using the Potts+3 model $E(S)$ (including triplet term in the energy) and using the Potts model $E(S)$ inferred from the Potts+3 sequences, using the same models described above. The results using a target MSA of $100K$ sequences is shown in Fig. S10 for the RRM and response regulator protein families.

The effect of the triplet interaction term is not easily detectable from the $E(S)$ comparison: The magnitude of the triplet interaction in this case, which we fixed at $\log(2) = 0.7$ energy units, is smaller than the typical error of the reconstructed energy (the width of the blue distribution in the plot).

Our conclusion from this is that r_{20} is better suited to distinguish whether a model correctly accounts for the higher-order-terms needed to model a dataset. In the statistical energy plots, the effect of interaction terms on individual sequences is smaller than the total contributions from all the other interactions throughout the sequence. In contrast, r_{20} averages MSA statistics across sequences, and focuses on marginals, so is not limited in this way.

Supplementary Note 11 - Analysis of additional protein families

In addition to kinase (PF00069) used in the main text, we also performed our comparative GPSM analysis on three other protein families in order to demonstrate the generality and robustness of our results. These are RRM (PF00076), response regulator (PF00072), and ABC transporter (PF00005). These three other families all have a large number of available sequences, allowing us to perform the analysis with the same $10K$ training/validation MSA splits as for the kinase family. We intentionally selected them for their diverse properties, with different sequence lengths, levels of sequence conservation, and biological function. Our original kinase MSA has sequences of 232 residues after processing, whereas RRM has 48, response regulator has 107, and ABC transporter has 125 residues. The sequence length affects the balance of pairwise to single-site terms in the Potts models, since the number of

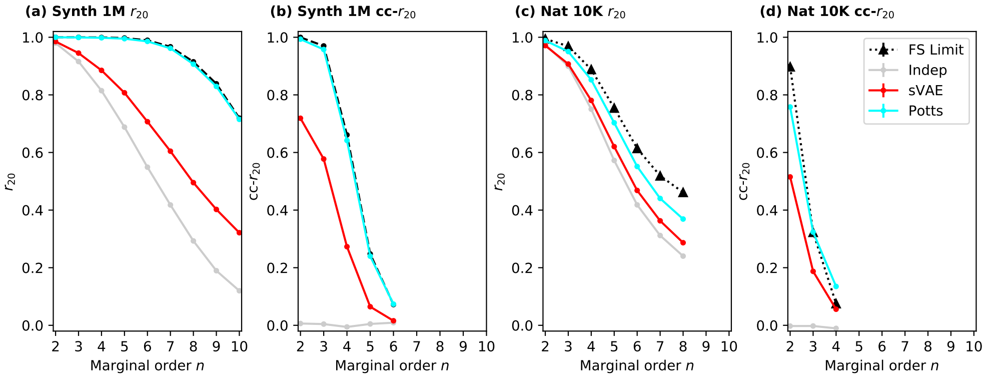

Figure S11. The RRM protein family (a) r_{20} analysis and (b) $\mathrm{cc\text{-}r_{20}}$ for a 1M synthetic test, (c) r_{20} analysis and (d) $\mathrm{cc\text{-}r_{20}}$ for the natural dataset. See text for description of the computation of the FS limit.

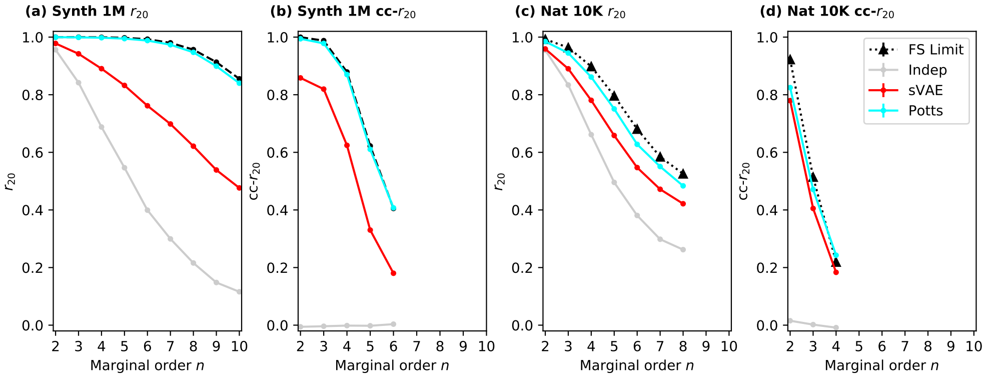

Figure S12. The response regulator protein family (a) r_{20} analysis and (b) $\mathrm{cc\text{-}r_{20}}$ for a 1M synthetic test, (c) r_{20} analysis and (d) $\mathrm{cc\text{-}r_{20}}$ for the natural dataset. See text for description of the computation of the FS limit.

single-site terms grows as L while the pair sites grow as L^2, so the amount and strength of higher-order covariation could differ among these families. Similar reasoning applies to even higher-order interactions.

In Figures S11,S12, and S13 we highlight the r_{20} results for these protein families for both the synthetic $1M$ test and the natural test, performed the same way as we did for kinase in the main text. That is, in the natural test, we train the models using $10K$ natural sequences obtained from Uniprot, using HHblits as described previously, and filtering so that no sequence more than 50% identity to any other. We then generate $6M$ sequence from each of these models, and compute r_{20} and $\mathrm{cc\text{-}r_{20}}$ by comparing the generated sequences to a target $10K$ MSA obtained from Uniprot, not overlapping with the training MSA. In the synthetic $1M$ test, we use a Potts model fit to $10K$ natural sequences as the target probability distribution, and then fit new models to $1M$ sequences generated from this target probability distribution. We then compute r_{20} and $\mathrm{cc\text{-}r_{20}}$ values comparing $6M$ sequences generated from each new model to $6M$ sequences generated from the target model. The relative r_{20} performance of the different GPSMs for all families are qualitatively similar to our results in the synthetic $1M$ kinase experiment (main text, Figure 3a), confirming our conclusions in the main text.

As noted in the main text, the Finite Sampling limit (FS limit) is meant to represent the expected error if the GPSM had modelled $p^0(S)$ exactly, which should be computed by computing r_{20} for two MSAs both drawn from $p^0(S)$. For the synthetic $1M$ test, we do this by comparing two MSAs drawn from $p^0(S)$ of $6M$ sequences each, mirroring the MSA sizes used to evaluate the GPSM r_{20} values. For the natural test, on the other hand, this is not possible because we are limited to $10K$ natural target sequences. As described in the main text, we therefore are only able to approximate the FS limit in the natural test by substituting the Potts $p_\theta(S)$, as an approximation to $p^0(S)$, and generate two MSAs of $10K$ and $6M$ sequences from this distribution, and compute r_{20} between them. This provides only an approximate estimate of the expected r_{20} if the GPSM had modelled $\hat{p}^0(S)$ exactly, since $\hat{p}^0(S)$ will differ from $p_\theta(S)$ when the training MSA is only $10K$ sequences. Therefore the FS limit for the natural test can only give an approximate estimate of the expected FS limit, which is why we annotate it with triangles in the plot to distinguish

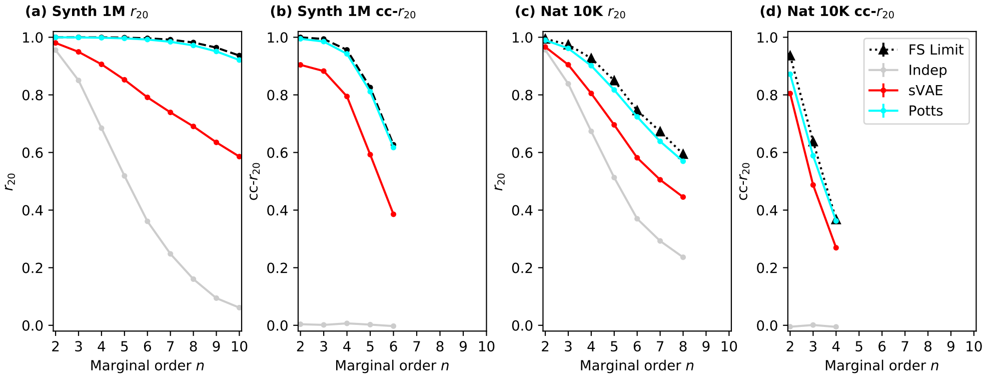

Figure S13. The ABC transporter protein family (a) r_{20} analysis and (b) cc-r_{20} for a 1M synthetic test, (c) r_{20} analysis and (d) cc-r_{20} for the natural dataset. See text for description of the computation of the FS limit.

from the more accurate result in the synth $1M$ test. Nevertheless, we observe that the Mi3 r_{20} scores are very close to this estimate, which is consistent with the hypothesis that the Potts model is well-specified to the natural protein sequence dataset, and the small difference can arise due to the approximation in the FS upper-bound.

Supplementary Note 12 - Typical natural sequence dataset MSA size

The $10K$ sequence training datasets we use in the main text are meant to illustrate performance for typical protein family dataset sizes. The size of $10K$ sequences is the number of estimated effective sequences N_{eff} remaining after curation and phylogenetic filtering for the twentieth most frequent protein (Cadherin) in Pfam (Fig. S14, right)[21]. Some of our measurements show significant out-of-sample error for Mi3 and the VAEs based on training sample size alone, suggesting that the vast majority of GPSMs training on natural data could be subject to the level of out-of-sample error reported in our results.

In Pfams's Top 20 most frequent protein domains, ranked by total number of sequences, there are between 10^5 and 10^6 total sequences each (Fig.S14, right). In this work, we use the fourth most frequent protein out of this ranking, kinase (PF00069), in the main text. In Supplementary Note 11, we present results for three other proteins in the Pfam Top 20: RRM (PF00076), response regulator (PF00072), and ABC transporter (PF00005).

After curation and phylogenetic filtering of the kinase MSA from Pfam, we retained only $N_{\text{eff}} \sim 22K$, or ∼5% of the original $\sim 424K$ kinase sequences (Fig. S14, left). Extending this fraction of $\sim 5\%$ to the other Top 20 proteins, we estimate that N_{eff} is capped at $\sim 10^5$ $(100K)$ for GPSMs trained on single domains, and that proteins outside the Top 20 can generally expect $N_{\text{eff}} < 10^4$ $(10K)$ after similar processing. This tabulation of Pfam data demonstrates that, for the vast majority of proteins with publicly available natural sequence data, contemporaneous GPSMs must have approximately $N_{\text{eff}} < 10K$ for training, validation, and testing.

Supplementary Note 13 - MSA Transformer

Transformer-based models are currently of great interest in protein sequence modeling[22–27]. Here we test a transformer protein sequence model on the RRM dataset, the MSA transformer[27], using the r_{20} metric. MSA Transformer has publicly-accessible code and a pretrained model, which we used to generate synthetic protein sequences using a modified technique detailed below. This model is composed of a large neural network with discrete variables that can model a multinomial distribution through increasingly complex transformations of hidden representations.

Although here we test it using our GPSM metrics, the MSA transformer model is significantly different enough in goals and target dataset from the GPSMs we have studied in the main text, such that this model and our other GPSMs are not strictly comparable, as we describe next. Nevertheless, there are many similarities as well, and since it is possible to generate sequences from this model, we take the opportunity here to evaluate it using our metrics.

Firstly, the original MSA Transformer work does not discuss the possibility or method of generating de novo protein sequences[27]. However, the model has generative capacity because it belongs to the category of autoregressive models which can generate protein sequences through iterative inference[28]. We therefore use the publicly-

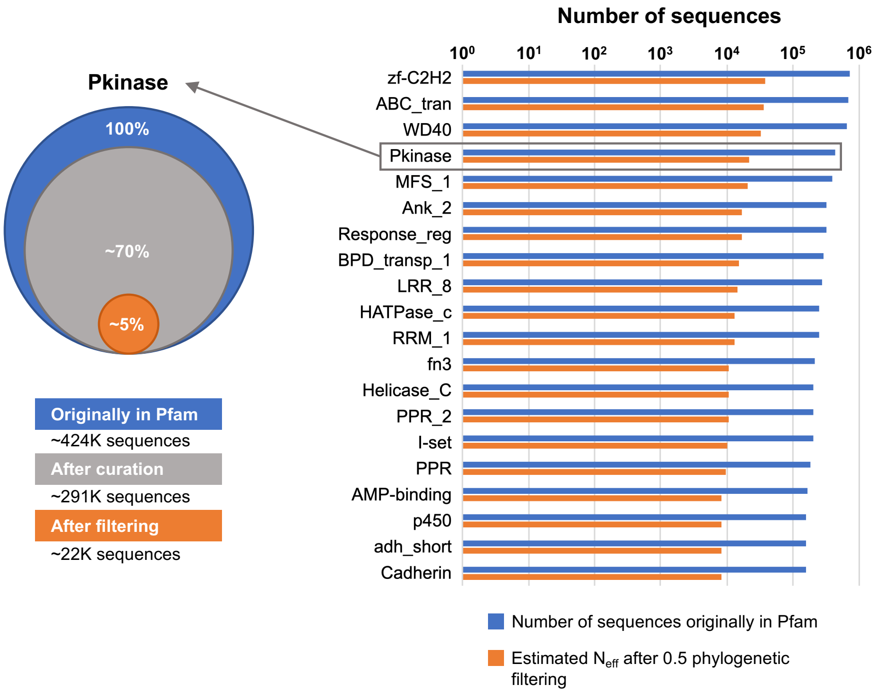

Figure S14. Pfam Top 20**.** GPSMs trained on publicly available natural sequence data could be inherently data-starved. **Right** Log-scaled histogram of Pfam sequence frequencies. Sorted by the log-scaled number of sequences originally in Pfam (blue), the histogram shows estimated number of effective sequences N_{eff} after phylogenetic filtering at the 0.5 similarity cutoff (orange). All estimates are based on the actual N_{eff} for Pkinase, the fourth most frequent protein family and the one used in this work, which is $\sim 22K$ sequences, or ~5% of the total $\sim 424K$ Pkinase sequences in Pfam (left). Cadherin, the last entry (bottom), has $N_{\text{eff}} < 10^4$ ($10K$ sequences), meaning that this must be the approximate upper-bound of N_{eff} for GPSMs training on natural data outside the Pfam Top 20. Since all proteins outside the Pfam Top 20 must $N_{\text{eff}} < 10^4$, we chose $10K$ sequences as the lower limit of total training sequences for our synthetic analysis. **Left** Curation and phylogenetic filtering breakdown for Pfam Pkinase dataset. Of $\sim 424K$ Pkinase sequences in Pfam (blue), only $\sim 291K$ ($\sim 70\%$) remained after curation (grey). This curated set was phylogenetically filtered at 0.5 similarity, resulting in $N_{\text{eff}} \sim 22K$ (orange), or 5% of the original $\sim 424K$.

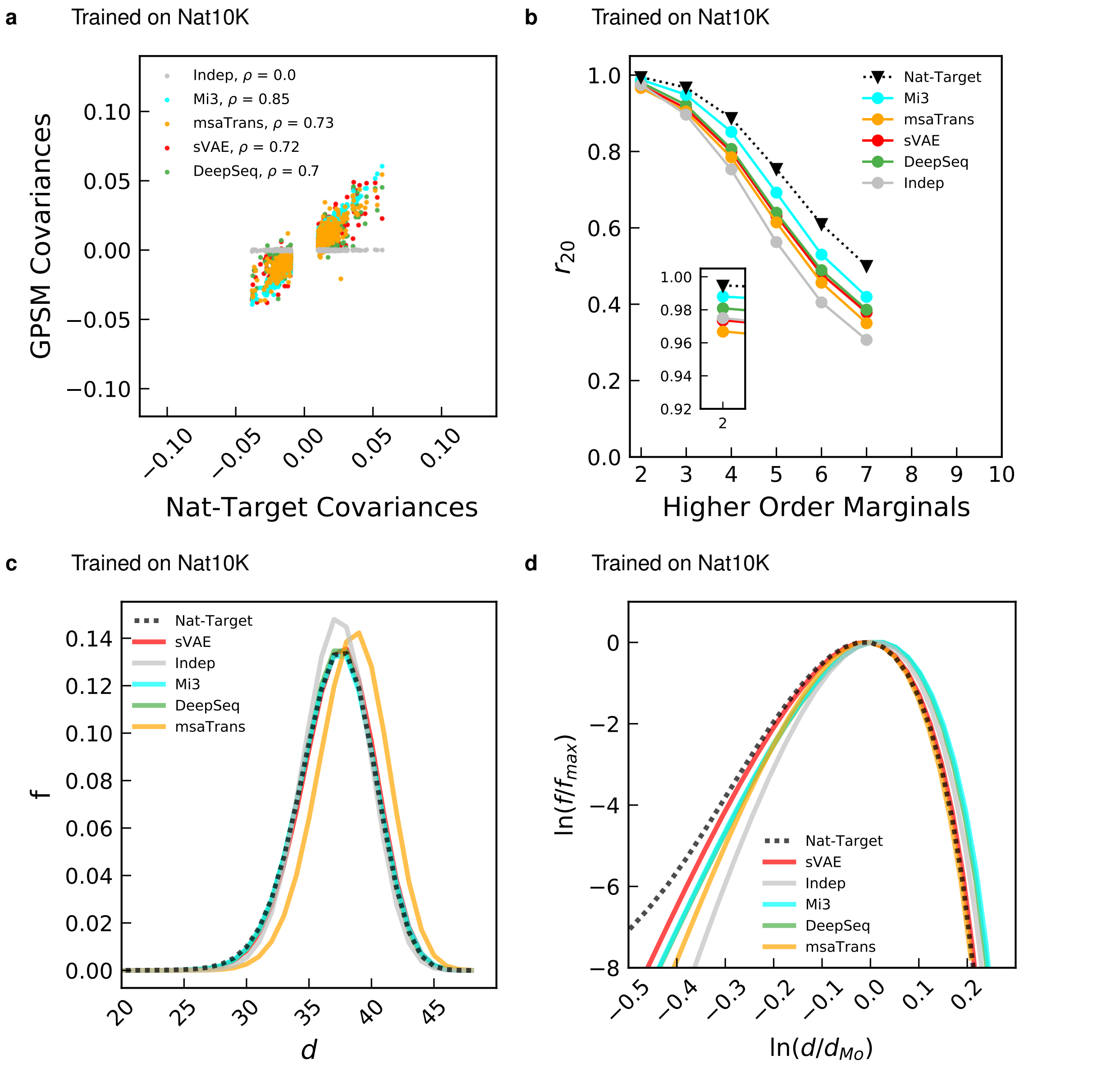

Figure S15. MSA Transformer. MSA Transformer (msaTrans, orange) was pre-trained in a starkly different fashion from the other models used in this work. Based on the same natural $10K$ RRM sequences used to train the other models in the Natural Analysis, MSA Transformer was then used to generate a synthetic evaluation MSA of size $1M$ for the RRM protein in an autoregressive process. This $1M$ evaluation MSA was then used in the following four generative capacity measurements as was done in the Natural Analysis in ms. Other than the inclusion of MSA Transformer, these measurements were performed exactly as in the Natural Analysis for RRM, with the exception of r_{20} as detailed below. Most notably, MSA Transformer's generative capacity is qualitatively similar to the VAEs on our most discriminative metric, r_{20}, indicating that MSA Transformer captures about as much covariation as the VAEs. **a** Pairwise covariance correlations. This measurement was performed using a $500K$ evaluation MSA for all models, including MSA Transformer, as in Figure 2, ms. **b** r_{20}. This measurement was performed using a $1M$ evaluation MSA for MSA Transformer, and a $6M$ evaluation for all other models, as in Figure 3, ms. The r_{20} calculation was repeated using only $200K$ sequences with qualitatively similar results as $1M$, so we do not expect performance to increase if more sequences were used. $1M$ was selected for MSA Transformer due to exceedingly long sequence generation time. **c** Hamming distance distribution. This measurement was performed using a $50K$ evaluation MSA for all models, including MSA Transformer, as in Figure 4, ms. **d** Log-log Hamming distance distribution. This measurement was performed using a $50K$ evaluation MSA for all models, including MSA Transformer, as in Figure 4, ms.

available pretrained model for MSA Transformer, but we use an adapted sequence generative process akin to other autoregressive models[28,29]. In this method, starting from a given seed MSA, we mask out an entire protein sequence, and use the MSA transformer model to predict a probability distribution $p(x_i|MSA)$ over the residues x_i at a masked position i, given the known residues in the remainder of the seed MSA. We replace one mask token by sampling a new residue from $p(x_i|MSA)$. This process is repeated for each residue in the missing sequence until all positions are filled in. We follow an "easy first" approach, where we first sample from the most conserved position, ordered by their site entropy $s_i = -\sum_a f_a^i \log f_a^i$. In our test, we use groups of 256 sequences randomly sampled from the $10K$ natural RRM training MSA used in the main text, as seed MSAs.

Second, MSA Transformer learns from $26M$ MSAs based on each sequence in Uniprot50, creating MSAs with average depth of 1192. Each of these MSAs were phylogenetically filtered down to the most diverse 256 sequences. In contrast, for the Natural Analysis, the GPSMs in this work were trained on only one specific protein family of size $10K$ after phylogenetic filtering. In other words, the target distribution $p^0(S)$ for the GPSMs we examine in ms corresponds to the probability of sequences in a particular protein family, while for MSA Transformer the equivalent probability distribution contains information about other protein families as well. Additionally, the sequences generated using the adapted sequence generative process for MSA Transformer depends on the seed sequences, as it generates sequences from the same protein family as the seed, which means that the generated sequences are not strictly i.i.d. samples from the model distribution $p_\theta(S)$ over all protein families. Because of these differences we expect the inferred residue frequencies to be slightly different compared to a model that was trained for only one specific family. Additionally the MSA pre-processing is performed differently for the MSA Transformer, which again will affect the residue frequencies.

Third, the sequence generation approach for MSA Transformer is very slow, because it needs L calls to the model, where the model complexity is $\mathcal{O}(L^2M) + \mathcal{O}(LM^2)$, for a total computational cost of $\mathcal{O}(L^3M) + \mathcal{O}(L^2M^2)$. To speed up the generation process we mask out 32 of the 256 sequences to generate more than one protein at a time.

Fourth, MSA Transformer was trained with input noise to avoid over-fitting, which might have some undesired effects on the predicted output probabilities. This plays a similar role to the regularization or pseudocounts used to infer GPSM models.

Despite these differences, we generated an evaluation MSA of $1M$ synthetic sequences from MSA Transformer for RRM using the iterative sequence generation method described above. The results in Figure S15 show that the generative capacity of MSA transformer does not quite reach the performance of the models trained on a single MSA, but is comparable to the performance of state-of-the-art VAEs. In general, it is encouraging that MSA Transformer can capture covariation at about the level of VAEs, and above that of the Indep model. However, the method scales very poorly and it might take an unreasonable amount of time to generate longer proteins, such as kinase. In this test, the target MSA is a natural sequence protein MSA limited to $10K$ sequences, as all models were fit to natural protein sequence data. As noted in the main text, the fact that only $10K$ sequences are available for the natural target MSA causes the r_{20} metric comparison to be obscured by high estimation error, as in main text Figure 3d. This means the $r20$ values for different models appear more similar to each other than if a similar test was performed using a large synthetic target MSA, however this is unavailable.

References

1. Charte, D., Charte, F., García, S., del Jesus, M. J. & Herrera, F. A practical tutorial on autoencoders for nonlinear feature fusion: Taxonomy, models, software and guidelines. *Information Fusion* **44**, 78–96 (2018).

2. Ding, Z. *et al.* Guided Variational Autoencoder for Disentanglement Learning. In *2020 IEEE/CVF Conference on Computer Vision and Pattern Recognition (CVPR)*, 7917–7926 (2020). ISSN: 2575-7075.

3. Kingma, D. P. & Welling, M. Auto-Encoding Variational Bayes. *arXiv:1312.6114 [cs, stat]* (2014). URL http://arxiv.org/abs/1312.6114. ArXiv: 1312.6114.

4. Sinai, S., Kelsic, E., Church, G. M. & Nowak, M. A. Variational auto-encoding of protein sequences. *NeurIPS 2017 MLCB workshop* (2017). ArXiv: 1712.03346.

5. Chollet, F. *et al.* Keras (2015). URL https://github.com/keras-team/keras. Original-date: 2015-03-28T00:35:42Z.

6. Srivastava, N., Hinton, G., Krizhevsky, A., Sutskever, I. & Salakhutdinov, R. Dropout: a simple way to prevent neural networks from overfitting. *The Journal of Machine Learning Research* **15**, 1929–1958 (2014).

7. Ioffe, S. & Szegedy, C. Batch Normalization: Accelerating Deep Network Training by Reducing Internal Covariate Shift. In *International Conference on Machine Learning*, 448–456 (PMLR, 2015). URL http://proceedings.mlr.press/v37/ioffe15.html. ISSN: 1938-7228.

8. Kingma, D. P. & Ba, J. Adam: A Method for Stochastic Optimization. *arXiv:1412.6980 [cs]* (2017). URL http://arxiv.org/abs/1412.6980. ArXiv: 1412.6980.

9. Rezende, D. J., Mohamed, S. & Wierstra, D. Stochastic Backpropagation and Approximate Inference in Deep Generative Models. In *International Conference on Machine Learning*, 1278–1286 (PMLR, 2014). URL http://proceedings.mlr.press/v32/rezende14.html. ISSN: 1938-7228.

10. Kingma, D. P. & Welling, M. An introduction to variational autoencoders. *Foundations and Trends® in Machine Learning* **12**, 307–392 (2019). URL http://dx.doi.org/10.1561/2200000056.

11. Ding, X., Zou, Z. & Brooks Iii, C. L. Deciphering protein evolution and fitness landscapes with latent space models. *Nature Communications* **10**, 5644 (2019). Number: 1 Publisher: Nature Publishing Group.

12. Riesselman, A. J., Ingraham, J. B. & Marks, D. S. Deep generative models of genetic variation capture the effects of mutations. *Nature Methods* **15**, 816–822 (2018).

13. Lucas, J., Tucker, G., Grosse, R. & Norouzi, M. Understanding posterior collapse in generative latent variable models. *ICLR 2019 Workshop DeepGenStruct* (2019).

14. Dai, B., Wang, Z. & Wipf, D. The usual suspects? Reassessing blame for VAE posterior collapse. In III, H. D. & Singh, A. (eds.) *Proceedings of the 37th International Conference on Machine Learning*, vol. 119 of *Proceedings of Machine Learning Research*, 2313–2322 (PMLR, 2020). URL http://proceedings.mlr.press/v119/dai20c.html.

15. Granata, D. & Carnevale, V. Accurate Estimation of the Intrinsic Dimension Using Graph Distances: Unraveling the Geometric Complexity of Datasets. *Scientific Reports* **6**, 31377 (2016).

16. Haldane, A. & Levy, R. M. Influence of multiple-sequence-alignment depth on potts statistical models of protein covariation. *PRE* **99**, 032405 (2019).

17. Schneidman, E., Still, S., Berry, M. J. & Bialek, W. Network information and connected correlations. *Phys. Rev. Lett.* **91**, 238701 (2003). URL https://link.aps.org/doi/10.1103/PhysRevLett.91.238701.

18. Schneidman, E., Berry, M. J., Segev, R. & Bialek, W. Weak pairwise correlations imply strongly correlated network states in a neural population. *Nature* **440**, 1007–1012 (2006).

19. Merchan, L. & Nemenman, I. On the sufficiency of pairwise interactions in maximum entropy models of networks. *Journal of Statistical Physics* **162**, 1294–1308 (2016).

20. Percus, J. The equilibrium theory of classical fluids. *by HL Frisch and JL Lebowitz, Benjamin, New York* (1964).

21. El-Gebali, S. *et al.* The Pfam protein families database in 2019. *Nucleic Acids Research* **47**, D427–D432 (2019). Publisher: Oxford Academic.

22. Rives, A. *et al.* Biological structure and function emerge from scaling unsupervised learning to 250 million protein sequences. *Proceedings of the National Academy of Sciences* **118** (2021). URL https://www.pnas.org/content/118/15/e2016239118. Publisher: National Academy of Sciences Section: Biological Sciences.

23. Madani, A. *et al.* ProGen: Language Modeling for Protein Generation. preprint, Synthetic Biology (2020). URL http://biorxiv.org/lookup/doi/10.1101/2020.03.07.982272.

24. Vig, J. *et al.* BERTology Meets Biology: Interpreting Attention in Protein Language Models. *bioRxiv* 2020.06.26.174417 (2020). Publisher: Cold Spring Harbor Laboratory Section: New Results.

25. Elnaggar, A. *et al.* ProtTrans: Towards Cracking the Language of Life's Code Through Self-Supervised Deep Learning and High Performance Computing. *bioRxiv* 2020.07.12.199554 (2020). Publisher: Cold Spring Harbor Laboratory Section: New Results.

26. Choromanski, K. M. *et al.* Rethinking Attention with Performers. In *accepted to ICLR 2021 (oral presentation)* (2021). URL https://arxiv.org/abs/2009.14794.

27. Rao, R. *et al.* MSA Transformer. *bioRxiv* 2021.02.12.430858 (2021). URL https://www.biorxiv.org/content/10.1101/2021.02.12.430858v1. Publisher: Cold Spring Harbor Laboratory Section: New Results.

28. Trinquier, J., Uguzzoni, G., Pagnani, A., Zamponi, F. & Weigt, M. Efficient generative modeling of protein sequences using simple autoregressive models. *arXiv:2103.03292 [cond-mat, q-bio]* (2021). URL `http://arxiv.org/abs/2103.03292`. ArXiv: 2103.03292.

29. Strokach, A., Becerra, D., Corbi-Verge, C., Perez-Riba, A. & Kim, P. M. Fast and flexible protein design using deep graph neural networks. *Cell Systems* **11**, 402–411.e4 (2020). URL `https://www.sciencedirect.com/science/article/pii/S2405471220303276`.
