# Supplementary material for: The generative capacity of probabilistic protein sequence models
Source: Nat Commun. 2021 Nov 2;12:6302. doi: 10.1038/s41467-021-26529-9 (PMC8563988; doi:10.1038/s41467-021-26529-9)
Supplement: Supplementary file 2 — Peer Review File [file 41467_2021_26529_MOESM2_ESM.pdf]

nature portfolio

Peer Review File

Generative Capacity of Probabilistic Protein Sequence Models

Open Access This file is licensed under a Creative Commons Attribution 4.0 International License, which permits use, sharing, adaptation, distribution and reproduction in any medium or format, as long as you give appropriate credit to the original author(s) and the source, provide a link to the Creative Commons license, and indicate if changes were made. In the cases where the authors are anonymous, such as is the case for the reports of anonymous peer reviewers, author attribution should be to 'Anonymous Referee' followed by a clear attribution to the source work. The images or other third party material in this file are included in the article's Creative Commons license, unless indicated otherwise in a credit line to the material. If material is not included in the article's Creative Commons license and your intended use is not permitted by statutory regulation or exceeds the permitted use, you will need to obtain permission directly from the copyright holder. To view a copy of this license, visit http://creativecommons.org/licenses/by/4.0/.

Reviewers' Comments:

Reviewer #1:
Remarks to the Author:
Generative statistical modeling of protein sequences is arguably an important emerging subject in computational biology. The manuscript "Generative Capacity of Probabilistic Protein Sequence Models" addressed a number of highly relevant questions in this context: (i) Given two model architectures, like Potts models or VAE as discussed in the ms, can we design systematic measures to compare the accuracy of the models as generative models? (ii) Having such metrics in our hands, what model architecture is the most adapted for protein sequences?

The model reports interesting progress on both questions. It proposes the so far probably most systematic way to take higher-order statistical features into account in comparing test data with synthetic data coming from a generative model. And it results in far-reaching conjectures about two of the generative models used in the protein literatures (Potts, VAE). However, important questions remain open. First of all, the ms is written in a very technical style mostly accessible to specialists in the field. This would make it more suitable to a more specialized journal than Nature Comm., I would urge the authors to work on their presentation to make it more accessible to a broader audience. I think that both questions are relevant to a broader audience, also beyond the application to protein sequences, and this audience should be addressed rather than the very specialists of the very specific question.

Besides this general remark, there are a number of major points to be addressed by the authors:

1) One of the major technical novelties of the paper is the measure r_20 (Maybe I missed something but I did not understand the meaning of this name), which systematically compares higher-order statistics in "real" test data and sequences generated from the models. There are a few remarks and questions concerning this measure:

(a) The authors insist that it takes into account higher-order statistics, saying that the existing literature hardly goes beyond one- and two-site terms. This is not entirely true, several papers consider higher order terms like three-point correlations or distributions of sequences when projected onto dimensionally reduced spaces like done in RBM, Hopfield models, VAE etc etc. showing non-trivial moments beyond the first two. In this sense, the authors overclaim the novelty of the approach, even if r_20 is more systematic in its construction than any of the published examples I know.

(b) The construction of r_20 consider high-frequency n-tuples for n<=10. I would expect the highest frequencies to be related to combinations of conserved amino acids, so at least part of the signal is explainable by the single-site statistics (explaining why also the profile model has quite a good performance for smaller values of n). Instead of frequencies, higher-order statistics can be better detected by connected correlations or cumulants. I also would expect that low-frequency words may be informative (if expected at higher frequencies based on lower-order information), but possibly cannot ne detected reliably having limited statistics for larger values of n. So would it be possible to base r_20 rather on connected correlations than on frequencies?

(c) For each n, 3000 combinations of n sites are randomly selected. For larger n, this leads to a very very sparse sampling of all combinations of sites (e.g. for proteins of 200 sites and n=10, there are ~2e+16 choices, and only 3000 are considered). Most combinations of multiple sites show very small correlations (e.g. for n=2 those in the gap in Fig 2a-c), while the large correlations form a very sparse network (e.g. points beyond +-0.05 in these plots). So they are hardly ever taken into account in r_20. Shortly, if relevant higher-order terms are sparse, they will not be seen by the random selection of sites in r_20, and not be taken into account in comparing test data and synthetic data.

2) The ms provides analyses on synthetic data coming from a pairwise Potts model. The authors admit

that this choice favors the Potts model also in model learning, since the exact model can be learned (and will be learned by maximum likelihood in the limit of infinite data). However, it would be important to see how the measures perform, when systematic higher-order terms are introduced into the model. Does r_20 work, e.g., perfectly up to some k-1 for Mi3, when there are fields, pairwise interactions and true k-body interactions exist in the data-generating "true" model (I mean interactions not decomposable into lower-order interactions, like k-spin interactions J s1 s2 ... sk in the Ising model)? This would be important to make deviations in r_20 from the optimal perfomance better interpretable and thus usable for the systematic construction of better models.

3) Concerning still the synthetic data, only dataset of 10K and 1M sequences were used. Many protein families are smaller, so it would be interesting to see results for smaller dataset. Does, e.g., the simpler independent model win for small datasets? Furthermore, it is said that for 1M the out-of-sample error is negligible for VAE, but these are later said to have 2.7M parameters. I would naively expect the out-of-sample error to vanish when the sample size is far bigger than the number of parameters.

4) Concerning the analysis for real proteins, only a single family is analyzed, but far reaching conclusions are drawn for proteins in general. Pfam contains almost 20 000 protein families, with downloadable MSA reaching from very few (<10 sequences) to almost 1M sequences. I think it would be easy to assess the robustness of the claims for substantially larger datasets of variable MSA depth.

5) I did not find anything about code availability. It would be great to have a code implementing the different measures, given to MSA as input (one the target MSA, one the synthetic one).

Minor remarks (in order of appearance):

i) A protein of length 100 would have about 1B parameters and not 10B.

ii) The last two paragraphs on page 2 are an example of the extremely technical, hard-to-read style of the paper.

iii) The likelihood in Sec' "Target distributions" should either be given as the product of probabilities of sum of log-probabilities.

iv) Fig. 1 is a bit complicated, but I have to admit that I have no good suggestion to simplify it. Maybe the authors have one?

v) Sec. "Pairwise covariance correlations": individual C^ij_ab may be zero, but this is no indication of a lack of co-variation. Typically a scalar correlation measure taking all combinations of amino acids has to be taken (l1 or l2 norm, mutual information).

vi) Covariances close to zero in Fig. 2 are omitted. Are they included in the determination of the reported correlations \rho?

vii) In Fig. 2d-f, the r_20 Nat-Target decays much faster in the natural data than in the synthetic data. Do the authors have any explanation why?

viii) Concerning Hamming distances, not only Hamming distances between data of the same type are interesting, but also inter-dataset distances. For illustration, imagine that data are sampled from a distribution P(S). In the first case, the target is sampled, and the generated is just a copy of it. So the distance distributions of the two are the same, but the inter-distances have a peak in zero. If the two sets are independently sampled, the inter-distribution will also be equivalent to the intra-distributions. And last but not least, if the two are sampled independently, and in the generated dataset all positions are globally permuted, the intra-distributions will be equivalent, but the inter-distributions larger.

ix) Concerning the statistical energy correlations, the data-generating model has to be known. In this case, it would be interesting to consider models with higher-order couplings, which cannot be reproduced fully by the Potts model, as mentioned before.

x) Note that E = -log P cannot be calculated analytically for Mi3, only up to a constant (the free energy).

xi) The Methods section lack details about the VAE structure. How are hyperparameters selected (like the number and size of layers), and the dimension of latent space? How do the authors exclude, that their results about the limited generative capacity of VAE is not just a result of not optimal choices? I am astonished by only 7 dimensions in latent space.

To conclude, I think that the ms addresses an interesting question and shows interesting evidences, but should be complemented by further analysis to fully support the author's claims.

Reviewer #2:
Remarks to the Author:
Review of "Generative Capacity of Probabilistic Protein Sequence Models" by McGee, Novinger, Levy, Carnevale, and Haldane.

The authors present a systematic comparison of classic ("shallow") and modern ("deep") models for learning epistatic interactions in via parametric probability distributions of protein sequences given a fixed alignment frame. (High-order) epistatic interactions means the co-evolution of two (or more) sites in a protein sequence, meaning, that they vary in the same way between different homologous protein sequences. This exercise is useful since understanding these interactions can be important to do protein engineering, protein structure prediction, prediction of the impact of genetic variations on protein functon(and potentially disease), or to do protein design. There is and has been extensive activities in this area of research, and interest has surged in around 2013 when several groups independently found that Potts models were very good at capturing pair-wise epistatic interactions. With the mainstreaming of deep learning methods deep generative models have seen several applications as they promise to capture more complicated multi-site epistatic interactions due to the superior representative power. The authors aim to test whether this promise holds.

The somewhat surprising result the authors present is that the simple Potts model outperforms the more sophisticated deep models when it comes to faithfully reproducing high-order epistatic interactions.

The authors use natural and synthetic data-set, which represent a protein-family aligned by multiple sequence alignment (MSA). The authors compare two "shallow" models: independent sites (no interaction between sites in MSAs) and a Potts model (adding pair-wise interactions to the independent sites model), and two "deep" models corresponding to two different variational auto-encoder models. The authors use four different assessment criteria to see whether the epistatic interactions in the data are faithfully captured by the learned models. Using samples generated from the learned model they compare: Pair-wise covariance, higher order marginal statistics, Hamming distance distributions, and statistical energies, between model samples and "true" reference data. The latter test is only done for synthetic data since statistical energies are unknown in true data-sets.

Overall, I like the study. However, it is highly technical and many of the potential explanations used to rationalize the findings are well-known, and are linked connected to inappropriate use of deep learning methods outside the domain of applicability -- using continuous variable models to learn multinomial data -- which brings both statistical sub-optimality and topological problems. Other problems are

related to optimization and learning in these models. These sub-optimal assumptions are not made on the shallow models, although they could have been made to make the comparison fairer -- but also pointless. Nevertheless, I was disappointed that no effort was made to try to side-step these severe limitations. For instance, using a based transformer models could have resulted in a more level-headed comparison and within right application domain: sequence modeling. There are MSA-based transformer models described in the literature (https://www.biorxiv.org/content/10.1101/2021.02.12.430858v1.full). Including such a model in the evaluation would eliminate some of the known limitations of variational auto-encoder based models of sequence data from the equation and more fairly compare deep versus shallow models.

Some general comments:
- Figures are nicely done, however, axis-labeling and captioning could be made clearer. It is not always obvious what is being compared in the plots (in particular fig 2) what synthetic model is used where?
- The higher order marginal statistics calculation is poorly explained in the main-text. It is not clear to this reader why there should be any correlations for these quantities with the data in the independent sites model.
- One of the few examples of Potts models actually generating experimentally validated protein sequences and the statistical energy correlating with stability and melting temperature should be cited: https://onlinelibrary.wiley.com/doi/abs/10.1002/anie.201713220
- The Monte Carlo estimator the authors use to compute the statistical energy (marginal likelihood of the VAE) is known to converge slowly in some cases. 1000 could be on the low end. The authors should include a plot of the boot-strapped error of this quantity for a few representative sequence as function of the number of samples in the SI.

Response to Reviewers

We thank both reviewers for their insightful suggestions and comments, which we feel have helped improve the manuscript.

The summary of our response and changes are as follows:

We performed a number of new calculations in response to the reviewers' comments, each of which took significant computational and development time, which are:

1. Repeating our entire analysis on three new protein families, to show that our results are general and not specific to the kinase protein family.
2. Computing the cc-r20 (or "connected-correlation r20") score for all our models, which is based on correlation values rather than frequencies as in the original r20 score. This metric is much more affected by finite-sampling error than r20, but evaluates in a hierarchical way the amount of correlation at any order that cannot be accounted for by lower orders.
3. Performed a series of tests in which we artificially add k-body interaction terms (k=3) to a target Potts model and test whether the r20 metric can detect model misspecification for GPSMs fit to this target. Our results demonstrate that the r20 metric can detect model misspecification involving higher-order terms, and does so even if the higher-order interaction terms are rare or sparse.
4. Developed and tested a method to generate sequences from "MSA transformer".

In the main text/manuscript (ms) we have included a new figure (Figure 3a, ms) to better explain the r20 metric, and have replaced Figure 1, ms, with a new figure to better describe our overall procedure. We also significantly modified the introduction to better put our work in a more general context. Finally, to answer the reviewer's multiple questions about the r20 score, we also significantly expanded our discussion of which types of model misspecification it can detect.

In order to address reviewer questions related to Transformer models and comparisons to Natural Language Processing literature, we asked for assistance from two Machine Learning researchers with expertise in these areas, Sandro Hauri and Slobodan Vucetic. Based on their contributions, we feel it is appropriate to include them as additional authors. They have performed new computations using Transformers now included in the ms and Supplementary Information (SI), and contributed significantly to discussion and editing of the updated manuscript.

Our detailed responses are in non-italic dark text below, and the updates to the manuscript and Supplementary Information are highlighted in red in those documents.

Reviewer 1 (Expertise: generative modeling for protein design):

Generative statistical modeling of protein sequences is arguably an important emerging subject in computational biology. The manuscript "Generative Capacity of Probabilistic Protein Sequence Models" addressed a number of highly relevant questions in this context: (i) Given two model architectures, like Potts models or VAE as discussed in the ms, can we design systematic measures to compare the accuracy of the models as generative models? (ii) Having such metrics in our hands, what model architecture is the most adapted for protein sequences?

The model reports interesting progress on both questions. It proposes the so far probably most systematic way to take higher-order statistical features into account in comparing test data with synthetic data coming from a generative model. And it results in far-reaching conjectures about two of the generative models used in the protein literatures (Potts, VAE). However, important questions remain open. First of all, the ms is written in a very technical style mostly accessible to specialists in the field. This would make it more suitable to a more specialized journal than Nature Comm., I would urge the authors to work on their presentation to make it more accessible to a broader audience. I think that both questions are relevant to a broader audience, also beyond the application to protein sequences, and this audience should be addressed rather than the very specialists of the very specific question.

We appreciate the reviewer's suggestions, and have updated the text to make it more broadly accessible. Our results will be of interest outside the specific context of protein sequence modelling, since they are relevant for machine learning, natural language processing, and generative sequence models more generally.

We have rewritten the technical sections of the introduction (page 2, 3, ms), to better put our results in a broader context. That is, we better describe the current state and challenges of generative model evaluation, and in particular have added a discussion of common model evaluation strategies used in Natural Language Processing (NLP), as these do not test for the forms of mis-specification which appear to be common in protein sequence datasets, involving extensive chains and networks of interactions causing long-range covariation across the sequences.

Besides this general remark, there are a number of major points to be addressed by the authors:

1) One of the major technical novelties of the paper is the measure r20 (Maybe I missed something but I did not understand the meaning of this name), which systematically compares higher-order statistics in "real" test data and sequences generated from the models. There are a few remarks and questions concerning this measure:

To clarify, the r20 measure was named after the fact that it is computed using a Pearson-r score over the “top 20” subsequence frequencies for each set-of-positions. We have also added a more detailed description and diagram of r20 (Figure 3a, ms), in response to one of the second reviewer’s questions, described in that response below.

(a) The authors insist that it takes into account higher-order statistics, saying that the existing literature hardly goes beyond one- and two-site terms. This is not entirely true, several papers consider higher order terms like three-point correlations or distributions of sequences when projected onto dimensionally reduced spaces like done in RBM, Hopfield models, VAE etc etc. showing non-trivial moments beyond the first two. In this sense, the authors overclaim the novelty of the approach, even if r20 is more systematic in its construction than any of the published examples I know.

As the reviewer notes, there is existing literature computing 3rd and even 4th order sequence statistics but in a nonsystematic way, which we had cited, and we have now included more references along this line, Refs. [Theys et al, 2018; Shekhar et al, 2013; Mann et al, 2014; Ferguson et al, 2013; Shimagaki & Weight, 2019; Quadeer et al, 2020] . To clarify our contribution, the novelty of the r20 measure is that it generalizes and systematizes the assessment of a GPSM’s ability to model covariation to high orders, while still recapitulating previous measurements for the lowest orders. We think that generalizing the measure in this way motivates comparison with current standard measures in machine learning literature for sequence-based and natural language models, for instance perplexity. This provides additional perspective showing how current systematic measures do not probe higher-order covariation as directly as r20, and our results with r20 highlight how higher-order covariation can be used to distinguish models in ways these other measures cannot.

We have added a discussion of this (pages 8, 9 and 13, ms). We have also extended the discussion of the r20 metric (pages 8, 9, ms).

(b) The construction of r20 consider high-frequency n-tuples for n<=10. I would expect the highest frequencies to be related to combinations of conserved amino acids, so at least part of the signal is explainable by the single-site statistics (explaining why also the profile model has

quite a good performance for smaller values of n). Instead of frequencies, higher-order statistics can be better detected by connected correlations or cumulants. I also would expect that low-frequency words may be informative (if expected at higher frequencies based on lower-order information), but possibly cannot ne detected reliably having limited statistics for larger values of n. So would it be possible to base r20 rather on connected correlations than on frequencies?

This is an insightful idea. Following the reviewer's suggestion, we have performed the "connected correlation" equivalent of the r20 measurements, which we will call "cc-r20", and added these results to the SI. The results for kinase are shown in the plot below for the synth-1M test case. We find the cc-r20 results (left) are qualitatively similar to our original r20 measurements (figure 3b of the main text, right), and lead to the same conclusions: The Potts model matches the target values well while the VAE models are lower.

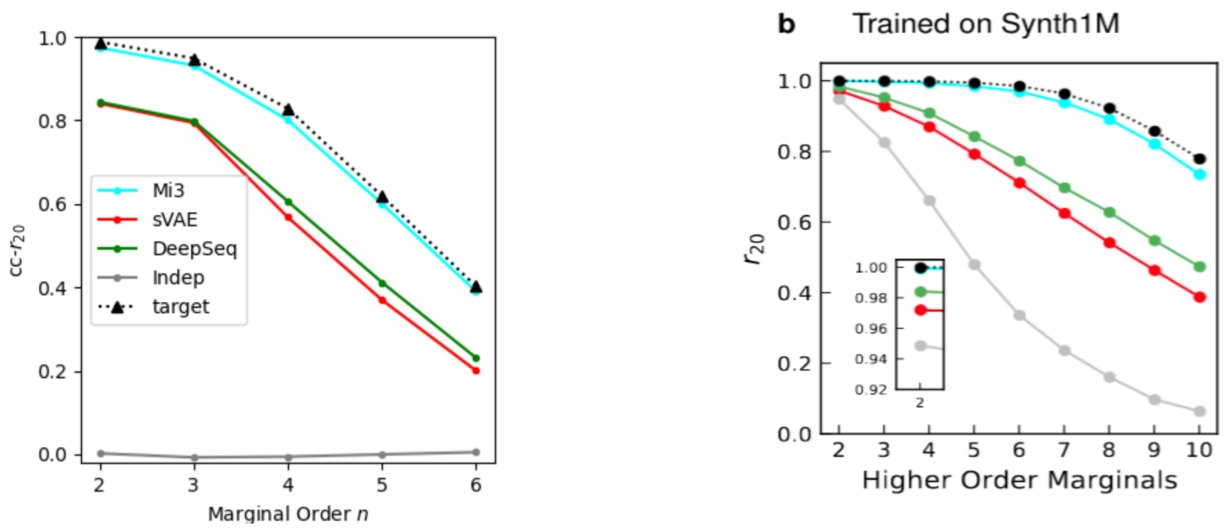

cc-r20 measurements for the kinase family, for the synth-1M test in which GPSMs fit to a training MSA of 1M sequences is compared to a target Potts model. Left: cc-r20 computations. Right: For comparison, the r20 results for the same dataset, reproduced from Figure 3b, ms.

As the reviewer noted, the cc-r20 results test statistics which cannot be predicted using a site-independent model. This emphasizes the inability of the independent model to capture the sequence covariation, since all of the connected correlations are close to 0 for the independent model.

We had commented on the connected correlations in the SI previously, but had not used them to construct a cc-r20 score because we expected cc-r20-like measures to be more sensitive to finite-sampling statistical effects (estimation error), limiting their use for higher orders of marginals. In our new tests, consistent with our expectation, we find that even using validation MSAs of 6M sequences we can only reliably evaluate cc-r20 up to 6th order, in contrast to up to 10th order for the original r20 scores. While we could potentially go beyond 6th order by

increasing the size of our test MSAs beyond 6M, as discussed in the SI the necessary MSA sizes quickly diverge.

Additionally, we agree that low-frequency words should be informative but cannot individually be directly reliably measured, but nevertheless the r20 score tests for them indirectly: If epistatic interactions increase the frequencies of low-frequency words, of which there are many, this necessarily reduces the frequency of the highest-frequency words since the frequencies must sum to 1. Thus, r20 indirectly measures the balance in probabilities between the low-frequency words and the individual highest 20 frequency words.

For these reasons, we have left the original r20 measurements in the main text, as they better highlight model accuracy to higher (10th) marginal orders than the cc-r20s. Nevertheless, the cc-r20 results up to 6th order are useful and interesting. Since the reviewer has suggested this and other variations of the r20 score, we have also added a discussion of alternatives to the r20 score, including the cc-r20 scores, with additional plots and discussion in SI.

This information is now discussed in the main text (page 9, ms), and in more detail (Section 7, SI).

(c) For each n, 3000 combinations of n sites are randomly selected. For larger n, this leads to a very very sparse sampling of all combinations of sites (e.g. for proteins of 200 sites and n=10, there are ~2e+16 choices, and only 3000 are considered). Most combinations of multiple sites show very small correlations (e.g. for n=2 those in the gap in Fig 2a-c), while the large correlations form a very sparse network (e.g. points beyond +-0.05 in these plots). So they are hardly ever taken into account in r20. Shortly, if relevant higher-order terms are sparse, they will not be seen by the random selection of sites in r20, and not be taken into account in comparing test data and synthetic data.

The reviewer accurately notes that the marginals are sparsely sampled by r20, especially at higher orders, but we argue that despite this sparse sampling the r20 metric is still able to determine whether higher-order covariation can be captured by a GPSM in many cases. This can be understood through the effect chains and networks of epistatic interactions on the MSA marginals.

The Potts Hamiltonian is the easiest that can be used to interpret or explain this. The Potts model is parameterized by pairwise interaction terms Jijab (couplings), and Potts models fit to protein MSAs have sparse couplings as we find many Jijab are close to 0. However, as we explained in our introduction and in more detail in SI, it is important to distinguish couplings Jijab (in the model parameters) from covariation (based on the MSA marginal statistics, such as connected-correlations). The Potts model usually generates higher than pairwise covariation despite only having pairwise couplings. In SI we showed an example where the Potts model generates triplet covariations. This arises due to chains of the pairwise couplings, for instance if

position 10 is coupled to position 20 which is coupled to position 30, we find nonzero triplet-connected correlations across the three positions. Through long chains and networks of couplings, we find there is widespread covariation to varying degrees across the positions in the MSAs we studied. This means that introducing a new pairwise coupling to the model at positions i,j will affect the marginals at other positions k and will affect many higher-order marginals (word frequencies) throughout the MSA.

In this way, as long as there are enough chains or networks of interactions, even if the interactions themselves are sparse, we expect that introducing a new model interaction at one set of positions will affect the marginals at many other sets of positions. This means that if a higher-order interaction term is necessary to model the MSA, the r20 score is likely to distinguish which GPSMs are well-specified for this MSA since many higher-order marginals throughout the MSA will be affected.

We have performed a numerical test to demonstrate this, by artificially introducing a single 3rd order interaction term to a protein sequence Potts model and showing its effect on r20. In this test, we choose a single arbitrary set of three positions ijk in a natural sequence MSA, only conditioned to avoid choosing highly conserved positions. With this condition, the most common character at each of the three positions we chose in all our tests has roughly 10% to 20% frequency. Taking a Potts model parameterized based on this natural sequence MSA, we then artificially introduce a single triplet interaction parameter, J^ijk_abc for the characters abc corresponding to the most likely character at each of the three positions, creating a “Potts+3” model, from which we generate sequences. In other words, we add the value J^ijk_abc to the Potts energy function only for sequences with the triplet abc. We choose a value of J^ijk_abc = -log(2), since this will approximately double the frequency of the word “abc” assuming it is fairly rare. We then fit new models to this Potts+3 biased MSA, and compute r20 for these new models relative to the target Potts+3 model. Further details on the procedure are included in Section 9, SI.

We did this for two of the protein families we tested (more details on these below), RRM and response regulator. In both cases, the introduction of this single triplet term causes a detectable decrease in the r20 score, particularly at order 10. In comparison, if we perform the same test but without adding the triplet term, the Potts result matches the reference target, and we find no decrease in r20 at order 10 beyond the effects of finite sampling or estimation error (see plots in answer to the reviewer’s question about other protein families further below). This demonstrates a case in which the r20 score can detect model misspecification even for sparse higher-order interaction terms.

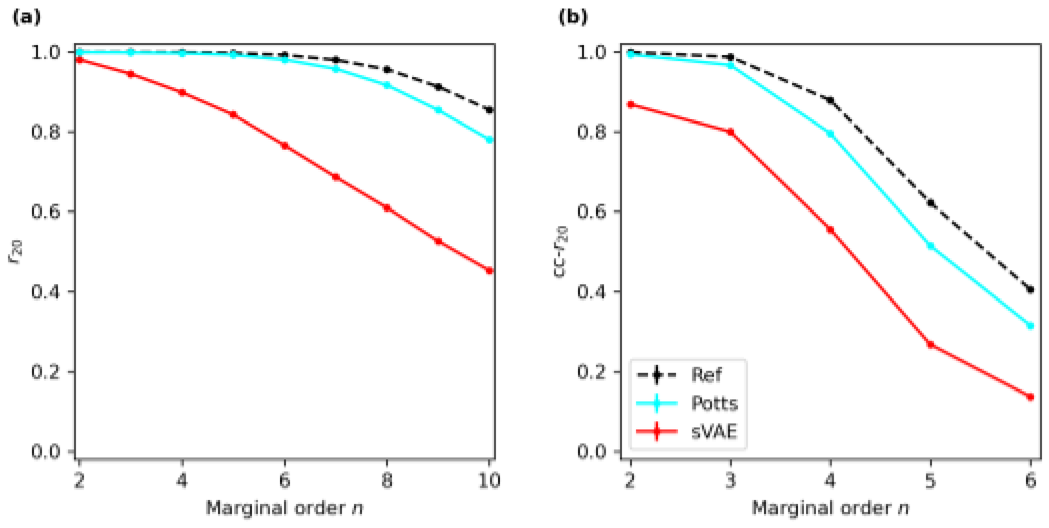

R20 results for the Potts+3 target model for the Response-Reg protein family. (a) The r20 scores (b) the cc-r20 scores. Note the deviation of the Potts model from Ref for n=10. The error bars (standard error in the mean) are too small to see.

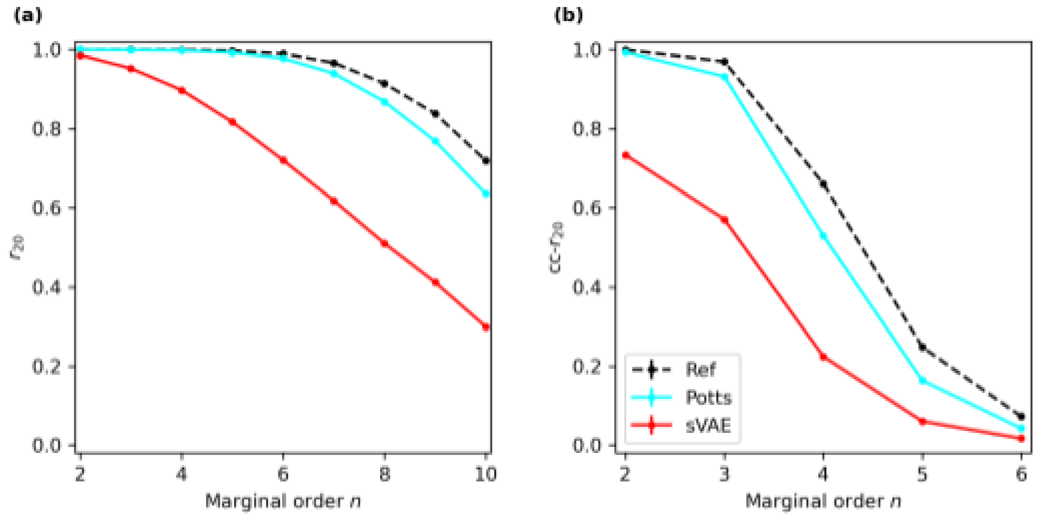

R20 results for the Potts+3 target model for the RRM protein family. (a) The r20 scores (b) the cc-r20 scores. Note the deviation of the Potts model from Ref for n=10. The error bars (standard error in the mean) are too small to see.

As an example of how r20 can be affected, consider the case where we introduce a single triplet term at positions ijk to an existing Potts model, as in our Potts+3 numerical test just described. If position i is strongly pairwise coupled in the Potts model to position x, and j to y, and k to z, then introducing a triplet term at ijk will also affect the marginals at positions xyz, generating higher-order covariations at xyz not well captured by a Potts model. Thus, even if the r20 calculation does not directly sample positions ijk where the interaction applies, it will likely

sample many other sets of positions xyz whose marginal frequencies are affected by the triplet interaction at ijk. Our numerical test of r20 for the Potts+3 model supports this interpretation.

This triplet interaction test is artificial in the sense that we added a triplet interaction term without any biological justification. Because the existence of higher-order interaction terms in natural sequence probability distributions is still debated, we do not know the strength and sparsity with which such interactions might appear. Nevertheless, our test provides a demonstration of a case in which r20 can be used to detect model misspecification even when higher-order interaction terms are sparse, and even for a single triplet interaction term as shown in this test. Additionally, for realistic MSA sizes available from Pfam, of up to 10K sequences as described further below, it is unlikely that properties of the natural sequence probability distribution can be fit better by including triplet terms, because finite size effects obscure the contributions of higher interaction terms, limiting our ability to reliably measure r20 to the higher orders at which the effects of the triplet term become apparent.

While we believe r20 will often detect misspecification of a model failing to include necessary higher-order interactions, we agree with the reviewer that the r20 score cannot absolutely rule out the possibility that sparse higher-order effects are necessary to model an MSA in all cases. For instance, if we introduce a single triplet interaction to a site-independent model which lacks the chains and networks of interaction of the Potts model, in such a way as to avoid generating any pairwise correlations, r20 is likely to fail to detect the affected triplet, since most site-frequencies across the MSA will be unaffected by the interaction. As we noted in results, none of the GPSM metrics we tested on its own can give a complete picture of the generative capacity, since to exhaustively test generative capacity one must deeply sample the distribution P(S) for all S, which is not feasible. But, in the case of protein-family MSA datasets where covariation and chains of interactions are important, and enough sequences are available, we expect r20 to be a powerful discriminative metric.

We have updated ms and SI to include this discussion of sparse triplet interactions. We discuss these issues in the main text (pages 8, 9, ms), and in SI (Sections 6, 9, SI).

2) The ms provides analyses on synthetic data coming from a pairwise Potts model. The authors admit that this choice favors the Potts model also in model learning, since the exact model can be learned (and will be learned by maximum likelihood in the limit of infinite data). However, it would be important to see how the measures perform, when systematic higher-order terms are introduced into the model. Does r20 work, e.g., perfectly up to some k-1 for Mi3, when there are fields, pairwise interactions and true k-body interactions exist in the data-generating "true" model (I mean interactions not decomposable into lower-order interactions, like k-spin interactions J s1 s2 ... sk in the Ising model)? This would be important to make deviations in r20 from the optimal perfomance better interpretable and thus usable for the systematic construction of better models.

The reviewer raises two interesting points we wish to address.

First, the reviewer notes that some of our synthetic tests potentially favor the Potts model since the target distribution is a Potts model, as we discussed in the manuscript (page 5, ms). But we would like to emphasize that we also tested GPSM model performance when the synthetic data was generated by sVAE rather than a Potts model, specifically to account for the potential bias the reviewer describes, and found that the Potts model predicted the target distribution most accurately according to our metrics, while the VAE model predictions were less accurate (Figure S6, SI). This demonstrates that our results are not due to a bias in the test towards Potts models.

Second, the reviewer points out that it would be useful to investigate our r20 metric's behavior when the target distribution requires higher than pairwise interaction terms to correctly model. The synthetic test we performed using the sVAE as the target model potentially does this, if one accepts that the VAE captures higher-order interaction effects as has been suggested in literature, however it is difficult to show this is really the case as our results for the VAEs using r20 demonstrate.

But more directly following the reviewer's suggestion, in our Potts+3 test described above in response to the previous question, we introduced a single triplet interaction term, which is a "true k-body interaction", and observed an effect on r20. This test demonstrates that r20 is sensitive to model misspecification caused by failing to model higher-order interactions, even if such interactions are sparse.

3) Concerning still the synthetic data, only dataset of 10K and 1M sequences were used. Many protein families are smaller, so it would be interesting to see results for smaller dataset. Does, e.g., the simpler independent model win for small datasets? Furthermore, it is said that for 1M the out-of-sample error is negligible for VAE, but these are later said to have 2.7M parameters. I would naively expect the out-of-sample error to vanish when the sample size is far bigger than the number of parameters.

Overfitting is an important concern, and we have previously presented detailed results about it for Potts and Independent models (Haldane & Levy, 2019). There, we discussed how, contrary to common intuition, the degree of overfitting (i.e. out-of-sample error) of these models cannot be gauged by simply comparing the number of parameters to the number of sequences, e.g. 1M sequences vs 2.7M parameters. For instance, overfitting also depends on the level of sequence conservation. Previously, we showed that out-of-sample error is virtually eliminated in some conditions even when the number of training sequences is orders of magnitude smaller than the number of parameters of the model, and we illustrated this with some simple examples (Haldane & Levy, 2019). This is an important point which, motivated and reminded by the reviewer's comment, we have added to the manuscript (page 5, ms).

Previously, we showed how, as the reviewer hypothesized, the independent model outperforms the Potts model for very small dataset sizes (Haldane & Levy, 2019). We derived mathematical results explaining why and how this happens, showing it is a function of the sequence length (L), number of sequences in the MSA (N), and the degree of conservation of the MSA (a quantity we called chi).

In the current manuscript, we also numerically tested for the magnitude of out-of-sample error on the 1M dataset, to show it is very small, as shown in Figure S2, SI. We show that when doubling the training size from 1M to 2M sequences the improvement in the model according to our GPSM metrics was negligible. We believe this is strong evidence that out-of-sample error is negligible for the 1M test. Our prior analysis supports this conclusion by providing an explanation for why one should expect 1M sequences to be sufficient to virtually eliminate out-of-sample error, even though the number of parameters is larger (Haldane & Levy, 2019).

Because we have already focused on the effects of MSA depth and on the very-small-dataset case in detail (Haldane & Levy, 2019), and because many studies of learning methods for protein MSAs focus on the well-sampled protein families with moderate-sized MSAs, we would like to focus on the 1M and 10K dataset sizes in this manuscript.

4) Concerning the analysis for real proteins, only a single family is analyzed, but far reaching conclusions are drawn for proteins in general. Pfam contains almost 20 000 protein families, with downloadable MSA reaching from very few (<10 sequences) to almost 1M sequences. I think it would be easy to assess the robustness of the claims for substantially larger datasets of variable MSA depth.

We have followed the reviewer's suggestion, and have now included three more protein families: ABC transporter, RRM, and response regulator. These three families all have a large number of available sequences, allowing us to perform the natural analysis with the same 10K training/validation MSA splits as for Kinase, and we chose them to have diverse properties, with different sequence length, sequence conservation, structure, and biological function. Our original kinase MSA has sequences of 232 residues after processing, that of ABC-transporter has 125 residues, response regulator has 107 residues, and RRM has 48 residues, so we are testing a wide range of sequence lengths. The sequence length affects the balance of pairwise vs single-site terms in the Potts models, since the number of single-site terms grows as L while the pair-sites grow as L^2, so the amount and strength of higher-order covariation could differ among these families. Similar reasoning applies to even higher-order interactions.

The results for all families are qualitatively similar to our previous results, and do not change our conclusions. These results are presented in SI (Section 11, SI), and discussed in ms (page 5, ms).

Below we highlight the r20 results for these protein families, for the natural 10K test and synthetic 1M test, performed the same way as we did for kinase in the main text. That is, in the Natural 10K test we fit the GPSMs to the 10K natural training MSA, and compare GPSM generated sequences to a 10K natural target MSA. In the Syntetic 1M test, we use the Potts model fit to 10K natural sequences as the target probability distribution P^0(S), and then fit new models to 1M sequences generated from this target probability distribution. We then compute r20 and cc-r20 values comparing 6M sequences generated from each model to 6M sequences generated from the target model. The relative performance of the different models is very similar to our kinase result (Figure 3, ms). See Figure 3a, ms, and discussion for more details on the procedure. Note that, as described in the main text page 8, in the natural analysis the expected estimation error (black line) is only approximate.

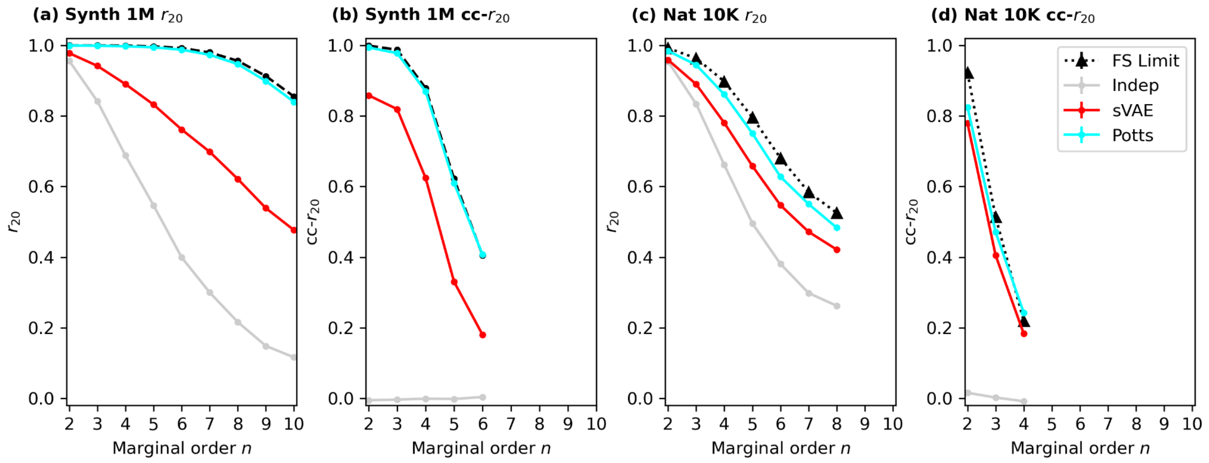

R20 results for the Response-Regulator protein family. (a) The r20 scores in a synthetic test with 1M training sequences with a Potts model synthetic target distribution. (b) the cc-r20 scores for the same 1M test. (c) The r20 scores for the natural data analysis (d) The cc-r20 scores for the natural analysis.

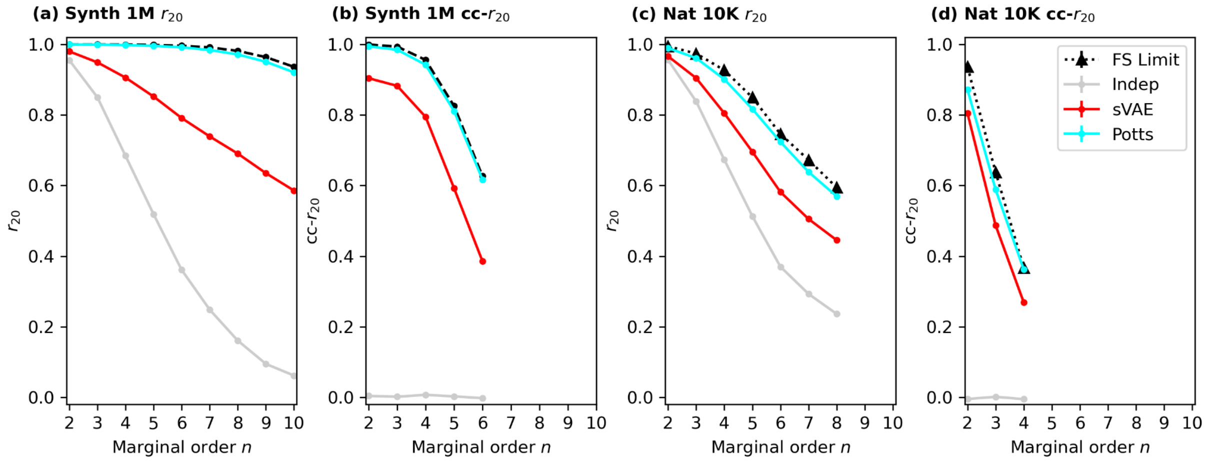

R20 results for the ABC-transporter protein family. (a) The r20 scores in a synthetic test with 1M training sequences with a Potts model synthetic target distribution. (b) the cc-r20 scores for the same 1M test. (c) The r20 scores for the natural data analysis (d) The cc-r20 scores for the natural analysis.

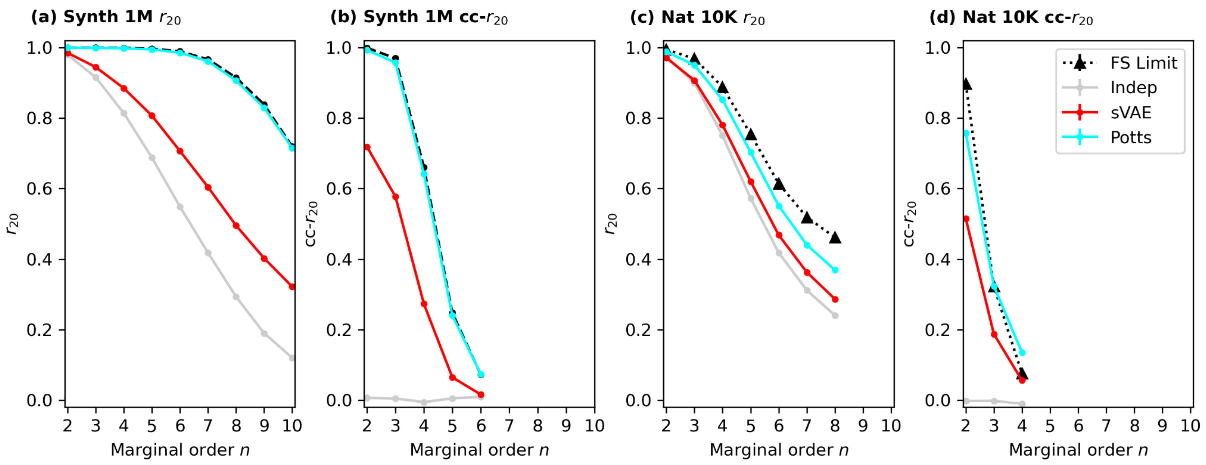

R20 results for the RRM protein family. (a) The r20 scores in a synthetic test with 1M training sequences with a Potts model synthetic target distribution. (b) the cc-r20 scores for the same 1M test. (c) The r20 scores for the natural data analysis (d) The cc-r20 scores for the natural analysis.

Note that plots for the Potts+3 model, in our other answers to the reviewer, can be compared to these plots to illustrate the triplet interaction effect described there.

5) I did not find anything about code availability. It would be great to have a code implementing the different measures, given to MSA as input (one the target MSA, one the synthetic one).

All of our code is publicly available. We have now clearly noted this in the manuscript, and we also link it here for the reviewer's convenience:

The code for our overall workflow and plotting is at:
https://github.com/alagauche/generative_capacity_metrics
The sVAE code is at:
https://github.com/ahaldane/MSA_VAE
The code used to build the Potts models is at:
https://github.com/ahaldane/Mi3-GPU
The code used to compute r20 and cc-r20 is at:
https://github.com/ahaldane/HOM_r20

Minor remarks (in order of appearance):

i) A protein of length 100 would have about 1B parameters and not 10B.

We thank the reviewer for finding this error, and we have corrected it (page 2, ms).

ii) The last two paragraphs on page 2 are an example of the extremely technical, hard-to-read style of the paper.

We have updated these and related paragraphs, and appreciate the reviewer pointing out these hard-to-read paragraphs (pages 2, 3, ms).

iii) The likelihood in Sec' "Target distributions" should either be given as the product of probabilities of sum of log-probabilities.

We again thank the reviewer for finding this error and have made the correction (page 3, ms).

iv) Fig. 1 is a bit complicated, but I have to admit that I have no good suggestion to simplify it. Maybe the authors have one?

We have extensively updated this figure in a way we hope is easier to understand (Figure 1, ms). The previous version of the figure had an emphasis on the practical procedure we carried out, while in this new version of the figure we tried to emphasize the different forms of tests we performed. The new figure is below.

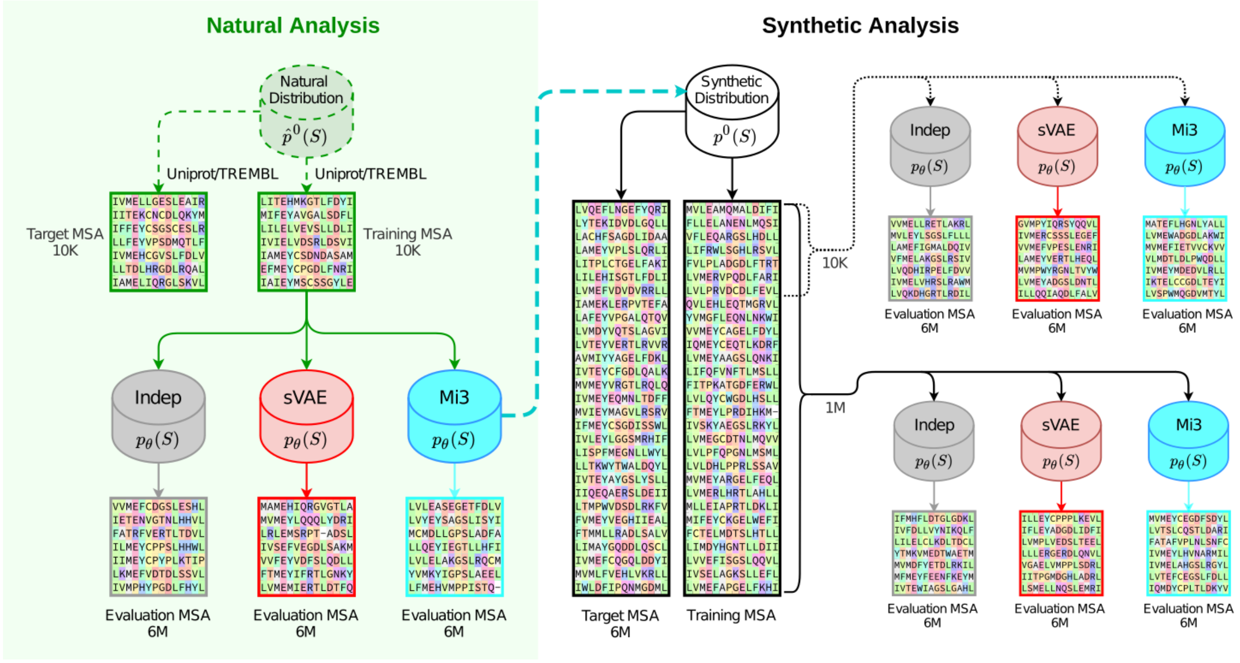

Workflow of Natural and Synthetic Analyses} We present two analyses, one Natural (left) and the other Synthetic (right). In both analyses, we train three types of GPSMs on protein MSAs, which are Indep (gray), VAEs (red), and Mi3 (cyan). To compare performance across GPSMs, we generate an Evaluation MSA of $6M$ synthetic sequences from each GPSM after training, and test generative capacity by comparing the Evaluation MSA to a

Target MSA that is non-overlapping with the Training MSA but was sampled from the same target probability distribution. \textbf{Left} In the Natural Analysis, we perform a single experiment where we train the GPSMs on $10K$ protein sequences taken from Uniprot/TREMBL. In this case, the natural target probability distribution is unknown, so we demarcate its container with a dashed line and define it as $\hat{p}^{0}(S)$ to emphasize this unique property, in contrast to all other probability distributions which we know exactly because they are actually GPSMs. The dashed lines leaving the Natural Distribution indicate that the non-overlapping $10K$ training and target MSAs are the result of aligning and phylogenetic filtering of sequences obtained from Uniprot/TREMBL in a process described in Methods and SI. In the Natural Analysis, we are data-limited to $\sim10K$ samples for the non-overlapping training and target MSAs (see SI). \textbf{Right} In the Synthetic Analysis, we perform two experiments on sequences sampled from a known Synthetic Distribution, $p^{0}(S)$, from which we can generate arbitrarily large MSAs, maximizing precision in our generative capacity measurements in a way that is unavailable in the Natural Analysis due to inherent data limitations. We define the Synthetic Distribution as the Mi3 GPSM distribution constructed in the Natural Analysis. In one experiment (top right), we train the GPSMs on only $10K$ synthetic sequences from $p^{0}(S)$ and use a $6M$ evaluation MSA, the same amounts as in the Natural Analysis. However, we perform generative capacity measurements on a target MSAs of $6M$ sequences, removing estimation error from the measurements. In the second experiment (bottom right), we train the GPSMs on $1M$ training sequences, which include the $10K$ from the first experiment. With arbitrarily large training and target MSAs, both out-of-sample and estimation error are removed from the generative capacity measurements, leaving only specification error.

v) Sec. "Pairwise covariance correlations": individual Cijab may be zero, but this is no indication of a lack of co-variation. Typically a scalar correlation measure taking all combinations of amino acids has to be taken (l1 or l2 norm, mutual information).

We agree and thank the reviewer for finding the inaccurate statement “The scores equal 0 if the two positions do not covary” and have updated it to the more correct statement (page 6, ms) “If $C^{ij}_{\alpha\beta}$ equal 0 for all $\alpha\beta$ then the positions i,j do not covary.”

We agree that, for a particular position-pair ij, some of the Cijab may be zero even if the positions covary overall. Since we include all residue pairs ab in our analyses, then if there is covariation we will detect it.

vi) Covariances close to zero in Fig. 2 are omitted. Are they included in the determination of the reported correlations rho?

Yes, the covariances close to zero are included in the reported correlations. We have noted this in the text (Figure 2, caption, ms).

vii) In Fig. 2d-f, the r20 Nat-Target decays much faster in the natural data than in the synthetic data. Do the authors have any explanation why?

The Nat-Target r20 decays much faster than for Synth-Target because many fewer natural sequences are available to compute r20 in the natural tests than in the synthetic tests, so the “estimation error” is much larger in the natural test, which decreases the maximum attainable r20. We only have 10K natural sequences available to compute r20 in the natural test, while in the synthetic test we use 6M sequences to compute r20.

Most protein families in Pfam have up to 10K effective natural sequences in total available to test, as we demonstrate in Section 12, SI, "Typical natural sequence dataset MSA size". One of our results is that, at MSA depth of ~10K, estimation error masks GPSM performance according to r20. We provided a detailed mathematical explanation for this phenomenon in Section 8, SI, "Analysis of estimation error". In summary, the r20 score depends on estimating

n-tuple (word) frequencies, but for larger n the individual word frequencies become very small, requiring large MSAs to estimate reliably.

In contrast, for the synthetic tests we can generate arbitrarily large MSAs, and in practice we use target and validation MSAs of 6M sequences. This is one of the motivations for performing the synthetic tests, as only in synthetic tests can we greatly decrease estimation error caused by small target MSAs, by generating enormous target MSAs from the target model.

We have updated the language with the paragraph beginning “In the natural analysis” (page 8, ms), to more explicitly explain this.

viii) Concerning Hamming distances, not only Hamming distances between data of the same type are interesting, but also inter-dataset distances. For illustration, imagine that data are sampled from a distribution P(S). In the first case, the target is sampled, and the generated is just a copy of it. So the distance distributions of the two are the same, but the inter-distances have a peak in zero. If the two sets are independently sampled, the inter-distribution will also be equivalent to the intra-distributions. And last but not least, if the two are sampled independently, and in the generated dataset all positions are globally permuted, the intra-distributions will be equivalent, but the inter-distributions larger.

We have performed the test the reviewer suggests. This test is a demonstration that our inference is not affected by a kind of overfitting effect in which the sequences generated by the model are identical or nearly identical to the target sequences, for instance if sequences generated by the model are repeated.

We performed this test using the kinase synth-1M dataset, in which we have a target MSA and a training MSA both generated from the target probability distribution. We fit a new Potts model to the training MSA, and from this Potts model we generate an evaluation MSA.

We then computed both the intra-hamming distance distributions separately for the target and evaluation MSAs, as well as the inter-hamming distance distribution between the target and evaluation MSA.

We find all three of these distributions are virtually identical, confirming that the GPSM Potts model fits the target well, and that the validation sequences generated by the GPSM are as distinct from the target MSA as they are from each other, thus ruling out an overfitting effect of this kind. As the reviewer notes, the intra-hamming distance distribution is mathematically identical if we shuffle the MSA columns, but we find the inter-distribution distance is much larger after shuffling one MSA’s columns, as the reviewer suggested.

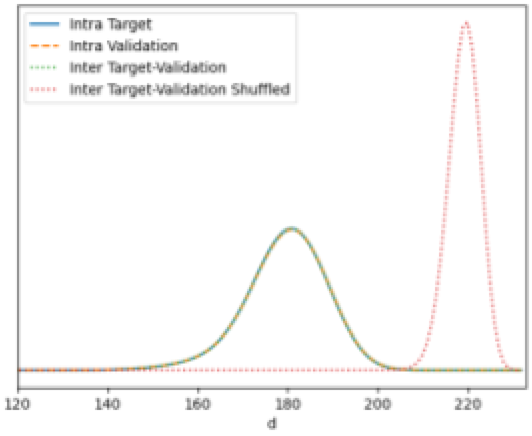

Hamming distance distributions for the Kinase protein family,where the target distribution is a Potts model (fit to natural data), and the GPSM is another Potts model fit to a 1M training MSA generated from the target distribution. Hamming distance distribution are calculated using 30K sequences for all MSAs.

ix) Concerning the statistical energy correlations, the data-generating model has to be known. In this case, it would be interesting to consider models with higher-order couplings, which cannot be reproduced fully by the Potts model, as mentioned before.

As the reviewer suggests, we can consider the statistical energy correlations in the context of higher-order couplings, using the Potts+3 model we described above which includes a triplet interaction parameter. Here we essentially repeat our procedure for statistical energies from the main text, but using the Potts+3 model.

To summarize this procedure, we generate a training MSA of 1M sequences from our Potts+3 model with a triplet interaction, then we fit a new Potts model (without triplets) to this training MSA. We can then score sequences of a target MSA generated from the Potts+3 model, both using the Potts+3 model (including triplet term in the energy) and using the inferred Potts model. The results using a target MSA of 100K sequences is shown below for the RRM and Response-reg protein families.

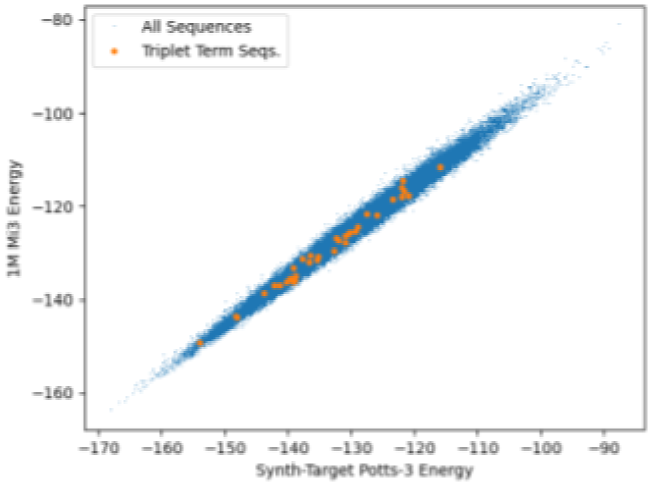

Statistical Energy correlations for the RRM protein family, when the target model is the Potts+3 model with a triplet interaction. Each point represents a sequence from the validation MSA. A random sample of about 50 sequences from the validation MSA which have the triplet interaction are shown in orange.

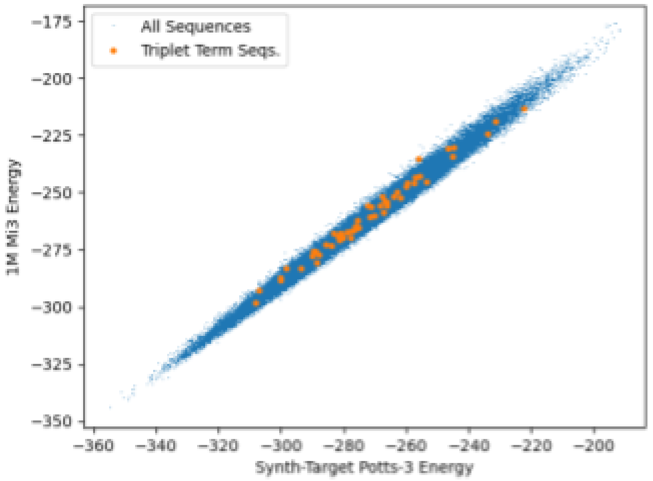

Statistical Energy correlations for the Response-reg protein family, when the target model is the Potts-3 model with a triplet interaction. Each point represents a sequence from the validation MSA. A random sample of about 50 sequences from the validation MSA which have the triplet interaction are shown in orange.

The effect of the triplet interaction term is not easily detectable from the statistical energy comparison: One could expect that the Potts model without triplet interactions would less accurately model the Potts+3 sequence energies, for instance for the sequences which have the favorable triplet combination (shown in orange in the plots). However, the magnitude of the triplet interaction in this case, which we fixed at log(2) = 0.7 energy units, is smaller than the typical error of the reconstructed energy (the width of the blue distribution in the plot), which has root-mean-square error of 2.4 for Response-reg and 1.1 energy units for RRM.

Our conclusion from this is that r20 is better suited to distinguish whether a model correctly accounts for the higher-order-terms needed to model a dataset. In the statistical energy plots, the effect of the triplet interaction term on individual sequences is negligible relative to the contributions from all the other interactions throughout the sequence. The r20, on the other hand, averages MSA statistics across sequences, and focuses on marginals, so is not limited in this way.

We have included these plots and their discussion (Section 10, SI), with a note in ms (page 2, ms).

x) Note that E = -log P cannot be calculated analytically for Mi3, only up to a constant (the free energy).

We have added a description that the Potts E is ambiguous up to a constant in the methods (page 14, ms).

xi) The Methods section lack details about the VAE structure. How are hyperparameters selected (like the number and size of layers), and the dimension of latent space? How do the authors exclude, that their results about the limited generative capacity of VAE is not just a result of not optimal choices? I am astonished by only 7 dimensions in latent space.

The details on the hyperparameter choice and architecture of the sVAE is given in Section 1, SI, but we have moved some of it to the methods section of the main text for clarity (page 15, ms).

To summarize, our VAE, which we call sVAE, is nearly identical in architecture to EVOVAE, a published state-of-the-art VAE-GPSM (Sinai et al, 2018). sVAE also performs very closely to another state-of-the-art GPSM, DeepSequence, in all metrics, including our most sensitive metric, r20. Interestingly, both VAEs perform well on all "standard" GPSM metrics, and are almost indistinguishable from Mi3, except for on r20, which is the only metric that directly measures higher order covariation. Together, these findings represent strong evidence that the limited generative capacity of VAEs in general is not due to suboptimal architectural or hyperparametric choices, but due to the properties of the variational autoencoding approaches used in the state-of-the-art VAEs we tested. In the Discussion in the manuscript, we list some shortcomings and their possible solutions for variational autoencoders broadly.

In response to reviewer's question about hyperparameter tuning, we have updated Methods in ms to clearly indicate that for sVAE, as with EVOVAE, hyperparameters were selected using a grid search on the number of dense hidden layers (1-4), number of units per layer (50-350), and inclusion/exclusion of dropout and batch normalization layers between each hidden layer. We already emphasized this point in Section 1, SI, "sVAE Implementation", and delineated our

departures from EVOVAE in detail. For example, we explained why we removed the scaling factor of *Lq* on EVOVAE's reconstruction loss.

For the reviewer's question about the choice of latent dimension as 7, we have updated Methods in ms with a brief justification and clear reference to our Section 2, SI, "VAE model validation and hyperparameter selection" for details on this selection. In the SI, we justified our selection of 7 for the latent dimension in two key ways, and have added a third. Firstly, we tested varied number of latent dimensions from 1 to 10, as shown in Figure S3, SI, and found that an effect known as “posterior collapse” begins at 8 latent dimensions, which may indicate that 7 latent dimensions appear to minimally represent the data in the latent space, where greater than 7 latent dimensions results in collapsed or "extraneous" dimensions. Posterior collapse of this nature has been attributed to extraneous dimensions in the literature, which we cited (Dai et al, 2019; Lucas et al, 2019).

Secondly, we performed a completely independent "intrinsic dimension" analysis of the natural training data [Granata & Carnevale, 2016], which indicates that the intrinsic dimension of the data is appropriately 7. Lastly, we originally included a citation which estimates that the intrinsic dimension of protein families appears to range from 6 to 12 [Facco et al, 2019].

The reviewer is surprised that only 7 latent dimensions lead to the best fit. But interestingly even latent dimensions of l=2 and even l=1 still perform comparably to our models with l=7, as can be seen in the r20 plots in Figure S4, SI. In fact, a large fraction of studies in literature of VAEs applied to protein sequences use only l=2 latent dimensions (Riesselman et al, 2018; Ding et al, 2019), perhaps because this makes the latent space easy to plot on the cartesian plane. The neural network of the VAE’s decoder allows it to map the l=1 real number line or l=2 cartesian plane to a very diverse range of output sequences.

To conclude, I think that the ms addresses an interesting question and shows interesting evidences, but should be complemented by further analysis to fully support the author's claims.

We thank the reviewer for their useful and appropriate suggestions and comments,

To summarize again, we performed a number of new calculations in response to the reviewer’s comments, each of which took significant computational and development time, and we also significantly expanded our discussion of which types of model misspecification the r20 score can detect, in response to multiple of the reviewer’s questions.

<u>Reviewer 2 (Expertise: DL for protein structural prediction):</u>

Review of "Generative Capacity of Probabilistic Protein Sequence Models" by McGee, Novinger, Levy, Carnevale, and Haldane.

The authors present a systematic comparison of classic ("shallow") and modern ("deep") models for learning epistatic interactions in via parametric probability distributions of protein sequences given a fixed alignment frame. (High-order) epistatic interactions means the co-evolution of two (or more) sites in a protein sequence, meaning, that they vary in the same way between different homologous protein sequences. This exercise is useful since understanding these interactions can be important to do protein engineering, protein structure prediction, prediction of the impact of genetic variations on protein functon(and potentially disease), or to do protein design. There is and has been extensive activities in this area of research, and interest has surged in around 2013 when several groups independently found that Potts models were very good at capturing pair-wise epistatic interactions. With the mainstreaming of deep learning methods deep generative models have seen several applications as they promise to capture more complicated multi-site epistatic interactions due to the superior representative power. The authors aim to test whether this promise holds.

The somewhat surprising result the authors present is that the simple Potts model outperforms the more sophisticated deep models when it comes to faithfully reproducing high-order epistatic interactions.

The authors use natural and synthetic data-set, which represent a protein-family aligned by multiple sequence alignment (MSA). The authors compare two "shallow" models: independent sites (no interaction between sites in MSAs) and a Potts model (adding pair-wise interactions to the independent sites model), and two "deep" models corresponding to two different variational auto-encoder models. The authors use four different assessment criteria to see whether the epistatic interactions in the data are faithfully captured by the learned models. Using samples generated from the learned model they compare: Pair-wise covariance, higher order marginal statistics, Hamming distance distributions, and statistical energies, between model samples and "true" reference data. The latter test is only done for synthetic data since statistical energies are unknown in true data-sets.

Overall, I like the study. However, it is highly technical and many of the potential explanations used to rationalize the findings are

well-known, and are linked connected to inappropriate use of deep learning methods outside the domain of applicability -- using continuous variable models to learn multinomial data -- which brings both statistical sub-optimality and topological problems. Other problems are related to optimization and learning in these models. These sub-optimal assumptions are not made on the shallow models, although they could have been made to make the comparison fairer -- but also pointless. Nevertheless, I was disappointed that no effort was made to try to side-step these severe limitations. For instance, using a based transformer models could have resulted in a more level-headed comparison and within right application domain: sequence modeling. There are MSA-based transformer models described in the literature

https://www.biorxiv.org/content/10.1101/2021.02.12.430858v1.full

Including such a model in the evaluation would eliminate some of the known limitations of variational auto-encoder based models of sequence data from the equation and more fairly compare deep versus shallow models.

We thank the reviewer for these comments and suggestions, and would like to respond to a number of points:

1. We have updated the introduction and motivation in order to make the manuscript style less technical and better emphasize the scope of our conclusions.

As we noted in our response to reviewer #1, our results will be of interest outside the specific context of protein sequence modelling, since they are relevant for machine learning, natural language processing, and generative sequence models more generally. We have rewritten the technical sections of the introduction (pages 2, 3, ms), to better put our results in a broader context. That is, we better describe the current state and challenges of generative model evaluation, and in particular have added a discussion of common strategies used in Natural Language Processing (NLP) for model evaluation, as these do not test for the forms of mis-specification which appear to be common in protein sequence datasets, involving extensive chains and networks of interactions causing long-range covariation across the sequences.

2. Issues with using continuous variable models to learn multinomial (categorical) data, which is one potential source of VAE model error.

We would like to point out that most of the recent successes of deep learning lie in applications on data with discrete variables. Despite many open theoretical questions related to understanding of why continuous variable models work so well when applied on discrete data, there is no doubt that those models work well in practice, e.g. in natural language processing or categorizing images. We hope that discussion and experimental results in this paper provide

some useful insights about the differences between deep learning models and the more traditional Potts model.

The reasons for the behavior and potential limitations of the VAE are actively debated in literature. In the discussion in the manuscript, we discussed two currently proposed ways in which VAEs may fail to model a dataset. The first is the issue of “posterior collapse” and of the possibility of local minima in the likelihood function being optimized by the VAE (which may not be unique to VAEs, as discussed in Dai et al, 2019; Lucas et al, 2019). There are a number of different explanations for this phenomenon (Dai et al, 2019; Lucas et al, 2019). The second is the issue of “manifold mismatch”, related to the difference in topology of the target sequence space and the latent space, which is also an active area of investigation. Following on the reviewer’s suggestion, one can suppose that there is a topological mismatch between the categorical data (sequences made up of discrete characters) and the model’s latent space or embedding (a continuous, euclidean n-dimensional space, in the case of sVAE).

Additionally, while the VAE latent space is continuous, the sVAE model output is multinomial, and we are able to sample particular discrete sequences from the model. The VAE probability distribution P(S) gives a probability for each individual sequence, just as does P(S) for the Potts model. Internally, both models are parameterized by continuous variables, which are the couplings Jijab for the Potts model, and the neural node weights and latent dimensions for the VAE. In this sense, the models can be fairly compared as GPSMs. Likewise,even the Indep model, one of the simplest GPSMs, is a continuous multinomial model in the sense that its parameters are continuous probability values, yet we use it to model discrete sequences.

3. Testing a transformer model

The reviewer mentions a work of particular interest with publicly accessible code and a pretrained model: the MSA Transformer (Rao et al, 2021). This is indeed an impressive work and shows one example of how a large neural network with discrete variables can model a multinomial distribution through more and more complex transformations of hidden representations.

However, a comparison in this work comes with several caveats.

1) In the MSA Transformer paper (Rao et al, 2021), the possibility of generating de novo protein sequences is not discussed, though the model has generative capacity because it belongs to the category of autoregressive models, which can generate sequences through iterative inference as shown in prior work (Riesselman et al, 2019; Trinquier et al, 2021). We therefore base a comparison on the pretrained model of MSA Transformer (Rao et al, 2021) but using an adapted generative process akin to autoregressive models (Riesselman et al, 2019; Trinquier et al, 2021). In this method, starting from a given seed MSA, we mask out an entire protein sequence, and use the MSA transformer model to predict a probability distribution P(x_i|MSA) over the residues x_i at a masked position i, given the known residues in the remainder of the seed MSA. We replace one mask token by sampling a new residue from

P(x_i|MSA). This process is repeated for each residue in the missing sequence until all positions are filled in. We follow an easy first approach, where we first sample from the most conserved position, ordered by their site entropy s_i. In our test, we use groups of 256 sequences randomly sampled from the 10K natural RRM training MSA used in the main text, as seed MSAs.

2) MSA Transformer learns from 26 million MSAs containing sequences from different protein families, where each MSA is limited to 256 sequences, whereas the Potts model and VAE learns from 10,000 protein sequences from one particular protein family. In other words, the target distribution p^0(S) for the GPSMs we study in the main text corresponds to the probability of sequences in a particular protein family, while for MSA Transformer the equivalent probability distribution contains information about other protein families as well. Additionally, the sequences generated using the adapted sequence generative process for MSA Transformer depends on the seed sequences, as it generates sequences from the same protein family as the seed, which means that the generated sequences are not strictly i.i.d. samples from the model distribution p_\theta(S) over all protein families. Because of these differences we expect the inferred residue frequencies to be slightly different compared to a model that was trained for only one specific family. Additionally the MSA preprocessing is performed differently for the MSA Transformer, which again will affect the residue frequencies.

3) This generation approach is very slow, because it needs L calls to the model, where the model complexity is $O(L^2M) + O(LM^2)$, for a total computational cost of $O(L^3M) + O(L^2M^2)$. To speed up the generation process we mask out 32 of the 256 sequences to generate more than one protein at a time. Generation of 200,000 sequences of the RRM protein took approximately 2 days on a V100 GPU on an NVIDIA DGX-1 machine learning server.

4) MSA Transformer was trained with input noise to avoid over-fitting, which might have some undesired effects on the predicted output probabilities. This plays a similar role to the regularization and pseudocounts used to infer the GPSM models.

Although here we test it using our GPSM metrics, as seen from these caveats the MSA Transformer model is significantly different enough in goals and target dataset from the GPSMs we have studied in the main text, such that this model and our other GPSMs are not strictly comparable. Nevertheless, there are many similarities as well, and since it is possible to generate sequences from this model we can evaluate it using our metrics.

The results (see figure below) show that the generative capacity of MSA Transformer (msaTrans, orange) does not quite reach the performance of the models trained on a single MSA. In general, it is encouraging that an MSA Transformer can generate a reasonably close distribution from only 256 input sequences at a time. However, the method scales very poorly and it might take an unreasonable amount of time to generate longer proteins such as kinase.

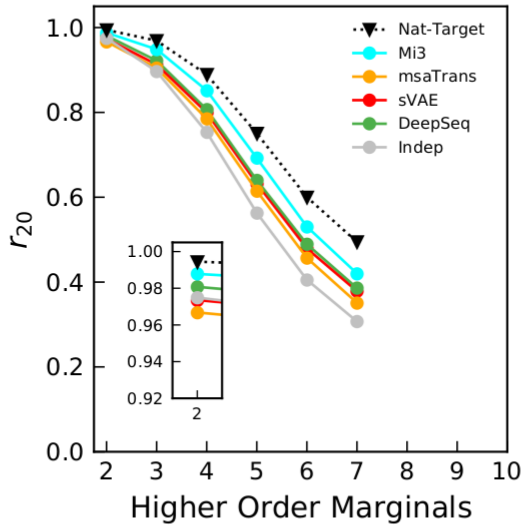

Comparison using the r20 metric of different GPSMs as well as MSA transformer (msaTrans). The GPSMs are fit to the Natural 10K kinase MSA, and MSA tranformer model is a pre-trained model fit to multiple protein family MSAs as described in Ref. \citen{rao_msa_2021}.

We have included these calculations and discussion in Section 13, SI, "MSA Transformer".

Some general comments:
- Figures are nicely done, however, axis-labeling and captioning could be made clearer. It is not always obvious what is being compared in the plots (in particular fig 2) what synthetic model is used where?

We have updated the figure labelling to add subpanel titles and other labels, as the reviewer suggests. See Figures 2, 3, 4, 5, ms.

- The higher order marginal statistics calculation is poorly explained in the main-text. It is not clear to this reader why there should be any correlations for these quantities with the data in the independent sites Model.

We have updated the description of the r20 metric (page 8, ms) and included a new subfigure to better illustrate the r20 calculation, shown below:

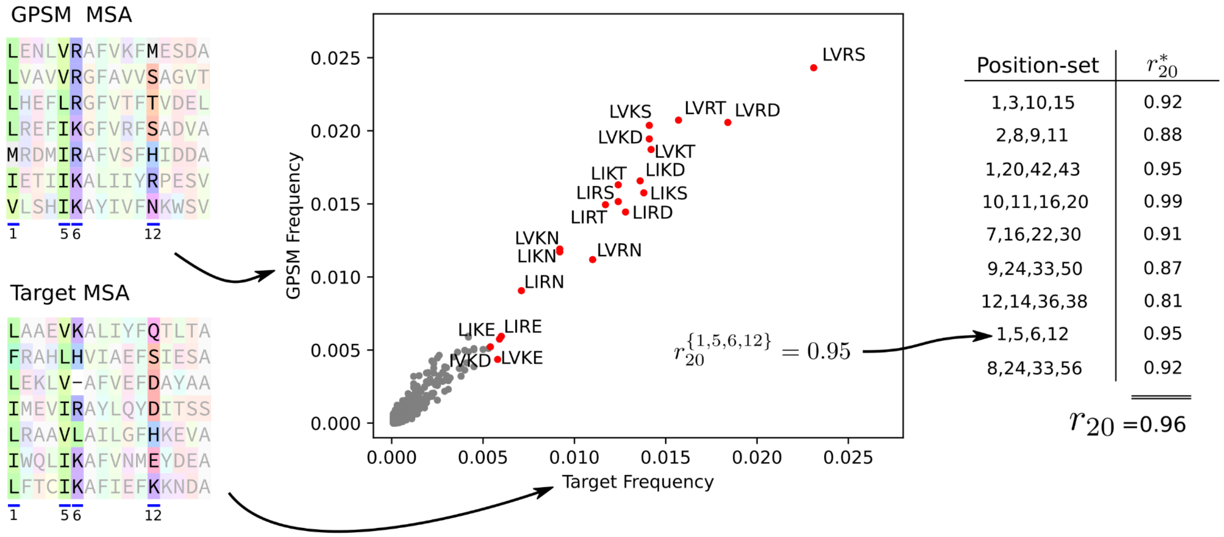

Diagram of r20 calculation for n=4 Left: For a particular set of positions, 1,5,6,12 in this case, the subsequence frequencies for all words at these positions is calculated for the target MSA and the GPSM MSA. Middle: The word frequencies for the two MSAs are compared, and the top-20 most frequent words according to the Target MSA (red points, labelled by word) are used to compute the Pearson-r value between the two sets of MSA frequencies. Right: This procedure is repeated for many different sets-of-positions. The final r20 score is the average of the r across position-sets.

To compute r20, for a given set-of-positions, we determine the top 20 most frequent “words” at those positions according to our target model, and we compare the frequencies of those words according to the target model to those of the GPSM, using a Pearson Correlation score. We have added an additional explanation in SI.

The r20 metric tests whether a model is able to reproduce the n-tuple frequencies (i.e., “word” frequencies), for instance the tuple of characters VALKD may appear with 2% frequency at positions 10, 20, 30, 40, 50. Even a site-independent model makes a non-zero prediction for these word frequencies, giving frequency equal to the product of the single-site frequencies of the character at each position. This explains why even the Indep model is able to give a non-zero r20 score, as it is able to predict word frequencies with limited accuracy, especially if epistasis is rare.

In response to reviewer #1, we also introduced another, similar, metric which may behave more like reviewer #2 expects, such that the Indep model gives 0 for all orders. It is computed in a very similar way to r20 but using the “connected correlations” in place of the word frequencies, and we call this the cc-r20 metric. The connected correlations can be seen as a generalization of Cijαβ to higher orders. See Section 7, SI, as well as the description in the ms (page 9, ms) paragraph beginning “Another potential issue is that the r20 metric does not directly test for covariation”.

- One of the few examples of Potts models actually generating experimentally validated protein sequences and the statistical energy correlating with stability and melting temperature should be cited:

https://onlinelibrary.wiley.com/doi/abs/10.1002/anie.201713220

We have now cited this publication in the first paragraph of the introduction.

- The Monte Carlo estimator the authors use to compute the statistical energy (marginal likelihood of the VAE) is known to converge slowly in some cases. 1000 could be on the low end. The authors should include a plot of the boot-strapped error of this quantity for a few representative sequence as function of the number of samples in the SI.

We had briefly discussed this issue in SI, but have now expanded on this issue and performed a test as the reviewer suggests (Section 2, SI).

Below we show results of a test using the Kinase family, which we have added in the SI. In this test, we obtain 100000 Monte-Carlo samples of the r.h.s. term of equation 1, SI from the sVAE for each of a set of 200 sequences. We then estimate the value of E(S) = - log P(S) for each sequence as the average over these 1,000,000 samples, as in equation 1, SI. We label this estimate using all 100,000 samples E_mu(S).

Then, we repeat the averaged estimate of E(S) but by using a limited number n of the 100,000 samples, giving an estimate E_n(S). For each value n, for each sequence, we repeat this estimation of E_n(S) for k=10000 times for different random choices of the n samples out of the 1,000,000, so in total we have k estimates E_n,k(S) for each sequence for each value n.

We then measure the error relative to the estimate made with all the samples E_mu. We measure the error in two ways: 1. The root-mean-square (RMS) error in the energy, sqrt(<E_n - E_mu>), averaging over all sequences and k, and 2. The maximum observed error, max |E_n - E_mu|, maximized over all sequences and k. We plot both forms of error below.

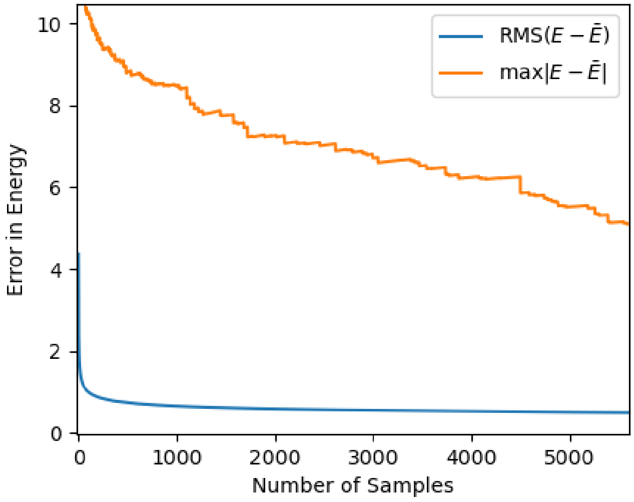

We find that the RMS error in the energy becomes less than 1 even at a very small number of samples, n=101. At n=1000 as we used in the main text, the RMS error in the energy is 0.6 energy units. This is much smaller than the RMS Error in the predicted energy (after subtracting the mean offsets) for the sVAE relative to the Target energy in our synthetic tests, for instance of 78 energy units for figure 4E for our 1M synthetic test. This suggests that the amount of statistical error due to the Monte Carlo sampling is a negligible source of error in our results for sVAE, and that n=1000 samples is sufficient to demonstrate our results in the main text.

The max error line in the plot represents the extremely rare “worst case” scenario. This is the maximum difference we observed for E_n relative to the large-sample estimate E_mu, over all k realizations. This rare worst-case error is higher than the RMS error, and at n=1000 samples is 8 energy units. This is still much smaller than the RMS error of the sVAE relative to the target in our synthetic tests, so our results using n=1000 in the main text should be reliable. The fact that the “worst case” error is significantly larger than the RMS error suggests a rare-event phenomenon. Indeed, we observe (not shown) that, rarely, sVAE generates outlier samples which have a very high likelihood score, which dominate the average in equation 1, SI. This property of sVAE may explain why other studies had difficulty converging the average. Nevertheless, our tests suggest that despite this the error in our estimates of E is small relative to the model error.

This discussion is now included in Section 2, SI.

Reviewers' Comments:

Reviewer #1:
Remarks to the Author:
The authors have carefully revised their manuscript. I appreciate in particular the effort to make the manuscript more broadly accessible thanks to an extended introduction, improved discussions and explained applications beyond GPSM. Even more importantly, the new results obtained both on artificial and protein sequence data support much better the main claims of the paper and make the conclusion substantially more solid and reliable.

The literature on related subjects is huge, and the authors present a very good selection of references. But since they introduced [13] for the experimental verification of generated sequences, they might be more exhaustive on this subject. The seminal experimental paper is Socolich et al. Nature 2005, which does not use explicit GPSM but Ref. [15] shows that their approach is equivalent to sampling from a Potts model. More recently diverse GPSM have been tested for their capacity to generate functional proteins, examples are Russ et al. Science 2020 for Potts models, Hawkins-Hooker et al PLoS CB 2021 for VAE, Repecka et al. Nature Mach. Intell. for GAN. This list may be incomplete.

Reviewer #2:
Remarks to the Author:
Congratulations on a much improved manuscript. I would like to thank you for seriously addressing all my concerns, and that of the other reviewer, in a thorough and rigorous fashion. Although I disagree with your argument about the parameterization of statistical models (continuous/discrete models) I recognize that the focus and contribution of your work lies elsewhere and is valuable in its own respect.

Response to Reviewers

We thank both reviewers again for their insightful suggestions and comments, especially their suggestions for new and interesting tests of higher-order marginals for different GPSMs which strengthened the manuscript.

Our specific responses to the new reviews are below. In summary, we added the citations the reviewers suggested, and added a data availability statement. These changes are highlighted in red in the updated manuscript.

Reviewer #1 (Remarks to the Author):

The authors have carefully revised their manuscript. I appreciate in particular the effort to make the manuscript more broadly accessible thanks to an extended introduction, improved discussions and explained applications beyond GPSM. Even more importantly, the new results obtained both on artificial and protein sequence data support much better the main claims of the paper and make the conclusion substantially more solid and reliable.

Again, we thank the reviewer for suggestions that led to these useful new results now included in the manuscript.

The literature on related subjects is huge, and the authors present a very good selection of references. But since they introduced [13] for the experimental verification of generated sequences, they might be more exhaustive on this subject. The seminal experimental paper is Socolich et al. Nature 2005, which does not use explicit GPSM but Ref. [15] shows that their approach is equivalent to sampling from a Potts model. More recently diverse GPSM have been tested for their capacity to generate functional proteins, examples are Russ et al. Science 2020 for Potts models, Hawkins-Hooker et al PLoS CB 2021 for VAE, Repecka et al. Nature Mach. Intell. for GAN. This list may be incomplete.

As suggested by the reviewer, we have added an appropriate set of citations in the introduction related to experimental tests of GPSM-generated sequences.

Reviewer #2 (Remarks to the Author):

Congratulations on a much improved manuscript. I would like to thank you for seriously addressing all my concerns, and that of the other reviewer, in a thorough and rigorous fashion. Although I disagree with your argument about the parameterization of statistical models (continuous/discrete models) I recognize that the focus and contribution of your work lies elsewhere and is valuable in its own respect.

We appreciate the reviewer's comments. We share the reviewer's interest in questions related to GPSM latent representations and dataset topology, which includes the questions related to continuous/discrete data, and hope to have the opportunity to explore them in the future.
